# Supplementary material for: Libra: scalable k-mer–based tool for massive all-vs-all metagenome comparisons
Source: Gigascience. 2018 Dec 28;8(2):giy165. doi: 10.1093/gigascience/giy165 (PMC6354030; doi:10.1093/gigascience/giy165)
Supplement: GIGA-D-18-00324_Original_Submission.pdf [file giy165_giga-d-18-00324_original_submission.pdf]

GigaScience

Libra: robust biological inferences of global datasets using scalable k-mer based all-vs-all metagenome comparisons

--Manuscript Draft--

**Manuscript Number:**	GIGA-D-18-00324
**Full Title:**	Libra: robust biological inferences of global datasets using scalable k-mer based all-vs-all metagenome comparisons
**Article Type:**	Research
**Funding Information:**	Directorate for Computer and Information Science and Engineering (1640775) — Prof. Bonnie L Hurwitz
**Abstract:**	Background<br><br>Shotgun metagenomics provides powerful insights into microbial community biodiversity and function. Unfortunately, inferences from metagenomic studies are often limited by dataset size and complexity, and are restricted by the availability and completeness of existing databases. De novo comparative metagenomics enables the comparison of metagenomes based on their total genetic content.<br><br>Results<br><br>We developed a novel tool called Libra that performs all-vs-all comparison of metagenomes based on their k-mer-composition. This tool presents three main innovations: the use of a scalable Apache Hadoop framework enabling massive dataset comparison, the use of complex distance metrics allowing precise clustering of metagenomes based on their k-mer content, and a web-based tool imbedded in iMicrobe (http://imicrobe.us) that uses the CyVerse advanced cyberinfrastructure to promote broad use of the tool by the scientific community.<br><br>Conclusions<br><br>A comparison of Libra to equivalent tools using both simulated and real metagenomic datasets, ranging from 80 million to 4.2 billion reads, reveals that numerous methods commonly implemented to reduce compute time for large datasets—such as data reduction, read count normalization, and presence/absence distance metrics—greatly diminish the degree of resolution and robustness of large-scale comparative analyses. In contrast, Libra provides scalable high-resolution comparisons using all reads without biases due to differences in abundance and read depth, enabling global-scale analyses to identify microbial signatures linked to biological processes.
**Corresponding Author:**	Bonnie Hurwitz<br><br>UNITED STATES
**Corresponding Author Secondary Information:**	
**Corresponding Author's Institution:**	
**Corresponding Author's Secondary Institution:**	
**First Author:**	Illyoung Choi, MS
**First Author Secondary Information:**	
**Order of Authors:**	Illyoung Choi, MS
	Alise J. Ponsero, PhD
	Matthew Bomhoff, MS
	Ken Youens-Clark, BA

Powered by Editorial Manager® and ProduXion Manager® from Aries Systems Corporation

	John H. Hartman, PhD
	Bonnie L Hurwitz, PhD
**Order of Authors Secondary Information:**	
**Additional Information:**	
**Question**	**Response**
Are you submitting this manuscript to a special series or article collection?	No
**Experimental design and statistics**<br><br>Full details of the experimental design and statistical methods used should be given in the Methods section, as detailed in our Minimum Standards Reporting Checklist. Information essential to interpreting the data presented should be made available in the figure legends.<br><br>Have you included all the information requested in your manuscript?	Yes
**Resources**<br><br>A description of all resources used, including antibodies, cell lines, animals and software tools, with enough information to allow them to be uniquely identified, should be included in the Methods section. Authors are strongly encouraged to cite Research Resource Identifiers (RRIDs) for antibodies, model organisms and tools, where possible.<br><br>Have you included the information requested as detailed in our Minimum Standards Reporting Checklist?	Yes
**Availability of data and materials**<br><br>All datasets and code on which the conclusions of the paper rely must be either included in your submission or deposited in publicly available repositories (where available and ethically appropriate), referencing such data using	Yes

Powered by Editorial Manager® and ProduXion Manager® from Aries Systems Corporation

a unique identifier in the references and in the “Availability of Data and Materials” section of your manuscript.<br><br>Have you have met the above requirement as detailed in our Minimum Standards Reporting Checklist?	

Manuscript

Click here to download Manuscript Gigascience_Libra_Manuscript.docx

Title: Libra: robust biological inferences of global datasets using scalable k-mer based all-vs-all metagenome comparisons

Authors: Illyoung Choi[1], Alise J. Ponsero[2], Matthew Bomhoff[2], Ken Youens-Clark[2], John H. Hartman[1*], and Bonnie L. Hurwitz[2,3*]

Affiliations:

[1]Department of Computer Science, University of Arizona, Tucson, Arizona

[2]Department of Biosystems Engineering, University of Arizona, Tucson, Arizona

[3]BIO5 Institute, University of Arizona, Tucson, Arizona

Corresponding Author:

Bonnie L. Hurwitz bhurwitz@email.arizona.edu

ABSTRACT

Background: Shotgun metagenomics provides powerful insights into microbial community biodiversity and function. Unfortunately, inferences from metagenomic studies are often limited by dataset size and complexity, and are restricted by the availability and completeness of existing databases. *De novo* comparative metagenomics enables the comparison of metagenomes based on their total genetic content.

Results: We developed a novel tool called Libra that performs all-vs-all comparison of metagenomes based on their k-mer-composition. This tool presents three main innovations: the use of a scalable Apache Hadoop framework enabling massive dataset comparison, the use of complex distance metrics allowing precise clustering of metagenomes based on their k-mer content, and a web-based tool imbedded in iMicrobe (http://imicrobe.us) that uses the CyVerse advanced cyberinfrastructure to promote broad use of the tool by the scientific community.

Conclusions: A comparison of Libra to equivalent tools using both simulated and real metagenomic datasets, ranging from 80 million to 4.2 billion reads, reveals that numerous methods commonly implemented to reduce compute time for large datasets—such as data reduction, read count normalization, and presence/absence distance metrics—greatly diminish the degree of resolution and robustness of large-scale comparative analyses. In contrast, Libra provides scalable high-resolution comparisons using all reads without biases due to differences in abundance and read depth, enabling global-scale analyses to identify microbial signatures linked to biological processes.

Keywords: metagenomics, Hadoop, k-mer, distance metrics, clustering

INTRODUCTION

Over the last decade, scientists have generated petabytes of genomic data to uncover the role of microbes in dynamic living systems. Yet to understand the underlying biological principles that guide the distribution of microbial communities, massive ‘omics datasets need to be compared with environmental factors to find linkages across space and time. One of the greatest challenges in these endeavors has been in documenting and analyzing unexplored genetic diversity in wild microbial communities. For example, fewer than 60% of 40 million non-redundant genes from the Global Ocean Survey (GOS) and the Tara Oceans Expeditions match known proteins in bacteria [1,2]. Other microorganisms such as viruses or pico- eukaryotes that are important to ocean ecosystems are even less well defined (e.g. < 7% of reads from viromes match known proteins [3]). This is largely due to the fact that reference genomes for these organisms do not exist in public data repositories and genome-sequences from metagenomic data await better taxonomic and functional definition. As a result, even advanced tools such as k-mer based classifiers that rapidly assign metagenomic reads to known microbes (Table 1) miss “microbial dark matter” that comprises a significant proportion of metagenomes.

Table 1.

Tool	Area*	Method	Platform	Command line	Parallelized	Scalable**	Web-enabled	Cyber-infrastructure	Cited by	Year
**Libra**	**MG**	Pairwise distance calculation	**Hadoop**	**X**	**X**	**X**	**X**	**X**	**current study**	
Compareads	MG	Pairwise distance calculation	single server	X					35	2012
Commet	MG	Pairwise distance calculation	single server	X					30	2014
Mash	G/MG	Pairwise distance calculation	single server	X					157	2016
Simka	MG	Pairwise distance calculation	HPC***	X	X				18	2016
NBC	MG	Taxonomic profiling	singer server	X					168	2010
Kraken	MG	Taxonomic profiling	singer server	X					785	2014
FOCUS	MG	Taxonomic profiling	singer server	X			X		49	2014
Clark	MG	Taxonomic profiling	singer server	X					176	2015

Metaphlan2	MG	Taxonomic profiling	singer server	X			227	2015
Metafast	MG	Taxonomic profiling	single server	X			19	2016
Centrifuge	MG	Taxonomic profiling	single server	X			78	2016
Jellyfish	G/MG	K-mer counting	single server	X			746	2011
BioPig	G/MG	K-mer counting	Hadoop	X	X	X	97	2013
Bloomfish	G/MG	K-mer counting	Hadoop	X	X	X	2	2017
Myrna	G	Differential gene expression	Hadoop	X	X	X	331	2010
Eoulsan	G	Differential gene expression	Hadoop	X	X	X	90	2012
Cloud RSD	G	Ortholog detection	Hadoop	X	X	X	120	2010
CloudBLAST	G	Read mapping (ref db)	Hadoop	X	X	X	362	2008
Cloudburst	G	Read mapping (ref genome)	Hadoop	X	X	X	711	2009
Crossbow	G	Variant detection	Hadoop	X	X	X	501	2009

* MG = metagenomics; G = genomics

** Scalability is defined as reliable distributed high-performance computing framework

*** High-performance computer

***De novo* comparative metagenomics offers a path forward.** In order to examine the complete genomic content, metagenomic samples can be compared using their sequence signature (or frequency of k-mers; Table 1). This approach relies on three core tenets of k-mer-based analytics: (i) closely related organisms share k-mer profiles and cluster together, making taxonomic assignment unnecessary [4,5], (ii) k-mer frequency is correlated with the abundance of an organism [6], and (iii) k-mers of sufficient length can be used to distinguish specific organisms [7]. In 2012, the Compareads [8] method was proposed, followed by Commet [9]. Both of these tools compute the number of shared reads between metagenomes using a k-mer-based read similarity measure. The number of shared reads between datasets is then used to compute a Jaccard distance between samples. Given the computational intensity of all-vs-all sequence analysis, several other methods have been employed to reduce the dimensionality of metagenomes and speed up analyses by creating unique k-mer sets and computing the genetic distance between pairs of metagenomes, such as MetaFast [10] and Mash [11]. The fastest of these methods, Mash, indexes samples by unique k-mers to create size-reduced sketches, and compares these sketches using the min-Hash algorithm [12] for computing a genetic distance

using Jaccard similarity. Yet, the tradeoff for speed is that samples are reduced to a subset of unique k-mers (1k by default) that lack information on k-mer abundance in the samples. Further, given that Mash uses Jaccard similarity only the genetic distance between samples is accounted for (or genetic content in microbial communities) without considering abundance (dominant vs rare organisms in the sample) which is central to microbial ecology and ecosystem processes.

Recently, SIMKA [13] was developed to compute a distance matrix between metagenomes by dividing the input datasets into abundance vectors from subsets of k-mers, then rejoining the resulting abundances in a cumulative distance matrix. The methodology can be parallelized to execute the analyses on a high-performance compute cluster (HPC). SIMKA also provides various ecological distance metrics to let the user choose the metric most relevant to their analysis. However, the computational time varies based on the distance metric, where simple distances scale linearly and complex distances metrics scale quadratically as additional samples are added [13]. Moreover, SIMKA normalizes datasets in an all-vs-all comparison by reducing the depth of sequencing for all samples to the least common denominator, therefore decreasing the resolution of the datasets. Lastly, computing k-mer analytics using HPC is subject to reduced fault tolerance for massive datasets.

Scaling sequence analysis using big data analytics via Hadoop. Hadoop is an attractive platform for performing large-scale sequence analysis because it provides a distributed file system and distributed computation for analyzing massive amounts of data. Hadoop clusters are comprised of commodity servers so that the processing power increases as more computing resources are added. Hadoop also offers a high-level programming abstraction based on MapReduce that greatly simplifies the implementation of new analytical tools. Programmers do not need specialized training in distributed systems and networking to implement distributed programs using Hadoop. Hadoop also provides fault-tolerance by default. When a Hadoop node fails, Hadoop reassigns the failed node’s tasks to another node containing a redundant copy of

the data those jobs were processing. This differs from HPC where schedulers track failed nodes and either restart the failed computation from the most recent checkpoint, or from the beginning if checkpointing wasn't used. Thus, using a Hadoop infrastructure ensures that computations and data are protected even in the event of hardware failures. These benefits have led to new analytic tools based on Hadoop, making Hadoop a de facto standard in large-scale data analysis. In metagenomics, the development of efficient and inexpensive high-throughput sequencing technologies has led to a rapid increase of the amount of sequence data for studying microbes in diverse environments. However, no Hadoop-enabled comparative metagenomics tools currently exist.

Spark [14] is increasingly popular for scientific data analysis [15] because of its outstanding performance provided by fast in-memory processing. Although Libra is currently implemented on Hadoop, Libra can be easily ported to Spark because both Hadoop and Spark have similar interfaces for data processing and partitioning. For example, Resilient Distributed Datasets (RDD) can be partitioned and distributed over a Spark cluster using Libra's k-mer range partitioning. RDDs are memory-resident, allowing Spark to significantly improve the performance of Libra's k-mer counting and distance matrix computation by avoiding slow disk I/O for intermediate data. Nevertheless, we implemented Libra using Hadoop because Spark requires much more RAM than Hadoop, significantly increasing the cost of the cluster.

Existing big data algorithms compare reads to limited genomic reference data. Recent progress has been made in translating bioinformatics algorithms to big data architectures to overcome scalability issues for genomic but not metagenomic applications (Table 1). Thus far, these algorithms compare large-scale NGS datasets to reference genomic datasets and replace computationally intensive algorithms such as sequence alignment [16], genetic variant detection [17,18], or short read mapping [19–22]. For example, BlastReduce and CloudBurst are parallel sequence mapping tools based on Apache MapReduce [20,21]. These tools, however, implement a query-to-a-reference approach that is inefficient for all-vs-all analyses of reads from

metagenomes. Other algorithms such as BioPig [23] and Bloomfish [24] generate an index of sequence data for later partial sequence search and k-mer counting using MapReduce [25]. These tools, however, adopt a suffix array approach similar to traditional bioinformatics tools that is inefficient in reading and indexing data on a distributed file system such as Hadoop, thus reducing performance. Moreover, neither tool offers an end-to-end solution for comparing metagenomes consisting of: data distribution on a Hadoop cluster, k-mer indexing and counting, distance computation, and visualization. Finally, none of these tools are enabled in an advanced cyberinfrastructure where users can compute analyses in a simple web-based platform that offers compute, data storage, and analysis tools.

Libra: a tool for scalable all-vs-all sequence analysis in an advanced cyberinfrastructure

Here, we describe a scalable algorithm called Libra that is capable of performing all-vs-all sequence analysis using MapReduce on the Apache Hadoop platform. We demonstrate for the first time that Hadoop can be applied to all-vs-all sequence comparisons of large-scale metagenomic datasets comprised of mixed microbial communities. We present a new distance metric for comparing datasets using Cosine Similarity [34] to consider genetic distance and microbial abundance simultaneously, along with widely accepted distance metrics in biology such as Bray-Curtis [35] and Jensen-Shannon [36]. We validate this new distance metric using simulated metagenomes to show that Libra has exceptional sensitivity in distinguishing complex mixed microbiomes. Next, we show Libra's ability to distinguish metagenomes by both community composition and abundance using 48 samples (16S rRNA and WGS) from the human microbiome project (HMP) across diverse body sites, and compare the results to Mash and SIMKA. Finally, we show that Libra can scale to massive global-scale datasets by examining viral diversity in 43 Tara Ocean Viromes (TOV) from the 2009-2011 Expedition [27] that represent 26 sites containing about 4.2 billion reads. The resulting data demonstrate that Libra provides accurate, efficient, and scalable compute for comparative metagenomics that can be used to discern global patterns in microbial ecology.

To promote the broad use of the Libra algorithm we developed a web-based tool in iMicrobe (http://imicrobe.us), where users can run Libra using data in their free CyVerse [28,29] account or use datasets that are integrated into the iMicrobe Data Commons. These analyses are fundamental for determining relationships among diverse metagenomes to inform follow-up analyses on microbial-driven biological processes.

DATA DESCRIPTION

Staggered mock community. We performed metagenomic shotgun sequencing on a staggered mock community obtained from the Human Microbiome Consortium (HM-277D). The staggered mock community is comprised of genomic DNA from genera commonly found on or within the human body, consisting of 1,000 to 1,000,000,000 16S rRNA gene copies per organism per aliquot. The resulting DNA was subjected to whole genome sequencing as follows. Mixtures were diluted to a final concentration of 1 nanogram/microliter and used to generate whole genome sequencing libraries with the Ion Xpress Plug Fragment Library Kit and manual #MAN0009847, revC (Thermo Fisher Scientific, Waltham, MA, USA). Briefly, 10 nanograms of bacterial DNA was sheared using the Ion Shear enzymatic reaction for 12 min and Ion Xpress barcode adapters ligated following end repair. Following barcode ligation, libraries were amplified using the manufacturer's supplied Library Amplification primers and recommended conditions. Amplified libraries were size selected to ~ 200 base pairs using the Invitrogen E-gel Size Select Agarose cassettes as outlined in the Ion Xpress manual and quantitated with the Ion Universal Library quantitation kit. Equimolar amounts of the library were added to an Ion PI Template OT2 200 kit V3. The resulting templated beads were enriched with the Ion OneTouch ES system and quantitated with the Qubit Ion Sphere Quality Control kit (Life Technologies) on a Qubit 3.0 fluorometer (Qubit, NY, NY, USA). Enriched templated beads were loaded onto an Ion PI V2 chip and sequenced according to the manufacturer's protocol using the Ion PI Sequencing 200 kit V3 on a Ion Torrent Proton sequencer. The sequence data

comprised of ~80 million reads have been deposited to the NCBI Sequence Read Archive under accession SRP115095 under project accession PRJNA397434.

Simulated data derived from the staggered mock community*.* The resulting sequence data from the staggered mock community (~80 million reads) were used to develop simulated metagenomes to test the effects of varying read depth, and composition and abundance of organisms in mixed metagenomes. To examine read depth (in terms of raw read counts and file size), we used the known staggered mock community abundance profile to generate an artificial metagenome using GemSim [30] of 2 million reads (454 sequencing) and duplicated the dataset 2x, 5x and 10x. We also simulated the effects of sequencing a metagenome more deeply using GemSim [30] to generate simulated metagenomes with 0.5, 1, 5, and 10 million reads based on the relative abundance of organisms in the staggered mock community. Next, we developed four simulated metagenomes to test the effect of changing the dominant organism abundance and genetic composition including: 10 million reads from the staggered mock community (mock 1), the mock community with alterations in a few abundant species (mock 2), the mock community with many alterations in abundant species (mock 3), and mock 3 with additional sequences from archaea to further alter the genetic composition (mock 4) as described in Supplemental Table 1. All simulated datasets are available in iMicrobe (http://imicrobe.us).

Human microbiome 16S rRNA gene amplicons and WGS reads. Human microbiome datasets were downloaded from the NIH Human microbiome project [31] including 48 samples from 5 body sites including: urogenital (posterior fomix), gastrointestinal (stool), oral (buccal mucosa, supragingival plaque, tongue dorsum), airways (anterior nares), and skin (retroauricular crease left and right; Supplemental Table 2). Matched datasets consisting of 16S rRNA reads, WGS reads, and WGS assembled contigs were downloaded from the 16S trimmed dataset and the HMIWGS/HMASM dataset respectively. For the WGS reads dataset, the analysis was run on the paired 1 read file.

Tara ocean viromes. Tara oceans viromes were downloaded from European Nucleotide Archive (ENA) at EMBL and consisted of 43 viromes from 43 samples at 26 locations across the world's oceans collected during the Tara Oceans (2009-2012) scientific expedition (Supplemental Table 3; [27]). Metadata for the samples was downloaded from PANGAEA [32]. These samples were derived from multiple depths including: 16 surface samples (5-6 meters), 18 deep chlorophyll maximum samples (DCM; 17-148 meters), and one mesopelagic sample (791 meters). Quality control procedures were applied according to methods described by Brum and colleagues [27].

RESULTS AND DISCUSSION

Libra computational strategy. Libra uses Hadoop MapReduce to perform massive all-vs-all sequence comparisons between next-generation sequence (NGS) datasets. Libra is designed to estimate genetic distance accurately without sacrificing performance. Instead, scalable algorithms and efficient resource usage make it feasible to perform all-vs-all comparisons on large datasets.

Libra performs all-vs-all distance comparisons using a sweep line algorithm (https://en.wikipedia.org/wiki/Sweep_line_algorithm). Naively, all-vs-all comparisons would require a total of [illegible] × ([illegible] − 1)/2 comparisons between [illegible] samples. Using a sweep line algorithm, Libra can perform these comparisons in a single pass (Supplemental Figure 1). Libra maximizes cluster efficiency using a load balancing algorithm inspired by Terabyte Sort [33] to distribute the workload evenly over the Hadoop cluster. Highly parallelizable inverted index construction and distance matrix computation algorithms enable Libra to scale to any size NGS dataset (often millions of reads), and perform any number of comparisons across datasets, making global ecosystem-level analyses possible.

Libra distance calculation. Libra uses a vector space model to compute the distance between two NGS datasets. In this model each sample is represented by a vector, each dimension of which corresponds to a unique k-mer. Each component of a vector indicates the weight given to the corresponding k-mer in the distance computation. For example, using the frequency (the raw count) of a k-mer as its weight and using 4-mers, the vector <2,4,0,...> indicates that a k-mer 'aaaa' has a weight of two and a k-mer 'aaac' has a weight of four in the sample, etc. The more weight, the more important the k-mer.

The distance between two samples can now be measured by comparing their vectors using a distance metric. Libra provides three distance metrics — Cosine Similarity [34], Bray-Curtis [35] and Jensen-Shannon [36]. In this paper, we demonstrate Cosine Similarity as the default distance metric given that it had the shortest runtime for all distances (see Methods).

Cosine Similarity determines an estimate of the genetic distance between samples by the angle between the two vectors. The larger the angle, the larger the distance. The cosine is one when the angle is zero (i.e. the vectors are identical except for their magnitude) and less than one otherwise (see Supplemental Methods for a detailed description).

The cosine of the angle does not depend on the magnitude (length) of the vectors. This is advantageous in comparing samples with different sizes of samples (or sequencing depth). For example, if there are two samples with the same composition of k-mers but one has k-mers with double the frequency than the other, their vectors will have same angles so that their cosine similarity will one.

Libra implementation. We implemented Libra on the Hadoop MapReduce platform. This allows Libra to run on any standard Hadoop 2.3 implementation, while taking advantage of the scalability and fault-tolerance features provided by Hadoop. Hadoop allows robust parallel computation over distributed computing resources via its simple programming interface called

MapReduce, while hiding much of the complexity of distributed computing (e.g. node failures). Taking advantage of Hadoop MapReduce, Libra can scale to larger input datasets and more computing resources. Furthermore, many cloud providers such as Amazon and Google offer Hadoop clusters on a pay-as-you-go basis, allowing scientists to scale their Libra computations to match their datasets and budgets.

Libra is implemented using three different MapReduce jobs — 1) k-mer histogram construction, 2) inverted index construction, and 3) distance matrix computation. Figure 1 shows a workflow of the Libra algorithm.

Figure 1. The Libra Workflow.

Libra consists of three MapReduce jobs (yellow boxes) — 1) k-mer histogram construction, 2) inverted index construction and 3) distance matrix computation. k-mer histograms are first constructed for input samples to balance workloads over the Hadoop cluster during the subsequent jobs. Inverted indices are constructed per a group of samples in parallel by partitioning k-mer ranges. An index chunk is produced from each partition and an inverted index is constructed from multiple index chunks. During the distance matrix computation, partial contributions are computed within a partition and accumulated to produce the final distance matrix.

Libra constructs a k-mer histogram of the input samples for load-balancing. A separate Map task is spawned for every data block in the input sample files to calculate the k-mer histogram for each sample. Thus, the k-mer histogram of the input samples is computed in parallel by running multiple Map tasks and a Reduce task that combines their results.

Libra performs the inverted index construction in parallel. In the Map phase, a separate Map task is spawned for every data block in the input sample files. Each Map task generates k-mers from the sequences stored in a data block then passes them to the Reduce tasks. In the

Reduce phase, the I/O and computation is split by partitioning the k-mer space using the k-mer histograms computed in the first phase (Supplemental Figure 2). A separate Reduce task is spawned for every partition and a custom Partitioner routes the produced k-mers to Reduce tasks by their k-mer ranges. Each Reduce task then counts k-mers it receives and produces an index chunk. As a result, each index chunk is stored as a separate file in the Hadoop MapFile format. The MapFile is well-suited for Libra as it is designed to store key-value pairs in key order, and supports binary search of the keys.

In the distance matrix computation, the work is split by partitioning the k-mer space in the beginning of a MapReduce job. The k-mer histogram files for input samples are loaded and merged, and the k-mer space is partitioned according to the k-mer distributions. A separate Map task is spawned for each partition to perform the computation in parallel. As a result, each task produces an output file containing partial contributions to the score matrix. At the end of the job, Libra merges the partial contributions from the files and produces the complete distance matrix.

Advanced cyberinfrastructure for Libra in iMicrobe. To improve access to Libra we made it available at iMicrobe (https://www.imicrobe.us). A researcher with a CyVerse account can run Libra on iMicrobe by filling-out a simple web form specifying the input files and parameters. Input files are selected from the CyVerse Data Store where they have either been uploaded by the user to their home directory or are part of the iMicrobe Data Commons. When a job is submitted, the user is presented with the status of the job, and on completion the output files and visualization of results. To deploy Libra on iMicrobe, we developed a job dispatch service to automate execution of Libra on a University of Arizona Hadoop cluster. The service is written in NodeJS and accepts a JSON description of the job inputs and parameters, stages the input files onto the UA Hadoop cluster, executes Libra with the given parameters, and transfers the resulting output files to the user's home directory in the CyVerse Data Store. The service

provides a RESTful interface that mimics the Agave API Jobs service and is secured using an Agave OAuth2 token. Source code is located at https://github.com/hurwitzlab/occ-plan-b.

Cosine similarity allows for an accurate and normalized comparison of metagenomes.

Jaccard and Bray-Curtis distance have been extensively used to compare metagenomes based on their sequence signature [10,11,13]. While Mash only computes the Jaccard distance between samples, Simka and Libra implement several classical ecology distances allowing the user to choose the best-suited distance for the considered dataset [13]. Moreover, Libra implements a new distance metric, the cosine similarity. Users can also weight k-mers based on their abundance in Libra (using boolean weighting, natural weighting and logarithmic weighting) to account for differences in microbial community composition and sequencing effort as detailed below.

We tested these effects by varying: (1) the size of the datasets, (2) depth of sequencing, (3) the abundance of dominant microbes in the community, and (4) genetic composition of the community by adding in an entirely new organism (in our case we added archaea). We constructed simulated metagenomes and compared Libra's distance based on the cosine similarity against those from Mash and SIMKA. Simulated datasets were derived from genomic DNA from a staggered mock community of bacteria obtained from the human microbiome consortium and sequenced deeply using the Ion Torrent sequencing platform (80 million reads, see Methods).

First, we examined the effect of the size of the dataset by using GemSim [30] to obtain a simulated metagenome composed of 1 million reads from the mock community and duplicating that dataset 2x and 10x. Overall, we found that altering the size of the metagenome (by duplicating the data) had no effect on the distance between metagenomes for Mash, SIMKA, or Libra. In each case the distance of the duplicated datasets to the 1x mock community was less than 0.0001 (data not shown).

Because metagenomes don’t scale exactly with size and instead have an increasing representation of low-abundance organisms, we created a second simulated dataset from the mock community using GemSim [30] 0.5, 1, 5, and 10 million reads (454 sequencing) to mimic the effect of sequencing more deeply. Given the abundance of organisms in the mock community, the 0.5 M read dataset is mainly comprised of dominant species. With increased sequencing depth (1, 5, and 10 M reads) additional species are added relative to their abundance in the mock community. Overall, sequencing depth has little effect on the distance between samples in Mash and Libra (natural weighting), whereas SIMKA shows no changes between samples when using Jaccard and Bray-Curtis distances (Figure 2A). Indeed, SIMKA normalization is implemented as follows: the smallest sample from the dataset is determined and its number of sequences is used to compare the samples (in this experiment, all mock communities were compared based on the first 0.5 million reads). These results suggest that Libra (natural weighting) and Mash are appropriate for comparing datasets at different sequencing depths, whereas using SIMKA could lead to undesired effects.

Figure 2. Analysis of artificial metagenomes using Mash, SIMKA and Libra.

A. Distance to staggered mock community artificial metagenome composed of 10 million reads (mock1 10M), for artificial metagenomes of same community sequenced at various depth. Artificial metagenomes were obtained using GemSim and the known abundance profile of the staggered mock community (see Supplemental Table 1). In order to mimic various sequencing depth, the artificial metagenomes were generated at 0.5, 1, 5 or 10 million reads (noted mock1 0.5M; mock1 1M; mock1 5M; mock1V2 10M). The distances between the 4 artificial metagenomes and a 10 million read artificial metagenome (mock1 10M) were computing using Mash, SIMKA (Jaccard and Bray-curtis distance) and Libra (natural weighting).
B. Distance to staggered mock community artificial metagenome (mock 1), for artificial

metagenomes from increasingly distant communities. The mock 1 relies on the known abundance profile from the staggered mock community. The mock 2 community profile was obtained by randomly inverting 3 species abundance from mock 1 profile. The mock 3 profile was obtained by randomly inverting 2 species abundances from mock 2 profile. Finally, mock 4 profile was obtained by adding high abundance archeal genomes not present in any the other mock communities. Artificial metagenomes were generated using GemSim at 10 million reads. The distance between the mock 1 community to mock 2, mock 3, mock 4 and a replicate community (mock1 V2) was computed using Mash, SIMKA (Jaccard and Bray-curtis distance) and LIBRA (cosine distance, natural and logarithmic weighting).

In addition to natural variation in population-level abundances, artifacts from sequencing can result in high-abundance k-mers. Libra allows users to select the optimal methodology for weighting high abundance k-mers in their datasets including boolean, natural, and logarithmic. These options for weighting k-mers are important for different biological scenarios as described below and shown in simulated datasets. To examine the effect of weighting, we compared and contrasted the natural and logarithmic weight in Libra, with other distances obtained from Mash and SIMKA (Jaccard and Bray-Curtis). We also examined the effect of adding an entirely new species by spiking a simulated dataset with sequences derived from archaea (that were not present in the mock community). The simulated datasets were comprised of the staggered mock community (mock 1), the mock community with alterations in a few abundant species (mock 2), the mock community with many alterations in abundant species (mock 3), and mock 3 with additional sequences from archaea to alter the genetic composition of the community (mock 4; see Supplemental Table 1). The resulting data showed that Libra (logarithmic weighting) shows a stepwise increase in distance among the mock communities (Figure 2B). This suggests that logarithmic weighting in Libra allows for a comparison of distantly related microbial communities. Mash also shows a stepwise distance between communities, but is

compressed relative to Libra, making differences less distinct. SIMKA (Bray-Curtis and Jaccard) and Libra (cosine distance, natural weighting) reach the maximum difference between mock communities 3 and 4 (Figure 2B). This indicates that these distances are more appropriate when comparing metagenomes with small fluctuations in the community (e.g., data from a time-series analysis), whereas Libra (cosine distance, logarithmic weighting) can be used to distinguish metagenomes that vary in both genetic composition and abundance over a wide-range of species diversity by dampening the effect of high-abundance k-mers. Because of this important difference, we used the cosine distance with the logarithmic weighting in all subsequent analyses. Cosine distance also provided the fastest computation for complex distance metrics (see Methods).

Libra accurately profiles differences in bacterial diversity and abundance in amplicon and WGS datasets from the human microbiome.

Microbial diversity is traditionally assessed using two methods: the 16S rRNA gene to classify bacterial and archaeal groups at the genus to species level, or whole genome shotgun sequencing (WGS) for finer taxonomic classification at the species or subspecies level. Further, WGS datasets provide additional information on functional differences between metagenomes. Here we compare and contrast the effect of different algorithmic approaches (Mash vs Libra vs SIMKA), distance metric (Libra vs SIMKA), data type (16S rRNA vs WGS), and sequence type (WGS reads vs assembled contigs) in analyzing data from 48 samples across 8 body sites from the Human Microbiome Project. Specifically, we examine matched datasets (16S rRNA reads, WGS reads, and WGS assembled contigs) classified as urogenital (posterior fomix), gastrointestinal (stool), oral (buccal mucosa, supragingival plaque, tongue dorsum), airways (anterior nares), and skin (retroauricular crease left and right; Supplemental Table 2).

Because the HMP datasets represent microbial communities, abundant bacteria will have more total read counts than rare bacteria in the samples. Thus, each sample can vary by both taxonomic

composition (the genetic content of taxa in a sample) and abundance (the relative proportion of those taxa in the samples). Importantly, the 16S rRNA amplicon dataset is useful in showing how well each algorithm performs in detecting and quantifying small-scale variation for single a gene at the genus-level, whereas the WGS dataset demonstrates the effect of including the complete genetic content and abundance of organisms at the species-level in a community [37]. Also, we examine differences in each algorithm when read abundance is excluded using assembled contigs that only represent the genetic composition of the community.

Using the 16S rRNA reads, both Mash and Libra clustered samples by broad categories but not individual body-sites (Figure 3A and B). Similar to what is described in previous work [13], samples from the airways and skin co-cluster, whereas other categories including urogenital, gastrointestinal, and oral are distinct [13]. These results indicate that limited variation in the 16S rRNA gene may only allow for clustering for broad categories. Further, the Mash algorithm shows lower overall resolution (Figure 3A) as compared to Libra (Figure 3B). Indeed, amplicon sequencing analysis is not an intended use of Mash, given that it reduces the dimensionality of the data by looking at presence/absence of unique k-mers, whereas Libra examines the complete dataset accounting for both composition in organisms and their abundance. In contrast, SIMKA (Jaccard-ab and Bray-Curtis) failed to cluster samples by broad categories: some skin samples are found associated with stool and formix samples (Figure 3C and D). Moreover, SIMKA Jaccard-ab fails to cluster the mouth samples together (Figure 3C). This result suggests that applying SIMKA and these well-used distance metrics are not appropriate for these datasets.

Figure 3. Clustering of HMP 16S rRNA datasets using Mash, Libra and SIMKA.

48 Human metagenomic samples from the HMP projects clustered by Mash (A), Libra (B) or SIMKA using Jaccard-ab (C) and Bray-Curtis distances (D) from 16s sequencing runs. The samples were clustered using Ward’s method on their distance scores. Heat maps illustrate the

pairwise dissimilarity between samples, scaled between 0 (green) and 1 (red). A key below the heatmap colors the samples by body sites.

When using WGS reads, both Mash and Libra show enhanced clustering by body-site (Figure 4A and B), however Mash shows decreased resolution (Figure 4A) as compared to Libra (Figure 4B). Again, these differences reflect the effect of using all of the read data (Libra) rather than a subset (Mash). Importantly, the Libra algorithm also depends on read abundance that provides increased resolution for interpersonal variation as seen in skin samples (Figure 4B). Similar to the 16S rRNA datasets, SIMKA (Jaccard-ab and Bray-Curtis) failed to cluster the samples by body site, where some skin and stool samples cluster with formix samples (Figure 4C and D). Similarly, SIMKA Jaccard-ab also fails to cluster the mouth samples together (Figure 4C). Overall SIMKA shows an enhanced clustering by body-site using WGS data compared to the 16S rRNA data using these distance metrics, however the clustering is still not accurate.

Figure 4. Clustering of WGS samples using Mash, and Libra and SIMKA.

48 Human metagenomic samples from the HMP projects clustered by Mash (A), Libra (B) or Simka using Jaccard-ab (C) and Bray-Curtis distances (D) from whole genome shotgun sequencing runs. The samples were clustered using Ward's method on their distance scores. Heat maps illustrate the pairwise dissimilarity between samples, scaled between 0 (green) and 1 (red). A key below the heatmap colors the samples by body sites.

When abundance is taken out of the equation by using assembled contigs (Supplemental Figure 3) Mash performs well in clustering distinct body sites whereas Libra shows discrepancies and less overall resolution. Thus, Libra requires reads rather than contigs to perform accurately and obtain high-resolution clustering (Figure 4). SIMKA (Jaccard-ab and Bray-Curtis) was not able to distinguish any assembled datasets and scored all sample-to-sample distances to the maximum, even considering presence-absence distance metric proposed by SIMKA (data not shown). This

phenomenon may be explained by the normalization method used by SIMKA, which does not provide enough data to compare the samples when normalized by the smallest number of contigs (in our dataset 69).

Libra allows for ecosystem-scale analysis: clustering the Tara ocean viromes to unravel global patterns.

To demonstrate the scale and performance of the Libra algorithm, we analyzed 43 Tara Ocean Viromes (TOV) from the 2009-2011 Expedition [27] representing 26 sites, 43 samples, and 4.2 billion reads from the global ocean (see methods). Phages (viruses that infect bacteria) are abundant in the ocean [38] and can significantly impact environmental processes through host mortality, horizontal gene transfer, and host-gene expression. Yet, how phages change over space and time in the global ocean and with environmental fluxes is just beginning to be explored. The primary challenge is the majority of reads in viromes (often > 90%) do not match known proteins or viral genomes [3] and no conserved genes like the bacterial 16S rRNA gene exist to differentiate populations. To examine known and unknown viruses simultaneously, viromes are best compared using sequence signatures to identify common viral populations. Two approaches exist to cluster viromes based on sequence composition. The first approach uses protein clustering to examine functional diversity in viromes between sites [3,27,39]. Protein clustering, however, depends on accurate assembly and gene finding that can be problematic in fragmented and genetically diverse viromes [40]. Further, assemblies from viromes often only include a fraction of the total reads (e.g., only ⅓ in TOV [27]). To examine global viral diversity in the ocean using all of the reads we examined TOV using Libra. The complete pairwise analysis of ~4.2 billion reads in the TOV dataset [27] finished in 18 hours using a 10-node Hadoop cluster (see Methods and Table 2). Importantly, Libra exhibits remarkable performance in computing similarity scores, wherein k-mer matches for all TOV completed within 1.5 hours (Table 2). This step usually represents the largest computational

bottleneck for bioinformatics tools that compute pairwise distances between sequence pairs for applications such as hierarchical sequence clustering [41–44].

Table 2. Execution times for the Libra based on the Tara Ocean Virome (TOV) dataset.

Stage	Execution Time
Preprocessing (k-mer histogram construction + Inverted index construction)	16:32:55
Distance matrix computation	1:24:27
Total	17:57:22

Overall, we found that viral populations in the ocean are largely structured by temperature in four gradients (Figure 5) similar to their bacterial hosts [2]. Interestingly, samples from different Longhurst Provinces but the same temperature gradient cluster together. Also, water samples from the surface (SUR) and deep chlorophyll maximum (DCM) at the same station, cluster more closely together than samples from the same depth at nearby sites (Figure 5). Also noteworthy, samples that were derived from extremely cold environments (noted as C0 in Figure 5) lacked similarity to all other samples (at a 30% similarity score), indicating distinctly different viral populations. These samples include a mesotrophic sample that have previously been shown to have distinctly different viral populations than surface ocean samples [45]. Taken together, these data indicate that viral populations are structured globally by temperature, and at finer resolution by station (for surface and DCM samples) indicating that micronutrients and local conditions play an important role in defining viral populations.

Figure 5. Visualizing the genetic distance among marine viral communities using Libra.

Distance computed from 43 TOV from the 2009-2012 Tara Oceans Expedition. Lines (edges) between samples represent the similarity and are colored and thickened accordingly. Lines with insignificant similarity (less than 30%) are removed. Each of the sample names are color coded by Longhurst Province. Inner circles show temperature ranges. Sample names show the temperature range, station, and depth as indicated on the legend.

INNOVATIONS

Scientific collaboration is increasingly data driven given large-scale next generation sequencing datasets. It is now possible to generate, aggregate, archive, and share datasets that are terabytes and even petabytes in size. Scalability of a system is becoming a vital feature that decides feasibility of massive 'omic's analyses. In particular, this is important for metagenomics where patterns in global ecology can only be discerned by comparing the sequence signatures of microbial communities from massive 'omics datasets, given that most microbial genomes have not been defined. Current algorithms to perform these tasks run on local workstations or high-performance computing architectures that cannot scale. Libra presents three main innovations: the use of a scalable Apache Hadoop framework enabling massive dataset comparison, the use of sophisticated distance metrics allowing high accuracy and clustering of the metagenomes based on their k-mer content, and a web-based tool imbedded in the CyVerse advanced cyberinfrastructure through iMicrobe (http://imicrobe.us) for broader use of the tool in the scientific community. The work described here is the first step in implementing a cloud-based resource for comparative metagenomics that can be broadly used by scientists to analyze large-scale shared data resources. Moreover, the code can be ported to any MapReduce cluster (e.g., Wrangler at TACC, Amazon EMR or private Hadoop clusters). This computing paradigm is consistent with recent efforts to increase the accessibility of big datasets in the cloud, such as the Pan Cancer Analyses of Whole Genomes Project [46].

METHODS

Scalability benchmarking for Libra. We used synthetic datasets for a scalability benchmark. The synthesized datasets consisted of different number of samples, each of which is 10 billion bytes (approximately 9.3 GB). We took samples that are larger than 10 billion bytes from Tara ocean virome dataset and truncated each of them to approximately 10 billion bytes in size while respecting read boundaries. We varied the number of samples to show the scalability of Libra. We used four datasets consisting of 10, 20, 30 and 40 samples in the benchmark. Total sizes of the datasets are 93GB, 186GB, 279GB and 372GB respectively. Each experiment was run three times, and an average of the three runs reported (Supplemental Table 4).

Figure 6. Scalability testing for Libra. Four datasets consisting of 10, 20, 30 and 40 samples with total sizes of 93GB, 186GB, 279GB and 372GB, respectively. Runtime of Libra increased linearly with increased input volume and number of input samples. The linear increase of runtime shows that Libra efficiently handles increased volume of input and efficiently computes distances between all sample pairs while the number of sample pairs increases quadratically.

Benchmarking runtimes of different distance metrics in Libra. We used the same synthetic dataset with 40 samples (372GB in total) in the scalability benchmarking. We varied the distance metrics and measured the runtimes of Libra. Because all distance metrics share the same index, we reused the index constructed during the scalability benchmarking, thus, runtimes of the inverted index construction for the different metrics are the same. Each experiment was run three times, and an average of the three runs reported (Supplemental Table 4).

Figure 7. Runtimes of three different distance metrics (Cosine Similarity, Bray-Curtis and Jensen-Shannon) in Libra with 40 samples of input (372GB in total). Differences in runtimes are mainly due to different computational workload of distance metrics. For example, Jensen-

Shannon requires more multiplications and divisions in nested loops than cosine similarity, incurring more computational workload. Yet, distance matrix computation with Jensen-Shannon took only 12.64% of total runtime.

Experimental Environment Description:

Mash and SIMKA configurations. Mash v1.1 was run on the metagenomic datasets with the following parameters: -r –s 10000 –m 2 [19]. The analysis of assemblies was run without the parameter “-r”, used for short sequences.

SIMKA v1.3.2 was run on the metagenomic datasets with the following parameters: -abundance-min 2 -max-reads [MINCOUNT] -simple-dist -complex-dist, where [MINCOUNT] is the smallest sequence count across the analyzed samples.

Hadoop cluster configuration. The Libra experiments described in the paper were performed on a Hadoop cluster consisting of 10 physical nodes (9 MapReduce worker nodes). Each node contains 12 CPUs and 128 GB of RAM, and is configured to run a maximum of 7 YARN containers simultaneously with 10 GB of RAM per container. The remaining system resources are reserved for the operating system and other Hadoop services such as Hive or Hbase.

FUNDING

This work was supported by the National Science Foundation award #1640775 to BLH and JHH. System support and access for the Hadoop cluster was provided by University of Arizona Information Technology Services. System support and access for the Wrangler cluster was provided by Texas Advanced Computing Center. The following reagent was obtained through BEI Resources, NIAID, NIH as part of the Human Microbiome Project: Genomic DNA from Microbial Mock Community B (Staggered, High Concentration), v5.2H, for Whole Genome Shotgun Sequencing, HM-277D.

Competing interests: The authors declare no competing interests.

Availability and Implementation:

Project name: Libra
Project home page: http://github.com/iychoi/libra
Operating system(s): Hadoop 2.3 or higher
Programming language: Java
Other requirements: Java 1.7 or higher
License: Apache License Version 2.0
Any restrictions to use by non-academics: No restriction
Libra web-based App is in iMicrobe under Apps (http://imicrobe.us); Code to implement the Libra web-based App is in Github (https://github.com/hurwitzlab/occ-plan-b).

REFERENCES

1. Yooseph S, Sutton G, Rusch DB, Halpern AL, Williamson SJ, Remington K, et al. The Sorcerer II Global Ocean Sampling expedition: expanding the universe of protein families. PLoS Biol. 2007;5:e16.
2. Sunagawa S, Coelho LP, Chaffron S, Kultima JR, Labadie K, Salazar G, et al. Structure and function of the global ocean microbiome. Science. 2015;348.
3. Hurwitz BL, Sullivan MB. The Pacific Ocean Virome (POV): a marine viral metagenomic dataset and associated protein clusters for quantitative viral ecology. PLoS One. 2013;8:e57355.
4. Dubinkina VB, Ischenko DS, Ulyantsev VI, Tyakht AV, Alexeev DG. Assessment of k-mer spectrum applicability for metagenomic dissimilarity analysis. BMC Bioinformatics. 2016;17:38.
5. Teeling H, Waldmann J, Lombardot T, Bauer M, Glockner FO. TETRA: a web-service and a stand-alone program for the analysis and comparison of tetranucleotide usage patterns in DNA sequences. BMC Bioinformatics. 2004;5:163.
6. Wu Y-W, Ye Y. A novel abundance-based algorithm for binning metagenomic sequences using l-tuples. J Comput Biol. online.liebertpub.com; 2011;18:523–34.
7. Fofanov Y, Luo Y, Katili C, Wang J, Belosludtsev Y, Powdrill T, et al. How independent are the appearances of n-mers in different genomes? Bioinformatics. 2004;20:2421–8.
8. Maillet N, Lemaitre C, Chikhi R, Lavenier D, Peterlongo P. Compareads: comparing huge metagenomic experiments. BMC Bioinformatics. 2012;13 Suppl 19:S10.
9. Maillet N, Collet G, Vannier T, Lavenier D, Peterlongo P. Commet: Comparing and combining multiple metagenomic datasets. 2014 IEEE International Conference on Bioinformatics and Biomedicine (BIBM). 2014. p. 94–8.
10. Ulyantsev VI, Kazakov SV, Dubinkina VB, Tyakht AV, Alexeev DG. MetaFast: fast reference-free graph-based comparison of shotgun metagenomic data. Bioinformatics. 2016;32:2760–7.
11. Ondov BD, Treangen TJ, Melsted P, Mallonee AB, Bergman NH, Koren S, et al. Mash: fast genome and metagenome distance estimation using MinHash. Genome Biol. 2016;17:132.
12. Chum O, Philbin J, Zisserman A. Near Duplicate Image Detection: min-Hash and tf-idf Weighting. BMVC. 2008; Available from: http://www.bmva.org/bmvc/2008/papers/119.pdf
13. Benoit G, Peterlongo P, Mariadassou M, Drezen E, Schbath S, Lavenier D, et al. Multiple comparative metagenomics using multiset k-mer counting. PeerJ Comput Sci. 2016;2:e94.
14. Zaharia M, Chowdhury M, Franklin MJ, Shenker S, Stoica I. Spark: Cluster computing with working sets. HotCloud. static.usenix.org; 2010;10:95.
15. Guo R, Zhao Y, Zou Q, Fang X, Peng S. Bioinformatics applications on Apache Spark. Gigascience. 2018.

16. Kolker N, Higdon R, Broomall W, Stanberry L, Welch D, Lu W, et al. Classifying proteins into functional groups based on all-versus-all BLAST of 10 million proteins. OMICS. online.liebertpub.com; 2011;15:513–21.
17. McKenna A, Hanna M, Banks E, Sivachenko A, Cibulskis K, Kernytsky A, et al. The Genome Analysis Toolkit: a MapReduce framework for analyzing next-generation DNA sequencing data. Genome Res. genome.cshlp.org; 2010;20:1297–303.
18. Langmead B, Schatz MC, Lin J, Pop M, Salzberg SL. Searching for SNPs with cloud computing. Genome Biol. 2009;10:R134.
19. Nguyen T, Shi W, Ruden D. CloudAligner: A fast and full-featured MapReduce based tool for sequence mapping. BMC Res Notes. 2011;4:171.
20. Schatz MC. BlastReduce: high performance short read mapping with MapReduce. University of Maryland, Available from: https://www.cs.umd.edu/sites/default/files/scholarly_papers/MichaelSchatz_1.pdf
21. Schatz MC. CloudBurst: highly sensitive read mapping with MapReduce. Bioinformatics. 2009;25:1363–9.
22. Pandey RV, Schlötterer C. DistMap: a toolkit for distributed short read mapping on a Hadoop cluster. PLoS One. 2013;8:e72614.
23. Nordberg H, Bhatia K, Wang K, Wang Z. BioPig: a Hadoop-based analytic toolkit for large-scale sequence data. Bioinformatics. 2013;29:3014–9.
24. Gao T, Guo Y, Wei Y, Wang B, Lu Y, Cicotti P, et al. Bloomfish: A Highly Scalable Distributed K-mer Counting Framework. 2017 IEEE 23rd International Conference on Parallel and Distributed Systems (ICPADS). 2017. p. 170–9.
25. Menon RK, Bhat GP, Schatz MC. Rapid Parallel Genome Indexing with MapReduce. Proceedings of the Second International Workshop on MapReduce and Its Applications. New York, NY, USA: ACM; 2011. p. 51–8.
26. Salton G, Wong A, Yang CS. A Vector Space Model for Automatic Indexing. Commun ACM. New York, NY, USA: ACM; 1975;18:613–20.
27. Brum JR, Ignacio-Espinoza JC, Roux S, Doulcier G, Acinas SG, Alberti A, et al. Patterns and ecological drivers of ocean viral communities. Science. 2015;348.
28. Goff SA, Vaughn M, McKay S, Lyons E, Stapleton AE, Gessler D, et al. The iPlant Collaborative: Cyberinfrastructure for Plant Biology. Front Plant Sci. 2011;2:34.
29. Devisetty UK, Kennedy K, Sarando P, Merchant N, Lyons E. Bringing your tools to CyVerse Discovery Environment using Docker. F1000Res. 2016;5:1442.
30. McElroy KE, Luciani F, Thomas T. GemSIM: general, error-model based simulator of next-generation sequencing data. BMC Genomics. 2012;13:74.
31. Human Microbiome Project Consortium. Structure, function and diversity of the healthy human microbiome. Nature. 2012;486:207–14.
32. Diepenbroek M, Grobe H, Reinke M, Schindler U, Schlitzer R, Sieger R, et al. PANGAEA—an information system for environmental sciences. Comput Geosci. 2002;28:1201–10.
33. O'Malley O. Terabyte sort on apache hadoop. Yahoo, available online at: http://sortbenchmark org/Yahoo-Hadoop pdf,(May). Citeseer; 2008;1–3.
34. Huang A. Similarity measures for text document clustering. Proceedings of the sixth new zealand computer science research student conference (NZCSRSC2008), Christchurch, New Zealand. 2008. p. 49–56.
35. Michie MG. Use of the Bray-Curtis similarity measure in cluster analysis of foraminiferal data. Math Geol. 1982;14:661–7.
36. Lin J. Divergence measures based on the Shannon entropy. IEEE Trans Inf Theory. 1991;37:145–51.
37. Watts GS, Youens-Clark K, Slepian MJ, Wolk DM, Oshiro MM, Metzger GS, et al. 16S rRNA gene sequencing on a benchtop sequencer: accuracy for identification of clinically important bacteria. J Appl Microbiol. 2017;123:1584–96.

38. Bergh O, Borsheim KY, Bratbak G, Heldal M. High abundance of viruses found in aquatic environments. Nature. 1989;340:467–8.
39. Hurwitz BL, Brum JR, Sullivan MB. Depth-stratified functional and taxonomic niche specialization in the “core”and “flexible”Pacific Ocean Virome. ISME J. 2014.
40. Minot S, Wu GD, Lewis JD, Bushman FD. Conservation of gene cassettes among diverse viruses of the human gut. PLoS One. 2012;7:e42342.
41. Sun Y, Cai Y, Huse SM, Knight R, Farmerie WG, Wang X, et al. A large-scale benchmark study of existing algorithms for taxonomy-independent microbial community analysis. Brief Bioinformatics. 2012;13:107–21.
42. Edgar RC. Search and clustering orders of magnitude faster than BLAST. Bioinformatics. 2010;26:2460–1.
43. Niu BF, Fu LM, Sun SL, Li WZ. Artificial and natural duplicates in pyrosequencing reads of metagenomic data. BMC Bioinformatics. 2010;11:187.
44. Cai Y, Sun Y. ESPRIT-Tree: hierarchical clustering analysis of millions of 16S rRNA pyrosequences in quasilinear computational time. Nucleic Acids Res. 2011;39:e95.
45. Hurwitz BL, Brum JR, Sullivan MB. Depth-stratified functional and taxonomic niche specialization in the “core” and “flexible” Pacific Ocean Virome. ISME J. 2015;9:472–84.
46. Weinstein JN, Collisson EA, Mills GB, Shaw KRM, Ozenberger BA, Ellrott K, et al. The cancer genome atlas pan-cancer analysis project. Nat Genet. 2013;45:1113–20.
47. Wrangler - Texas Advanced Computing Center. [cited 2017 Dec 20]. Available from: https://www.tacc.utexas.edu/systems/wrangler

Figure 1

Click here to download Figure Figure 1.pdf

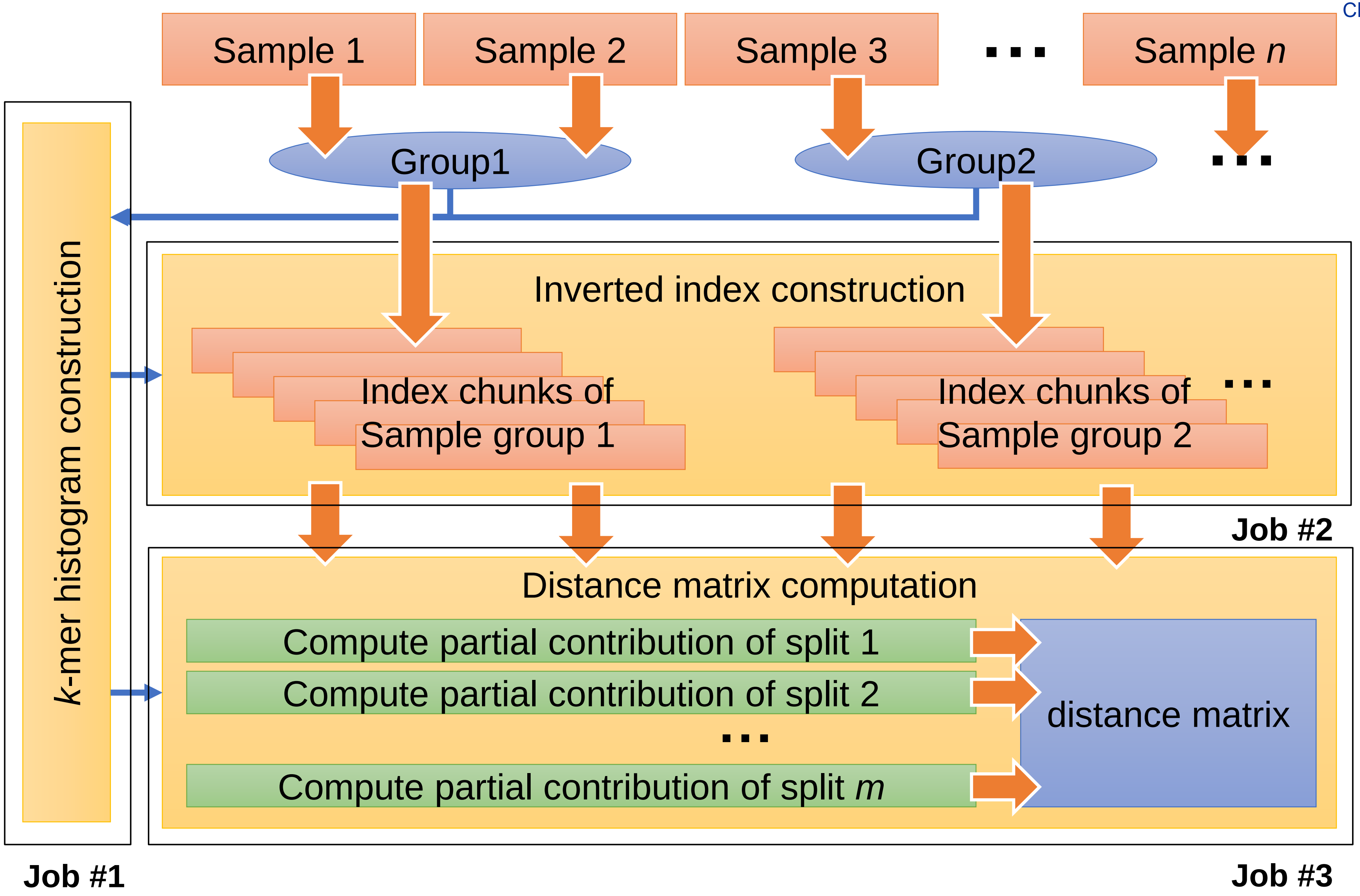

Figure 2

Click here to download Figure Figure 2.pdf

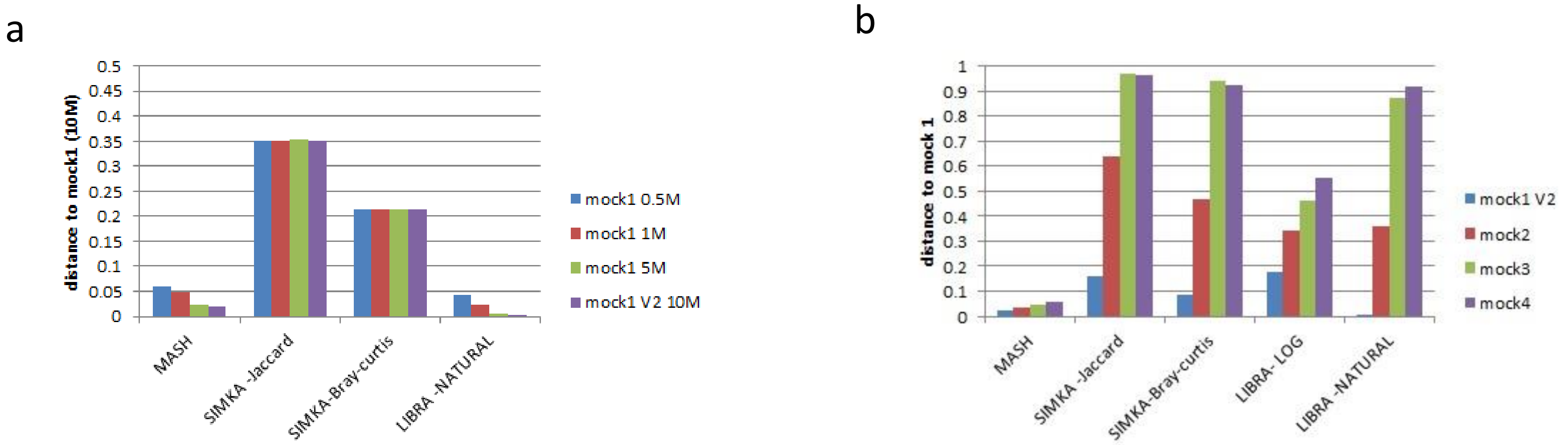

Figure 3

Click here to download Figure Figure 3.pdf

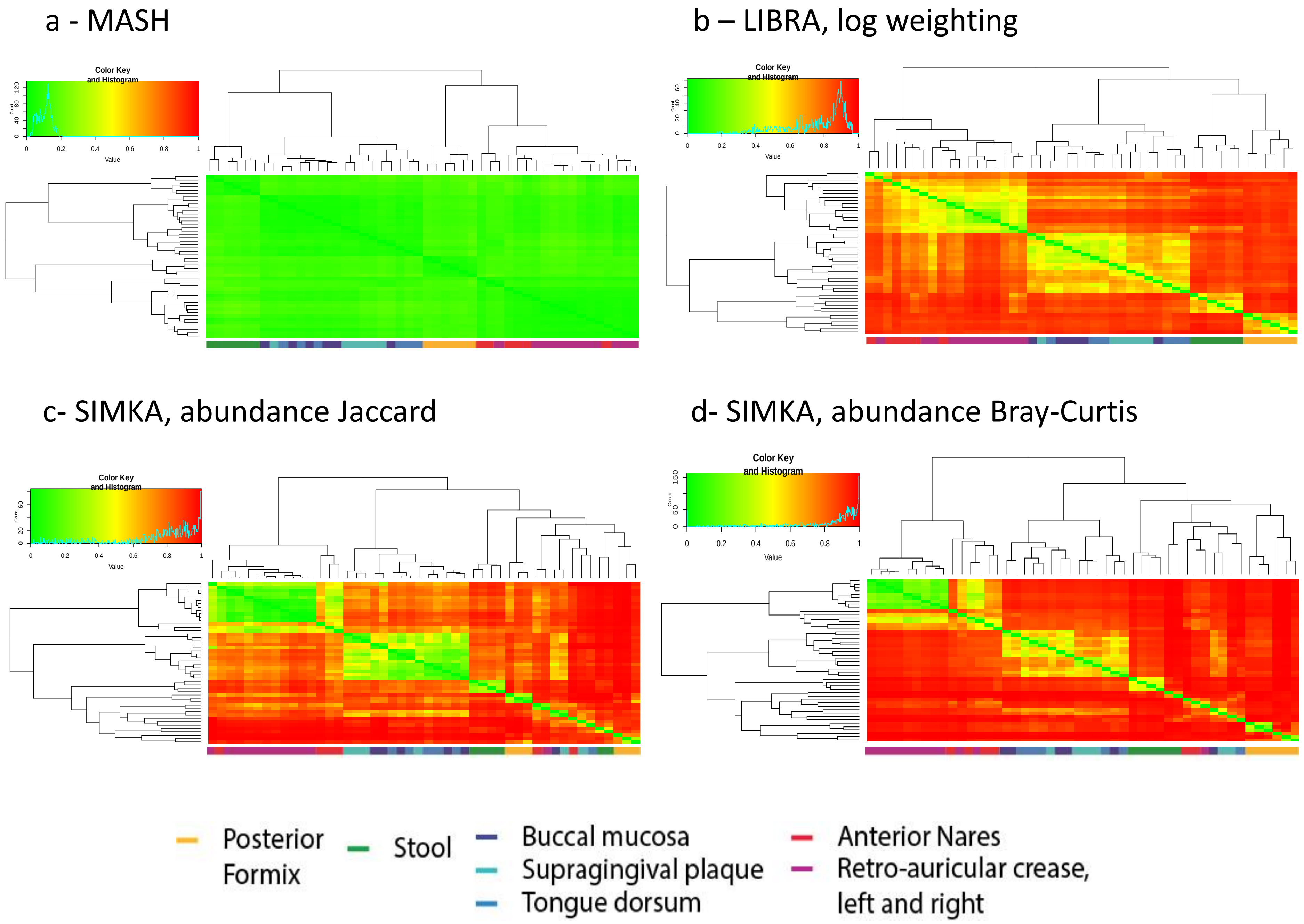

Figure 4

Click here to download Figure Figure 4.pdf

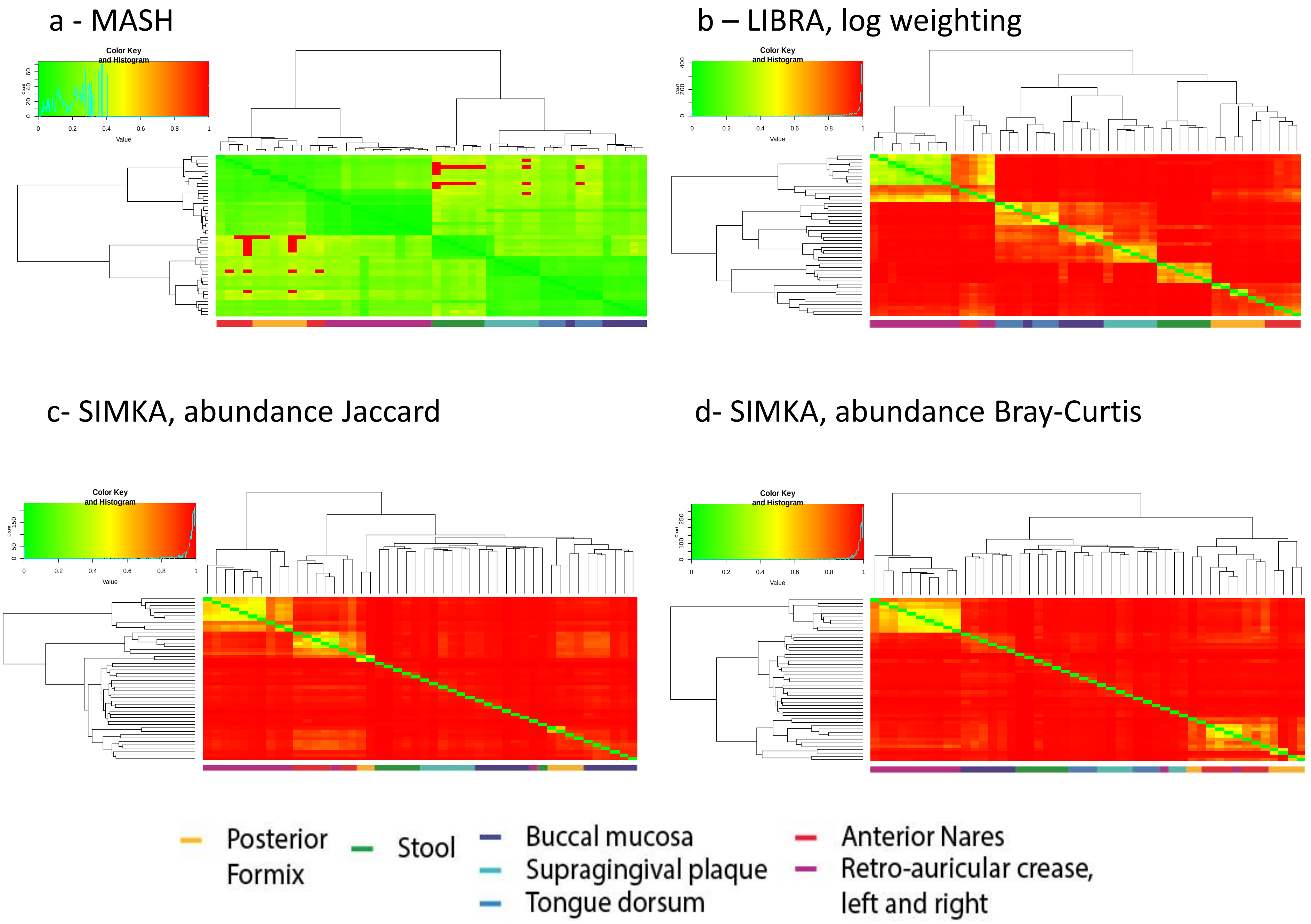

Figure 5

Click here to download Figure Figure 5.pdf

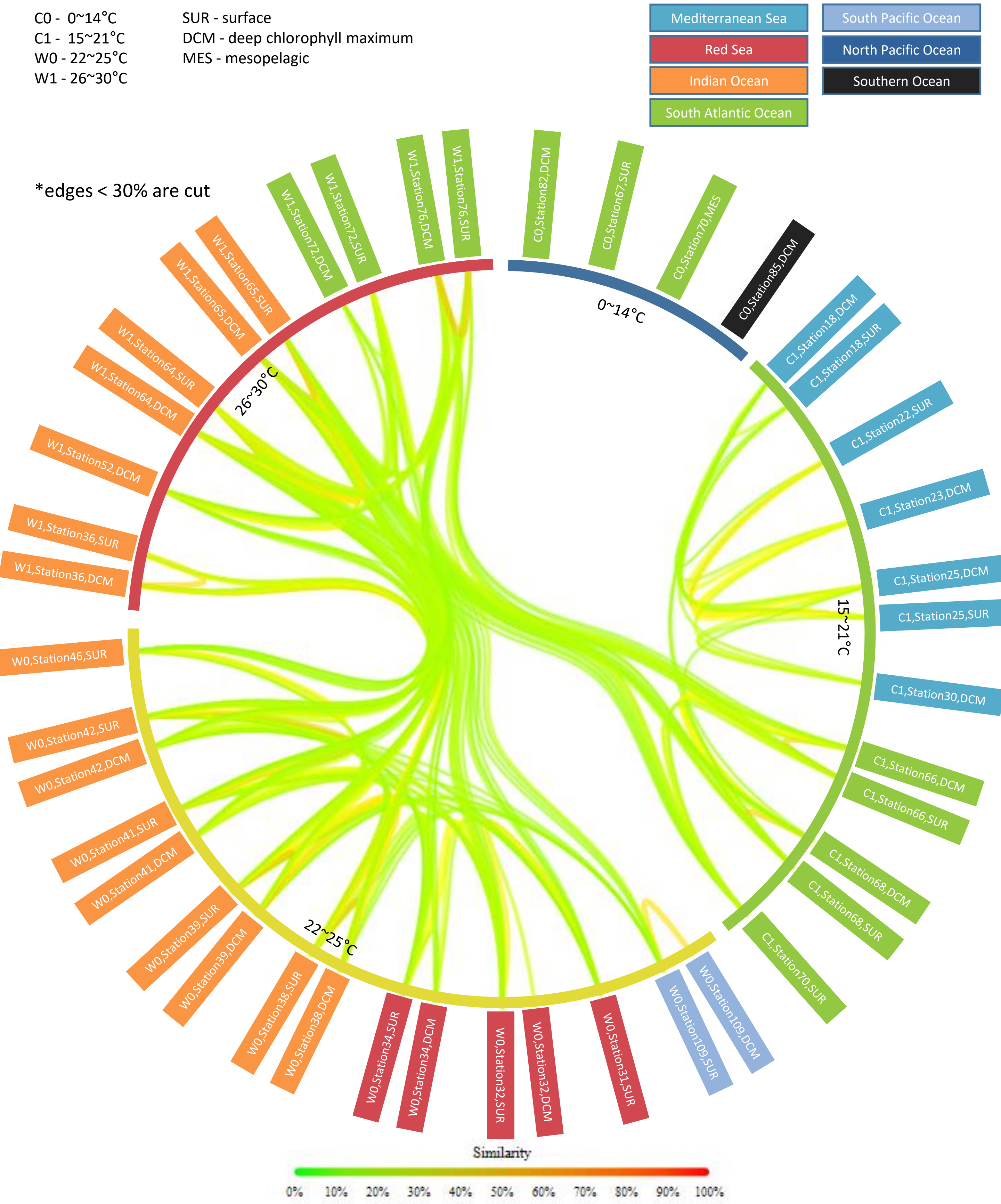

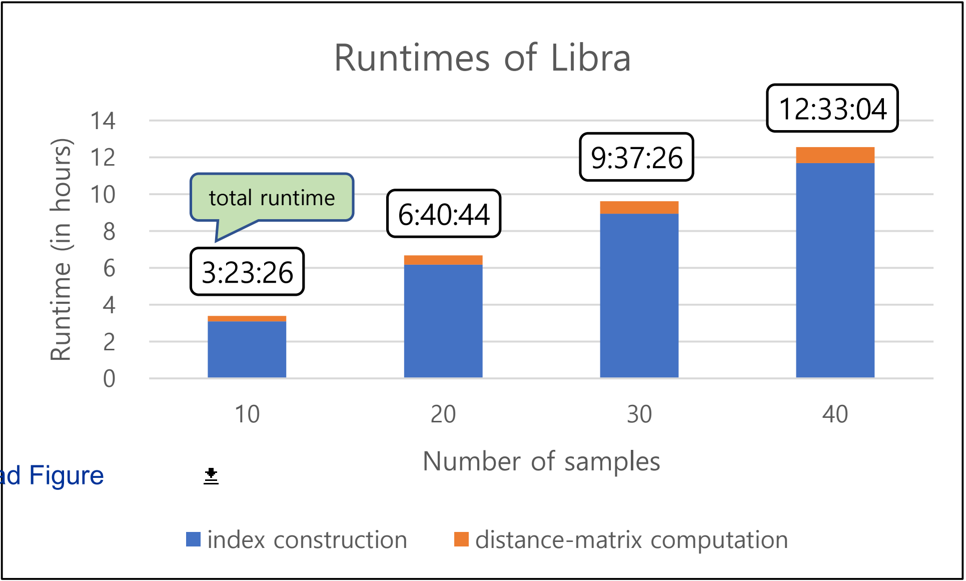
Runtimes of Libra
Runtime (in hours)
0
2
4
6
8
10
12
14
total runtime
3:23:26
6:40:44
9:37:26
12:33:04
10
20
30
40
Number of samples
index construction
distance-matrix computation

ad Figure

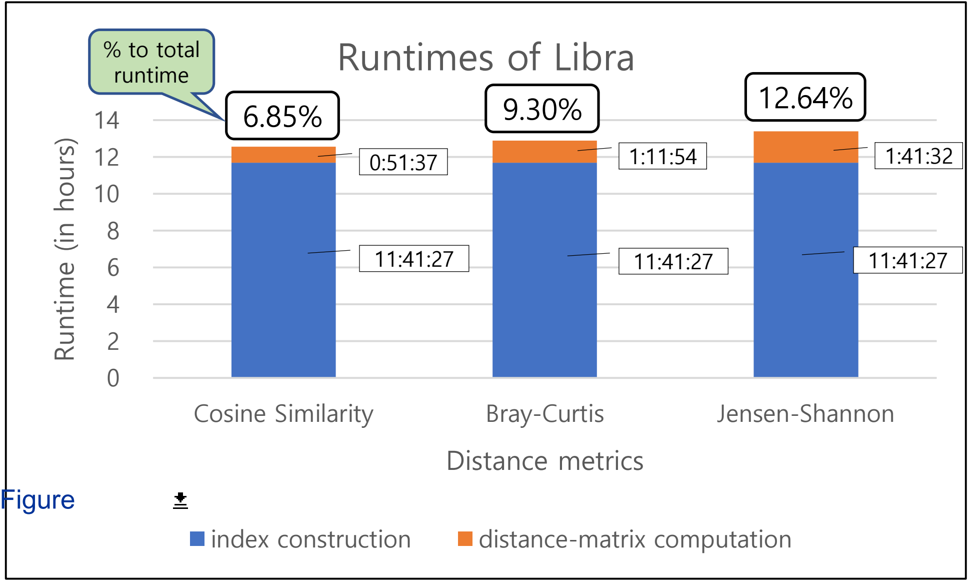
Runtimes of Libra
% to total runtime
6.85%
9.30%
12.64%
0:51:37
1:11:54
1:41:32
11:41:27
11:41:27
11:41:27
Runtime (in hours)
14
12
10
8
6
4
2
0
Cosine Similarity
Bray-Curtis
Jensen-Shannon
Distance metrics
index construction
distance-matrix computation
Figure

Click here to access/download
Supplementary Material
Supplemental_methods_and_fig_table_legends.docx

Supplementary Figure 1

Click here to access/download
Supplementary Material
supplemental Figure 1.pdf

Supplementary Figure 2

Click here to access/download
Supplementary Material
Supplemental Figure 2.pdf

Supplementary Figure 3

Click here to access/download
Supplementary Material
Supplemental Figure 3.pdf

Supplementary Table 1

Click here to access/download
Supplementary Material
Supplemental Table 1.xlsx

Supplementary Table 2

Click here to access/download
Supplementary Material
Supplemental Table 2.xlsx

Supplementary Table 3

Click here to access/download
Supplementary Material
Supplemental Table 3.xlsx

Supplementary Table 4

Click here to access/download
Supplementary Material
Supplemental Table 4.xlsx

Personal Cover

Click here to download Personal Cover Gigascience_Cover_Letter.docx

THE UNIVERSITY OF ARIZONA

College of Agriculture and Life Sciences

Department of Agricultural and Biosystems

Shantz Bldg., B38, Room 403
1177 E. 4th Street
P.O. Box 210038
Tucson, AZ 85721-0038
Tel: (520) 621-1607
Fax: (520) 621-3963

August 24, 2018

Dear Editors,

Please find our paper for consideration at *Gigascience* as a research article titled "Libra: robust biological inferences of global datasets using scalable k-mer based all-vs-all metagenomic comparisons".

Microbiome research spans a broad array of disciplines from medicine, agriculture, bioenergy, and the environment, and is united in addressing core scientific questions relating microbial communities to biological and chemical processes in human, animal, or Earth systems. Given the preponderance of genomic data from diverse environments, there is a new desire to ask cross-cutting questions from the environment to human health. To move this work forward, microbiome datasets need to be holistically analyzed to examine how microbes move through living systems. Currently, only a subset of tools are available that make these analyses possible (through data reduction techniques and read count normalization), but none exploit big data architectures to scale compute and analyze complete datasets (100% of reads) in a linear and fault tolerant manner. This level of resolution is vital in metagenomic analyses where > 50% of the reads are unknown and the only way to understand functional changes in microbial communities is through all-vs-all analysis of diverse datasets to associate sequence patterns with environmental factors. To date, no tool offers a scalable and complete analysis of reads to explore global patterns in microbiome sciences.

Here we describe the first scalable algorithm for comparative metagenomics called Libra that is capable of performing an all-vs-all sequence analysis on hundreds of metagenomes in a Hadoop big data framework. Libra performs with unparalleled accuracy compared to equivalent tools using both simulated and real metagenomic datasets ranging from 80 million to 4.2 billion reads. In contrast to current methods, Libra's state-of-the-art algorithm and its implementation in a big data architecture does not require a reduction in dataset size or simplified distance metrics to achieve remarkable compute times and accuracy. As a result, Libra enables integration of massive datasets across disciplines to identify microbial and viral signatures linked to key biological processes. Moreover, Libra is available as an open-access web-based tool in iMicrobe (http://imicrobe.us) and in Github where the code is available for further optimization and reuse by the community. All authors declare no competing interests and have approved the manuscript for submission. The content of the manuscript has not been published, or submitted for publication elsewhere. Thank you for considering our paper for publication in *Gigascience*.

Sincerely,

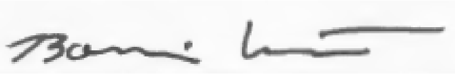

Bonnie Hurwitz, PhD
Assistant Professor of Biosystems Engineering
University of Arizona, bhurwitz@email.arizona.edu
