# Supplementary material for: Libra: scalable k-mer–based tool for massive all-vs-all metagenome comparisons
Source: Gigascience. 2018 Dec 28;8(2):giy165. doi: 10.1093/gigascience/giy165 (PMC6354030; doi:10.1093/gigascience/giy165)
Supplement: Supplemental Files [file giy165_supplemental_files.zip › Gigascience_Libra_Manuscript_TrackedChanges.docx]

**Title:** Libra: scalable k-mer based tool for massive all-vs-all metagenome comparisons.

**Authors:** Illyoung Choi^1^, Alise J. Ponsero^2^, Matthew Bomhoff^2^, Ken Youens-Clark^2^, John H. Hartman^1*^, and Bonnie L. Hurwitz^2,3*^

**Affiliations:**

^1^Department of Computer Science, University of Arizona, Tucson, Arizona

^2^Department of Biosystems Engineering, University of Arizona, Tucson, Arizona

^3^BIO5 Institute, University of Arizona, Tucson, Arizona

**Corresponding Author:**

Bonnie L. Hurwitz [bhurwitz@email.arizona.edu](mailto:bhurwitz@email.arizona.edu)

**ABSTRACT**

**Background**: Shotgun metagenomics provides powerful insights into microbial community biodiversity and function. Yet, inferences from metagenomic studies are often limited by dataset size and complexity, and are restricted by the availability and completeness of existing databases. *De novo* comparative metagenomics enables the comparison of metagenomes based on their total genetic content.

**Results**: We developed a tool called Libra that performs all-vs-all comparison of metagenomes for precise clustering based on their k-mer content. This tool presents three main innovations: the use of a scalable Apache Hadoop framework to enables massive metagenome comparisons, cosine similarity for calculating the distance using sequence composition and abundance while normalizing for sequencing depth, and a web-based tool in iMicrobe (<http://imicrobe.us>) that uses the CyVerse advanced cyberinfrastructure to promote broad use of the tool by the scientific community.

**Conclusions**: A comparison of Libra to equivalent tools using both simulated and real metagenomic datasets, ranging from 80 million to 4.2 billion reads, reveals that methods commonly implemented to reduce compute time for large datasets—such as data reduction, read count normalization, and presence/absence distance metrics—greatly diminish the resolution of large-scale comparative analyses. In contrast, Libra uses all of the reads to calculate k-mer abundance in a Hadoop architecture that can scale to any size dataset to enable global-scale analyses and link microbial signatures to biological processes.

**Keywords**: metagenomics, Hadoop, k-mer, distance metrics, clustering

**INTRODUCTION**

Over the last decade, scientists have generated petabytes of genomic data to uncover the role of microbes in dynamic living systems. Yet to understand the underlying biological principles that guide the distribution of microbial communities, massive ‘omics datasets need to be compared with environmental factors to find linkages across space and time. One of the greatest challenges in these endeavors has been in documenting and analyzing unexplored genetic diversity in wild microbial communities. For example, fewer than 60% of 40 million non-redundant genes from the Global Ocean Survey (GOS) and the Tara Oceans Expeditions match known proteins in bacteria [[1,2]](https://paperpile.com/c/8kIEbl/k1BC4+96Fzn). Other microorganisms such as viruses or pico- eukaryotes that are important to ocean ecosystems are even less well defined (e.g. < 7% of reads from viromes match known proteins [[3]](https://paperpile.com/c/8kIEbl/6r8Pm)). This is largely due to the fact that reference genomes for these organisms do not exist in public data repositories and genome-sequences from metagenomic data await better taxonomic and functional definition. As a result, even advanced tools such as k-mer based classifiers that rapidly assign metagenomic reads to known microbes (Table 1) miss “microbial dark matter” that comprises a significant proportion of metagenomes [(Morgan and Huttenhower 2012)](https://paperpile.com/c/8kIEbl/S8WK)

|  |  |  |  |  |  |  |  |  |  |  |
| --- | --- | --- | --- | --- | --- | --- | --- | --- | --- | --- |
|  |  |  |  |  |  |  |  |  |  |  |
|  |  |  |  |  |  |  |  |  |  | |
|  |  |  |  |  |  |  |  |  |  |  |
|  |  |  |  |  |  |  |  |  |  |  |
|  |  |  |  |  |  |  |  |  |  |  |
|  |  |  |  |  |  |  |  |  |  |  |
|  |  |  |  |  |  |  |  |  |  |  |
|  |  |  |  |  |  |  |  |  |  |  |
|  |  |  |  |  |  |  |  |  |  |  |
|  |  |  |  |  |  |  |  |  |  |  |
|  |  |  |  |  |  |  |  |  |  |  |
|  |  |  |  |  |  |  |  |  |  |  |
|  |  |  |  |  |  |  |  |  |  |  |
|  |  |  |  |  |  |  |  |  |  |  |
|  |  |  |  |  |  |  |  |  |  |  |
|  |  |  |  |  |  |  |  |  |  |  |
|  |  |  |  |  |  |  |  |  |  |  |
|  |  |  |  |  |  |  |  |  |  |  |
|  |  |  |  |  |  |  |  |  |  |  |
|  |  |  |  |  |  |  |  |  |  |  |
|  |  |  |  |  |  |  |  |  |  |  |
|  |  |  |  |  |  |  |  |  |  |  |
|  | | | | | | | |  |  |  |
|  | | | | | | | |  |  |  |
|  | | | | | | | | | | |

***De novo* comparative metagenomics offers a path forward.** In order to examine the complete genomic content, metagenomic samples can be compared using their sequence signature (or frequency of k-mers; Table 1). This approach relies on three core tenets of k-mer-based analytics: (i) closely related organisms share k-mer profiles and cluster together, making taxonomic assignment unnecessary [[4,5]](https://paperpile.com/c/8kIEbl/ZdF41+heFng), (ii) k-mer frequency is correlated with the abundance of an organism [[6]](https://paperpile.com/c/8kIEbl/Z5qS4), and (iii) k-mers of sufficient length can be used to distinguish specific organisms [[7]](https://paperpile.com/c/8kIEbl/bqvNX). In 2012, the Compareads [[8]](https://paperpile.com/c/8kIEbl/BqLrD) method was proposed, followed by Commet [[9]](https://paperpile.com/c/8kIEbl/vVxsY). Both of these tools compute the number of shared reads between metagenomes using a k-mer-based read similarity measure. The number of shared reads between datasets is then used to compute a Jaccard distance between samples. Given the computational intensity of all-vs-all sequence analysis, several other methods have been employed to reduce the dimensionality of metagenomes and speed up analyses by creating unique k-mer sets and computing the genetic distance between pairs of metagenomes, such as MetaFast [[10]](https://paperpile.com/c/8kIEbl/M6kKE) and Mash [[11]](https://paperpile.com/c/8kIEbl/RWhtK). The fastest of these methods, Mash [(Benoit et al. 2016)](https://paperpile.com/c/8kIEbl/LTd9P), indexes samples by unique k-mers to create size-reduced sketches, and compares these sketches using the MinHash algorithm [[12]](https://paperpile.com/c/8kIEbl/9Emr) for computing a genetic distance using Jaccard similarity. Yet, the tradeoff for speed is that samples are reduced to a subset of unique k-mers (1k by default) that may lead inaccurate k-mer profile of the samples. Further, given that Mash uses Jaccard similarity only the genetic distance between samples is accounted for (or genetic content in microbial communities) without considering abundance (dominant vs rare organisms in the sample) which is central to microbial ecology and ecosystem processes [(Koslicki and Zabeti 2017)](https://paperpile.com/c/8kIEbl/Vgyg).

Recently, SIMKA [[13]](https://paperpile.com/c/8kIEbl/LTd9P) was developed to compute a distance matrix between metagenomes by dividing the input datasets into abundance vectors from subsets of k-mers, then rejoining the resulting abundances in a cumulative distance matrix. The methodology can be parallelized to execute the analyses on a high-performance compute cluster (HPC). SIMKA also provides various ecological distance metrics to let the user choose the metric most relevant to their analysis. However, the computational time varies based on the distance metric, where some distances scale linearly and other distances metrics, like Jensen-Shannon scale quadratically as additional samples are added [[13]](https://paperpile.com/c/8kIEbl/LTd9P). Moreover, SIMKA normalizes datasets in an all-vs-all comparison by reducing the depth of sequencing for all samples to the least common denominator, therefore decreasing the resolution of the datasets. Lastly, computing k-mer analytics using HPC is subject to reduced fault tolerance for massive datasets.

**Scaling sequence analysis using big data analytics via Hadoop.** Hadoop is an attractive platform for performing large-scale sequence analysis because it provides a distributed file system and distributed computation for analyzing massive amounts of data. Hadoop clusters are comprised of commodity servers so that the processing power increases as more computing resources are added. Hadoop also offers a high-level programming abstraction, called MapReduce [[14]](https://paperpile.com/c/8kIEbl/jTfy) that greatly simplifies the implementation of new analytical tools. Programmers do not need specialized training in distributed systems and networking to implement distributed programs using MapReduce. Hadoop also provides fault-tolerance by default. When a Hadoop node fails, Hadoop reassigns the failed node’s tasks to another node containing a redundant copy of the data those jobs were processing. This differs from HPC where schedulers track failed nodes and either restart the failed computation from the most recent checkpoint, or from the beginning if checkpointing wasn’t used. Thus, using a Hadoop infrastructure ensures that computations and data are protected even in the event of hardware failures. These benefits have led to new analytic tools based on Hadoop, making Hadoop a de facto standard in large-scale data analysis. In metagenomics, the development of efficient and inexpensive high-throughput sequencing technologies has lead to a rapid increase of the amount of sequence data for studying microbes in diverse environments. However, no Hadoop-enabled comparative metagenomics tools currently exist.

Spark [[15]](https://paperpile.com/c/8kIEbl/tdI8) is increasingly popular for scientific data analysis [[16]](https://paperpile.com/c/8kIEbl/GFlP) because of its outstanding performance provided by fast in-memory processing. Although Libra is currently implemented on Hadoop MapReduce, Libra can be easily ported to Spark because both Hadoop MapReduce and Spark have similar interfaces for data processing and partitioning. For example, Resilient Distributed Datasets (RDD) can be partitioned and distributed over a Spark cluster using Libra’s k-mer range partitioning. RDDs are memory-resident, allowing Spark to significantly improve the performance of Libra’s k-mer counting and distance matrix computation by avoiding slow disk I/O for intermediate data. Nevertheless, we implemented Libra using Hadoop MapReduce because Spark requires much more RAM than Hadoop MapReduce, significantly increasing the cost of the cluster.

**Existing big data algorithms compare reads to limited genomic reference data**. Recent progress has been made in translating bioinformatics algorithms to big data architectures to overcome scalability issues (Table 1). Thus far, these algorithms compare large-scale NGS datasets to reference genomic datasets and replace computationally intensive algorithms such as sequence alignment [[17]](https://paperpile.com/c/8kIEbl/A552F), genetic variant detection [[18,19]](https://paperpile.com/c/8kIEbl/5S5KM+NqwE2), or short read mapping [[20–23]](https://paperpile.com/c/8kIEbl/Ft9hw+sDd86+mdmoh+gl8cP). For example, BlastReduce and CloudBurst are parallel sequence mapping tools based on Hadoop MapReduce [[21,22]](https://paperpile.com/c/8kIEbl/sDd86+mdmoh). These tools, however, implement a query-to-a-reference approach that is inefficient for all-vs-all analyses of reads from metagenomes. Other algorithms such as BioPig [[24]](https://paperpile.com/c/8kIEbl/nqMT) and Bloomfish [[25]](https://paperpile.com/c/8kIEbl/07Nl) generate an index of sequence data for later partial sequence search and k-mer counting using Hadoop [[26]](https://paperpile.com/c/8kIEbl/1e0Mk). These tools, however, adopt a suffix array approach similar to traditional bioinformatics tools that is inefficient in reading and indexing data on a distributed file system such as HDFS, a file system of Hadoop, thus reducing performance. Moreover, neither tool offers an end-to-end solution for comparing metagenomes consisting of: data distribution on a Hadoop cluster, k-mer indexing and counting, distance matrix computation, and visualization. Finally none of these tools are enabled in an advanced cyberinfrastructure where users can compute analyses in a simple web-based platform.

**Libra: a tool for scalable all-vs-all sequence analysis in an advanced cyberinfrastructure**

Here, we describe a scalable algorithm called Libra that is capable of performing all-vs-all sequence analysis using Hadoop MapReduce (SciCrunch.org tool reference ID SCR_016608). We demonstrate for the first time that Hadoop MapReduce can be applied to all-vs-all sequence comparisons of large-scale metagenomic datasets comprised of mixed microbial communities. We demonstrate that Cosine Similarity [[27]](https://paperpile.com/c/8kIEbl/6poA), which is widely used in document clustering and information retrieval, can be a good distance metric for comparing datasets to consider genetic distance and microbial abundance simultaneously, along with widely accepted distance metrics in biology such as Bray-Curtis [[28]](https://paperpile.com/c/8kIEbl/RAEY) and Jensen-Shannon [[29]](https://paperpile.com/c/8kIEbl/Pllm). We validate this distance metric using simulated metagenomes to show that Libra has exceptional sensitivity in distinguishing complex mixed microbiomes. Next, we show Libra’s ability to distinguish metagenomes by both community composition and abundance using 48 samples (16S rRNA and WGS) from the human microbiome project (HMP) across diverse body sites, and compare the results to Mash and SIMKA. Finally, we show that Libra can scale to massive global-scale datasets by examining viral diversity in 43 Tara Ocean Viromes (TOV) from the 2009-2011 Expedition [[30]](https://paperpile.com/c/8kIEbl/rCFoW) that represent 26 sites containing about 4.2 billion reads. The resulting data demonstrate that Libra provides accurate, efficient, and scalable compute for comparative metagenomics that can be used to discern global patterns in microbial ecology.

To promote the broad use of the Libra algorithm we developed a web-based tool in iMicrobe (<http://imicrobe.us>), where users can run Libra using data in their free CyVerse [[31,32]](https://paperpile.com/c/8kIEbl/3YGkv+luhg4) account or use datasets that are integrated into the iMicrobe Data Commons. These analyses are fundamental for determining relationships among diverse metagenomes to inform follow-up analyses on microbial-driven biological processes.

**DATA DESCRIPTION**

**Staggered mock community.** We performed metagenomic shotgun sequencing on a staggered mock community obtained from the Human Microbiome Consortium (HM-277D). The staggered mock community is comprised of genomic DNA from genera commonly found on or within the human body, consisting of 1,000 to 1,000,000,000 16S rRNA gene copies per organism per aliquot. The resulting DNA was subjected to whole genome sequencing as follows. Mixtures were diluted to a final concentration of 1 nanogram/microliter and used to generate whole genome sequencing libraries with the Ion Xpress Plug Fragment Library Kit and manual #MAN0009847, revC (Thermo Fisher Scientific, Waltham, MA, USA). Briefly, 10 nanograms of bacterial DNA was sheared using the Ion Shear enzymatic reaction for 12 min and Ion Xpress barcode adapters ligated following end repair. Following barcode ligation, libraries were amplified using the manufacturer’s supplied Library Amplification primers and recommended conditions. Amplified libraries were size selected to ~ 200 base pairs using the Invitrogen E-gel Size Select Agarose cassettes as outlined in the Ion Xpress manual and quantitated with the Ion Universal Library quantitation kit. Equimolar amounts of the library were added to an Ion PI Template OT2 200 kit V3. The resulting templated beads were enriched with the Ion OneTouch ES system and quantitated with the Qubit Ion Sphere Quality Control kit (Life Technologies) on a Qubit 3.0 fluorometer (Qubit, NY, NY, USA). Enriched templated beads were loaded onto an Ion PI V2 chip and sequenced according to the manufacturer’s protocol using the Ion PI Sequencing 200 kit V3 on a Ion Torrent Proton sequencer. The sequence data comprised of ~80 million reads have been deposited to the NCBI Sequence Read Archive under accession SRP115095 under project accession PRJNA397434.

**Simulated data derived from the staggered mock community***.* The resulting sequence data from the staggered mock community (~80 million reads) were used to develop simulated metagenomes to test the effects of varying read depth, and composition and abundance of organisms in mixed metagenomes. To examine read depth (in terms of raw read counts and file size), we used the known staggered mock community abundance profile to generate a simulated metagenome using GemSim [[33]](https://paperpile.com/c/8kIEbl/ftnTG) of 2 million reads (454 sequencing) and duplicated the dataset 2x, 5x and 10x. We also simulated the effects of sequencing a metagenome more deeply using GemSim [[33]](https://paperpile.com/c/8kIEbl/ftnTG) to generate simulated metagenomes with 0.5, 1, 5, and 10 million reads based on the relative abundance of organisms in the staggered mock community. Next, we developed four simulated metagenomes to test the effect of changing the dominant organism abundance and genetic composition including: 10 million reads from the staggered mock community (mock 1), the mock community with alterations in a few abundant species (mock 2), the mock community with many alterations in abundant species (mock 3), and mock 3 with additional sequences from archaea to further alter the genetic composition (mock 4) as described in Supplemental Table 1. The same community profiles were used to generate paired-end illumina dataset (100 million reads), using GemSim (illumina v4 error model). Finally, using SimLord, the community profiles were used to generate simulated third generation sequencing datasets (Pacific Bioscience SMRT sequencing - 1 million reads). SimLord default parameters were used to generate those simulated datasets.

All simulated datasets are available in iMicrobe (<http://imicrobe.us>).

**Human microbiome 16S rRNA gene amplicons and WGS reads**. Human microbiome datasets were downloaded from the NIH Human microbiome project [[34]](https://paperpile.com/c/8kIEbl/Z7p6a) including 48 samples from 5 body sites including: urogenital (posterior fomix), gastrointestinal (stool), oral (buccal mucosa, supragingival plaque, tongue dorsum), airways (anterior nares), and skin (retroauricular crease left and right) ([See Supplemental Table 2]). Matched datasets consisting of 16S rRNA reads, WGS reads, and WGS assembled contigs were downloaded from the 16S trimmed dataset and the HMIWGS/HMASM dataset respectively. For the WGS reads dataset, the analysis was run on the paired 1 read file.

**Tara ocean viromes**. Tara oceans viromes were downloaded from European Nucleotide Archive (ENA) at EMBL and consisted of 43 viromes from 43 samples at 26 locations across the world's oceans collected during the Tara Oceans (2009-2012) scientific expedition (Supplemental Table 3) [[30]](https://paperpile.com/c/8kIEbl/rCFoW). Metadata for the samples was downloaded from PANGAEA [[35]](https://paperpile.com/c/8kIEbl/LNPBO). These samples were derived from multiple depths including: 16 surface samples (5-6 meters), 18 deep chlorophyll maximum samples (DCM; 17-148 meters), and one mesopelagic sample (791 meters). Quality control procedures were applied according to methods described by Brum and colleagues [[30]](https://paperpile.com/c/8kIEbl/rCFoW).

**CAMI Human microbiome project toy dataset**

The human microbiome project toy dataset from the Critical Assessment of Metagenome Interpretation 2nd Challenge was downloaded from <https://data.cami-challenge.org/participate>. This dataset is composed of 49 simulated pacBio reads from five different body sites of the human host, namely gastrointestinal tract, oral cavity, airways, skin and urogenital tract.

**RESULTS AND DISCUSSION**

**Libra computational strategy**. Libra uses Hadoop MapReduce to perform massive all-vs-all sequence comparisons between next-generation sequence (NGS) datasets. Libra uses a scalable algorithm and efficient resource usage to make all-vs-all comparisons feasible on large datasets. Hadoop allows parallel computation over distributed computing resources via its simple programming interface called *MapReduce*, while hiding much of the complexity of distributed computing (e.g. node failures) for robust fault-tolerant computation. Taking advantage of Hadoop, Libra can scale to larger input datasets and more computing resources. Furthermore, many cloud providers such as Amazon and Google offer Hadoop clusters on a pay-as-you-go basis, allowing scientists to scale their Libra computations to match their datasets and budgets.

Libra is implemented using three different MapReduce jobs — 1) k-mer histogram construction, 2) inverted index construction, and 3) distance matrix computation. Fig 1 shows a workflow of the Libra algorithm.

**Figure 1. The Libra Workflow.**

Libra consists of three MapReduce jobs (yellow boxes) — 1) Libra constructs a k-mer histogram of the input samples for load-balancing. The k-mer histogram of the input samples is computed in parallel by running multiple Map tasks and a Reduce task that combines their results; 2) Libra performs the inverted index construction in parallel. In the Map phase, a separate Map task is spawned for every data block in the input sample files. Each Map task generates k-mers from the sequences stored in a data block then passes them to the Reduce tasks. Each Reduce task then counts k-mers it receives and produces an index chunk; 3) In the distance matrix computation, the work is split by partitioning the k-mer space in the beginning of a MapReduce job. The k-mer histogram files for input samples are loaded and merged, and the k-mer space is partitioned according to the k-mer distributions. A separate Map task is spawned for each partition to perform the computation in parallel and merged to produce the complete distance matrix.

**Libra distance computation.**Libra provides three distance metrics — Cosine Similarity, Bray-Curtis and Jensen-Shannon. In this paper, we demonstrate Cosine Similarity as the default distance metric. This distance uses a vector space model to compute the distance between two NGS samples based on their k-mer composition and abundance, while simultaneously normalizing for sequencing depth.

**Cosine Similarity allows for an accurate and normalized comparison of metagenomes.**

Jaccard and Bray-Curtis distance have been extensively used to compare metagenomes based on their sequence signature [[10,11,13]](https://paperpile.com/c/8kIEbl/M6kKE+RWhtK+LTd9P). While Mash only computes the Jaccard distance between samples, SIMKA and Libra implement several classical ecology distances, allowing the user to choose the best-suited distance for the considered dataset [[13]](https://paperpile.com/c/8kIEbl/LTd9P). Moreover, Libra implements a distance metric, Cosine Similarity that is widely used in document clustering and information retrieval. This distance metric was previously used to evaluate the accuracy of methods to reconstruct genomes from “virtual metagenomes” derived from 16S rRNA data based on shared KEGG orthologous gene counts [(Okuda et al. 2012)](https://paperpile.com/c/8kIEbl/YE8x), but has not been applied in analyzing sequence signatures between metagenomes. Users can also weight k-mers based on their abundance in Libra (using boolean weighting, natural weighting and logarithmic weighting) to account for differences in microbial community composition and sequencing effort as detailed below.

We tested these effects by varying: (1) the size of the datasets, (2) depth of sequencing, (3) the abundance of dominant microbes in the community, and (4) genetic composition of the community by adding in an entirely new organism (in our case we added archaea). We constructed simulated metagenomes and compared Libra’s distance based on the cosine similarity against those from Mash and SIMKA. Simulated datasets were derived from genomic DNA from a staggered mock community of bacteria obtained from the human microbiome consortium and sequenced deeply using the Ion Torrent sequencing platform (80 million reads, see methods).

First, we examined the effect of the size of the dataset by using GemSim [[33]](https://paperpile.com/c/8kIEbl/ftnTG) to obtain a simulated metagenome composed of 1 million reads (454 sequencing) from the mock community and duplicating that dataset 2x and 10x. Overall, we found that altering the size of the metagenome (by duplicating the data) had no effect on the distance between metagenomes for Mash, SIMKA, or Libra. In each case the distance of the duplicated datasets to the 1x mock community was less than 0.0001 (data not shown).

Because metagenomes don’t scale exactly with size and instead have an increasing representation of low-abundance organisms, we created a second simulated dataset from the mock community using GemSim [[33]](https://paperpile.com/c/8kIEbl/ftnTG) 0.5, 1, 5, and 10 million reads (454 sequencing) to mimic the effect of reducing the sequencing. Given the abundance of organisms in the mock community, the 0.5 M read dataset is mainly comprised of dominant species. *Because SIMKA normalizes all samples to the lowest read count, no* changes between samples when using Jaccard and Bray-Curtis distances (Fig 2A). On the contrary*, Mash and Libra (natural weighting) are taking into account all of the reads in the metagenomes, therefore they measure a larger difference when you compare the smallest (0.5M read sample) and largest (10 million read sample).* . These results suggest that Libra (natural weighting) and Mash are appropriate for comparing datasets at different sequencing depths, whereas using SIMKA could lead to undesired effects.

**Figure 2. Analysis of simulated metagenomes using Mash, SIMKA and Libra.**

1. Distance to staggered mock community simulated metagenome composed of 10 million reads (mock1 10M), for simulatedmetagenomes of same community sequenced at various depth. Simulated metagenomes (454 sequencing) were obtained using GemSim and the known abundance profile of the staggered mock community (see Supplemental Table 1). In order to mimic various sequencing depth, the simulated metagenomes were generated at 0.5, 1, 5 or 10 million reads (noted mock1 0.5M; mock1 1M; mock1 5M; mock1V2 10M). The distances between the 4 simulated metagenomes and a 10 million read simulated metagenome (mock1 10M) were computing using Mash, SIMKA (Jaccard and Bray-curtis distance) and Libra (natural weighting).
2. Distance to staggered mock community simulated metagenome (mock 1), for simulated metagenomes from increasingly distant communities. The mock 1 relies on the known abundance profile from the staggered mock community. The mock 2 community profile was obtained by randomly inverting 3 species abundance from mock 1 profile. The mock 3 profile was obtained by randomly inverting 2 species abundances from mock 2 profile. Finally mock 4 profile was obtained by adding high abundance archeal genomes not present in any the other mock communities. Simulated metagenomes (454 sequencing) were generated using GemSim at 10 million reads. The distance between the mock 1 community to mock 2, mock 3, mock 4 and a replicate community (mock1 V2) was computed using Mash, SIMKA (Jaccard and Bray-curtis distance) and LIBRA (cosine distance, natural and logarithmic weighting).

In addition to natural variation in population-level abundances, artifacts from sequencing can result in high-abundance k-mers. Libra allows users to select the optimal methodology for weighting high abundance k-mers in their datasets including boolean, natural, and logarithmic. These options for weighting k-mers are important for different biological scenarios as described below and shown in simulated datasets. To examine the effect of weighting, we compared and contrasted the natural and logarithmic weight in Libra, with other distances obtained from Mash and SIMKA (Jaccard and Bray-Curtis). We also examined the effect of adding an entirely new species by spiking a simulated dataset with sequences derived from archaea (that were not present in the mock community). The simulated datasets (454 technology) were comprised of the staggered mock community (mock 1), the mock community with alterations in a few abundant species (mock 2), the mock community with many alterations in abundant species (mock 3), and mock 3 with additional sequences from archaea to alter the genetic composition of the community (mock 4) (see Supplemental Table 1). The resulting data showed that Libra (logarithmic weighting) shows a stepwise increase in distance among the mock communities (Fig 2B). This suggests that logarithmic weighting in Libra allows for a comparison of distantly related microbial communities. Mash also shows a stepwise distance between communities, but is compressed relative to Libra, making differences less distinct. SIMKA (Bray-Curtis and Jaccard) and Libra (cosine distance, natural weighting) reach the maximum difference between mock communities 3 and 4 (Fig 2B). This indicates that these distances are more appropriate when comparing metagenomes with small fluctuations in the community (e.g., data from a time-series analysis), whereas Libra (cosine distance, logarithmic weighting) can be used to distinguish metagenomes that vary in both genetic composition and abundance over a wide-range of species diversity by dampening the effect of high-abundance k-mers. Because of this important difference, we used the cosine distance with the logarithmic weighting in all subsequent analyses. Further, we also found that cosine distance provided the fastest computation among all distance metrics (see Methods). We confirmed these findings using Illumina simulated datasets (Supplemental Figure 4A), to show that these results are consistent across short read technologies.

Given the availability of long read (~10K) sequencing technologies like Oxford Nanopore and PacBio sequencing, we repeated the analyses above on simulated long read data (Supplemental Figure 4B). We show that simulated PacBio long read data for the mock community derived from SimLoRD [(Stöcker et al. 2016)](https://paperpile.com/c/8kIEbl/3qrw) shows a similar stepwise distance pattern between each of the mock communities (Supplemental Figure 4B), but has a higher overall distance between mock 1 and each of the mock communities (mock 2 - 4) likely due to the high simulated random error rate compared to simulated short read data.

**Libra accurately profiles differences in bacterial diversity and abundance in amplicon and WGS datasets from the human microbiome.**

Microbial diversity is traditionally assessed using two methods: the 16S rRNA gene to classify bacterial and archaeal groups at the genus to species level, or whole genome shotgun sequencing (WGS) for finer taxonomic classification at the species or subspecies level. Further, WGS datasets provide additional information on functional differences between metagenomes. Here we compare and contrast the effect of different algorithmic approaches (Mash vs Libra vs SIMKA), distance metric (Libra vs SIMKA), data type (16S rRNA vs WGS), and sequence type (WGS reads vs assembled contigs) in analyzing data from 48 samples across 8 body sites from the Human Microbiome Project. Specifically, we examine matched datasets (16S rRNA reads, WGS reads, and WGS assembled contigs) classified as urogenital (posterior fomix), gastrointestinal (stool), oral (buccal mucosa, supragingival plaque, tongue dorsum), airways (anterior nares), and skin (retroauricular crease left and right) ([See Supplemental Table 1]).

Because the HMP datasets represent microbial communities, abundant bacteria will have more total read counts than rare bacteria in the samples. Thus, each sample can vary by both taxonomic composition (the genetic content of taxa in a sample) and abundance (the relative proportion of those taxa in the samples). Importantly, the 16S rRNA amplicon dataset is useful in showing how well each algorithm performs in detecting and quantifying small-scale variation for single a gene at the genus-level, whereas the WGS dataset demonstrates the effect of including the complete genetic content and abundance of organisms at the species-level in a community [[37]](https://paperpile.com/c/8kIEbl/yybmX). Also, we examine differences in each algorithm when read abundance is excluded using assembled contigs that only represent the genetic composition of the community.

Using the 16S rRNA reads, both Mash and Libra clustered samples by broad categories but not individual body-sites (Fig 3A and B). Similar to what is described in previous work [[13]](https://paperpile.com/c/8kIEbl/LTd9P), samples from the airways and skin co-cluster, whereas other categories including urogenital, gastrointestinal, and oral are distinct [[13]](https://paperpile.com/c/8kIEbl/LTd9P). These results indicate that limited variation in the 16S rRNA gene may only allow for clustering for broad categories. Further, the Mash algorithm shows lower overall resolution (Fig 3A) as compared to Libra (Fig 3B). Indeed, amplicon sequencing analysis is not an original intended use of Mash, given that it reduces the dimensionality of the data by looking at presence/absence of unique k-mers, whereas Libra examines the complete dataset accounting for both composition in organisms and their abundance. In contrast, SIMKA (Jaccard-ab and Bray-Curtis) failed to cluster samples by broad categories: some skin samples are found associated with stool and formix samples (Fig 3C and D). Moreover, SIMKA Jaccard-ab fails to cluster the mouth samples together (Fig 3C). This result suggests that applying SIMKA and these well-used distance metrics are not appropriate for these datasets.

**Figure 3. Clustering of HMP 16S rRNA datasets using Mash, Libra and SIMKA.**

48 Human metagenomic samples from the HMP projects clustered by Mash (A), Libra (B) or SIMKA using Jaccard-ab (C) and Bray-Curtis distances (D) from 16s sequencing runs . The samples were clustered using Ward’s method on their distance scores. *Mash, SIMKA, and Libra report distance in the same range (0-1).* Heat maps showing the pairwise dissimilarity between samples, were therefore scaled between 0 (green) and 1 (red). A key below the heatmap colors the samples by body sites.

When using WGS reads, both Mash and Libra show enhanced clustering by body-site (Fig 4A and B), however Mash shows decreased resolution (Fig 4A) as compared to Libra (Fig 4B). Again, these differences reflect the effect of using all of the read data (Libra) rather than a subset (Mash). The effect of using all of the read data compared to a subset (when sketching in Mash) has been previously described in Benoit *et* *al*. [(Benoit et al. 2016)](https://paperpile.com/c/8kIEbl/LTd9P). Importantly, the Libra algorithm depends on read abundance that provides increased resolution for interpersonal variation as seen in skin samples (Fig 4B). Similar to the 16S rRNA datasets, SIMKA (Jaccard-ab and Bray-Curtis) failed to cluster the samples by body site, where some skin and stool samples cluster with formix samples (Fig 4C and D). Similarly, SIMKA Jaccard-ab also fails to cluster the mouth samples together (Fig 4C). Overall SIMKA shows an enhanced clustering by body-site using WGS data compared to the 16S rRNA data using these distance metrics, however the clustering is still not accurate. In order to confirm the independence of these result toward the sequencing technology, we performed the same experiment on the *CAMI HMP “toy dataset” (simulated PacBio long reads) [supplemental Figure 4]. This analysis shows that each of the tools is able to cluster the samples broadly by body site. However there are small misclassifications shared across all tools, suggesting that the increased error rate for this technology could have a limited impact on k-mer based analytics.*

**Figure 4. Clustering of WGS samples using Mash, and Libra and SIMKA.**

48 Human metagenomic samples from the HMP projects clustered by Mash (A), Libra (B) or SIMKA using Jaccard-ab (C) and Bray-Curtis distances (D) from whole genome shotgun sequencing runs. The samples were clustered using Ward’s method on their distance scores. Heat maps illustrate the pairwise dissimilarity between samples, scaled between 0 (green) and 1 (red). A key below the heatmap colors the samples by body sites.

When abundance is taken out of the equation by using assembled contigs ([See Supplemental Figure 3]) Mash performs well in clustering distinct body sites whereas Libra shows discrepancies and less overall resolution. Thus, Libra requires reads rather than contigs to perform accurately and obtain high-resolution clustering (Fig 4). SIMKA (Jaccard-ab and Bray-Curtis) was not able to distinguish any assembled datasets and scored all sample-to-sample distances to the maximum, even considering presence-absence distance metric proposed by SIMKA (data not shown). This phenomenon may be explained by the normalization method used by SIMKA, which does not provide enough data to compare the samples when normalized by the smallest number of contigs (in our dataset 69).

**Libra allows for ecosystem-scale analysis: clustering the Tara ocean viromes to unravel global patterns.**

To demonstrate the scale and performance of the Libra algorithm, we analyzed 43 Tara Ocean Viromes (TOV) from the 2009-2011 Expedition [[30]](https://paperpile.com/c/8kIEbl/rCFoW) representing 26 sites, 43 samples, and 4.2 billion reads from the global ocean (see methods). Phages (viruses that infect bacteria) are abundant in the ocean [[38]](https://paperpile.com/c/8kIEbl/xHuWk) and can significantly impact environmental processes through host mortality, horizontal gene transfer, and host-gene expression. Yet, how phages change over space and time in the global ocean and with environmental fluxes is just beginning to be explored. The primary challenge is the majority of reads in viromes (often > 90%) do not match known proteins or viral genomes [[3]](https://paperpile.com/c/8kIEbl/6r8Pm) and no conserved genes like the bacterial 16S rRNA gene exist to differentiate populations. To examine known and unknown viruses simultaneously, viromes are best compared using sequence signatures to identify common viral populations.

Two approaches exist to cluster viromes based on sequence composition. The first approach uses protein clustering to examine functional diversity in viromes between sites [[3,30,39]](https://paperpile.com/c/8kIEbl/6r8Pm+nWzc3+rCFoW). Protein clustering, however, depends on accurate assembly and gene finding that can be problematic in fragmented and genetically diverse viromes [[40]](https://paperpile.com/c/8kIEbl/AtFGg). Further, assemblies from viromes often only include a fraction of the total reads (e.g., only ⅓ in TOV [[30]](https://paperpile.com/c/8kIEbl/rCFoW)). To examine global viral diversity in the ocean using all of the reads we examined TOV using Libra. The complete pairwise analysis of ~4.2 billion reads in the TOV dataset [[30]](https://paperpile.com/c/8kIEbl/rCFoW) finished in 18 hours using a 10-node Hadoop cluster (see Methods and Table 2). Importantly, Libra exhibits remarkable performance in computing similarity scores, wherein k-mer matches for all TOV completed within 1.5 hours (see Table 2). This step usually represents the largest computational bottleneck for bioinformatics tools that compute pairwise distances between sequence pairs for applications such as hierarchical sequence clustering [[41–44]](https://paperpile.com/c/8kIEbl/I2QyH+nAk4m+7KBba+OZW8n).  *A direct comparison of the runtime of the SIMKA, MASH and Libra is not possible given that each tool is tuned to a different computational architecture with a different number of servers and total CPU/memory (Mash runs on a single server; SIMKA runs on an HPC; and Libra on Hadoop).*

***Table 2. Execution times for the Libra based on the Tara Ocean Virome (TOV) dataset.***

| **Stage** | **Execution Time** |
| --- | --- |
| Preprocessing (k-mer histogram construction + Inverted index construction) | 16:32:55 |
| Distance matrix computation | 1:24:27 |
| Total | 17:57:22 |

Overall, we found that viral populations in the ocean are largely structured by temperature in four gradients (Fig 5) similar to their bacterial hosts [[2]](https://paperpile.com/c/8kIEbl/96Fzn). Interestingly, samples from different Longhurst Provinces but the same temperature gradient cluster together. Also, water samples from the surface (SUR) and deep chlorophyll maximum (DCM) at the same station, cluster more closely together than samples from the same depth at nearby sites (Fig 5). Also noteworthy, samples that were derived from extremely cold environments (noted as C0 in Fig 4) lacked similarity to all other samples (at a 30% similarity score), indicating distinctly different viral populations. These samples include a mesotrophic sample that have previously been shown to have distinctly different viral populations than surface ocean samples [[45]](https://paperpile.com/c/8kIEbl/MYHIH). Taken together, these data indicate that viral populations are structured globally by temperature, and at finer resolution by station (for surface and DCM samples) indicating that micronutrients and local conditions play an important role in defining viral populations.

**Figure 5. Visualizing the genetic distance among marine viral communities using Libra.**

Distance computed from 43 TOV from the 2009-2012 Tara Oceans Expedition. Lines (edges) between samples represent the similarity and are colored and thickened accordingly. Lines with insignificant similarity (less than 30%) are removed. Each of the sample names are color coded by Longhurst Province. Inner circles show temperature ranges. Sample names show the temperature range, station, and depth as indicated on the legend.

**INNOVATIONS**

Scientific collaboration is increasingly data driven given large-scale next generation sequencing datasets. It is now possible to generate, aggregate, archive, and share datasets that are terabytes and even petabytes in size. Scalability of a system is becoming a vital feature that decides feasibility of massive ‘omic’s analyses. In particular, this is important for metagenomics where patterns in global ecology can only be discerned by comparing the sequence signatures of microbial communities from massive ‘omics datasets, given that most microbial genomes have not been defined. Current algorithms to perform these tasks run on local workstations or high-performance computing architectures that cannot scale. Libra presents three main innovations: the use of a scalable Apache Hadoop framework enabling massive dataset comparison, linear calculations for complex distance metrics allowing for high accuracy and clustering of the metagenomes based on their k-mer content, and a web-based tool imbedded in the CyVerse advanced cyberinfrastructure through iMicrobe (<http://imicrobe.us>) for broader use of the tool in the scientific community. The work described here is the first step in implementing a cloud-based resource for comparative metagenomics that can be broadly used by scientists to analyze large-scale shared data resources. Moreover, the code can be ported to any Hadoop cluster (e.g., Wrangler at TACC, Amazon EMR or private Hadoop clusters). This computing paradigm is consistent with recent efforts to increase the accessibility of big datasets in the cloud, such as the Pan Cancer Analyses of Whole Genomes Project [[46]](https://paperpile.com/c/8kIEbl/Xwz4n).

**METHODS**

**Libra Algorithm Detailed Description**:

**k-mer size**. **Libra calculates the distances between samples based on their k-mer composition. Canonical representation of the k-mer is used to reduce the number of stored k-mers.**Several considerations should be taken into account for choosing the k-mer size *k*. Larger values of *k* result in fewer matches due to sequencing errors and fragmentary metagenomic data. However, smaller values of *k* give less information about the sequence similarities. In Libra, *k* is a configurable parameter chosen by the user, and is set by default to*k* equal to 21. This value was reported to be at the inflection point where the k-mer matches move from random to representative of the read content, and is generally resilient to sequencing error and variation [[1,2]](https://paperpile.com/c/hr5db9/0cXq+NDYT).

**Distance Matrix Computation**. Libra provides three distance metrics — Cosine Similarity, Bray-Curtis and Jensen-Shannon. The default distance metric in Libra is the Cosine Similarity.

**Cosine Similarity metric**. Libra constructs a vector $v_{s}$ for each sample *s* from the weight of each k-mer *k* in the sample ($w_{k.s}$). Each dimension in the vector corresponds to the weight of the corresponding k-mer:

$$v_{s}=(w_{k1,s},w_{k2,s},w_{k3,s},...,w_{kn,s})$$

The weight of a k-mer in a sample ($w_{ks}$) can be derived from frequency of the k-mer ($f_{ks}$) in several ways. The most simplest way is using the raw frequency of the k-mer (${w_{ks}=f}_{ks}$), called *Natural Weighting*. Another way is using *Logarithmic Weighting* ($w_{ks}=1+log(f_{ks})$) to not give too much weight to highly abundant k-mers. In this weighting the weight $w_{ks}$ grows logarithmically with the frequency $f_{ks}$, reducing the effect on the distance of highly abundant k-mers caused by sequencing artifacts.

Once their vectors have been constructed, the distance between two samples ($s_{1}$ and $s_{2}$) is derived using distance metrics. For example, the distance between the two samples using Cosine Similarity is determined as following:

$$Distance(s_{1},s_{2})=1-CosineSimilarity(s_{1},s_{2})$$

$$=1-cos(v_{s1},v_{s2})=1-\frac{v_{s1} \cdot v_{s2}}{{||v_{s1}||} \times{||v_{s2}||}}=1-\frac{D_{s1,s2}}{M_{s1}\times M_{s2}}$$

$$where, D_{s1,s2}=v_{s1}\cdot v_{s2}=\sum_{i\in s1\cap s2} w_{ki,s1}\times w_{ki,s2},$$

$M_{s}={||v_{s}||}=\sqrt{\sum_{i\in s} {(w_{ki,s})}^{2}}$

In other words, $D_{s1,s2}$ is the dot product of the vectors $v_{s1}$and $v_{s2}$, and $M_{s}$ is the magnitude (length) of the vector $v_{s}$. The distance between two NGS samples is the cosine of the angle between their vectors $v_{s}$, the magnitude of the vector $M_{s}$ is not taken into account in the metric therefore normalizing samples with different numbers of total base pairs.

**Inverted Index Construction**. A naïve implementation would require the storage of one vector with 4*^k^*  dimensions per sample, with *k* the k-mer length. For a k of 20, each vector would have more than one million dimensions. To reduce that, Libra stores and computes the distance on a a single *inverted index* with the k-mer frequencies from multiple samples and performs the distance computation on the index directly. The inverted index is indexed by k-mer, and contains the identifiers of the samples that contain that k-mer and its frequency for each sample. The key of the index is a k-mer and the value is a list of pairs, each of which contains a sample identifier and the frequency of the k-mer in the sample.

$index record = k₋mer : \{<sample₋id, frequency>,<sample₋id, frequency>...\}$

The records in the index are stored in an alphabetical order by k-mer, allowing the record for a particular k-mer to be found via binary search. The k-mer record contains the k-mer frequency in each sample, not the weight, to allow for different weighting functions to be applied during distance matrix computation.

**Sweep line algorithm**. To compute the distance between two samples $S_{1}$ and $S_{2}$, Libra must compute the three values $D_{s1,s2}$*,* $M_{s1}$*, and* $M_{s2}$*.* The values are calculated by scanning through the vectors $v_{s1}$and $v_{s2}$ and computing the values. The time for the distance matrix computation is proportional to the number of dimensions (the number of k-mers) in the two vectors. In general, computing all-vs-all comparisons on n samples would require $n\times(n-1)/2$ vector scans, which becomes prohibitively expensive as *n* gets large. Libra uses a sweep line algorithm [38] to greatly reduce the computational time.

$D_{s1,s2}$ $v_{s1}$ $v_{s2}$

The sweep line algorithm only requires a single scan of all vectors to compute the distance of all pairs of samples ([See Supplemental Figure 1]). Briefly, Libra sweeps a line through all the vectors simultaneously starting with the first component. Libra outputs a record of the non-zero values of the following format:

$$record = k₋mer : \{<sample₋id, weight>, <sample₋id, weight>, ...\}$$

Libra then moves the sweep line to the next component and performs the same operation. From the output records, contributions to $M_{s}$ for each sample in the record are computed and accumulated. Contributions to $D$ are also computed from the record by extracting sample pairs. For example, the record {${<s}_{1},x>,{<s}_{2},y>,<s_{4},z>$} has three sample pairs $($ $s_{1}s_{2}), (s_{1}s_{4}) and (s_{2}s_{4})$. Libra then computes contribution to $D$ for each pair, e.g. $x\times y$ is added to $D_{s1,s2}$, $x\times z$ is added to $D_{s1,s4}$, and $y\times z$ is added to $D_{s2,s4}$. Using this method, Libra computes the distances of every sample pairs in an input dataset in linear time. Other distance metrics, such as Bray-Curtis and Jensen-Shannon, can also be computed in the same fashion.

The sweep algorithm is particularly easy to implement on an inverted index; it consists of simply stepping through the (sorted) k-mers. Furthermore, the sweep algorithm is easily parallelized. The k-mer space is partitioned and a separate sweep is performed on each partition computing the contributions of its k-mer frequencies to the $D$ and $M$ values. At the end of the computation the intermediate $D$ and $M$ values are combined together to produce the final $D$ and $M$ values and thereby the distance matrix. Each sweep uses binary search to find the first k-mer in the partition.

**Terabyte Sort**. Libra groups samples automatically based on the number and size (by default 4GB per group). Similar to Terabyte Sort [[3], t](https://paperpile.com/c/hr5db9/VRKp)he index records are partitioned by k-mer ranges and the records in each partition is stored in a separate *chunk file*. All k-mers in partition $n$ appear before the k-mers partition$n+1$ in lexicographic order. This breaks computation and IO down into smaller tasks, so that workload for creating an index can be distributed across several machines.

**k-mer space partitioning**. Both the inverted index construction and the distance matrix computation require partitioning the k-mer space so that different partitions can be processed independently. For the partitioning to be effective, the workload should be balanced across the partitions. Simply partitioning into fixed-size partitions based on the k-mer space will not ensure balanced workloads, as the k-mers do not appear with uniform frequency. Some partitions may have more k-mer records than others, and thereby incur higher processing costs. Instead, the partitions should be created based on the k-mer distribution, so that each partition has roughly the same number of records ([See Supplemental Figure 2]).

Computing the exact k-mer distribution across all the samples is too expensive in both space and time, therefore Libra approximates the distribution instead. A histogram is constructed using the first 6 letters of the k-mers in each sample, which requires much less space and time to compute. In practice, partitioning based on this histogram sufficiently partitions the k-mer space so that the workloads are adequately balanced across the partitions.

**Scalability benchmarking for Libra.** We used synthetic datasets for a scalability benchmark. Each dataset contains 10 billion bytes (approximately 9.3 GB). We used four datasets consisting of 10 (93GB), 20 (186GB), 30 (279GB) and 40 (372GB) samples in the benchmark. Each experiment was run three times, and an average of the three runs reported ([See Supplemental Table 4 for details]). The runtime of Libra increased linearly with increased input volume (Figure 6) . This shows that Libra efficiently handles increased volume of input and efficiently computes distances between all sample pairs while the number of sample pairs increases quadratically.

**Figure 6** **Scalability testing for Libra.** Four datasets consisting of 10, 20, 30 and 40 samples (total sizes of 93GB, 186GB, 279GB and 372GB, respectively).

**Benchmarking runtimes of different distance metrics in Libra.** We used the same synthetic dataset with 40 samples (372GB in total) in the scalability benchmarking (Figure 7). We measured the runtimes of Libra for the different distance metrics. Once the index is constructed all distance metrics can be calculated using that index. .Thus, runtimes of the inverted index construction for the different metrics are the same. Each experiment was run three times, and an average of the three runs reported ([See Supplemental Table 4 for details]). Differences in runtimes are mainly due to different computational workload of distance metrics (Figure 7). For example, Jensen-Shannon requires more multiplications and divisions in nested loops than cosine similarity, incurring more computational workload. Yet, distance matrix computation with Jensen-Shannon took only 12.64% of total runtime.

**Figure 7**. **Runtime for different distance metrics**. Runtimes for three different distance metrics (Cosine Similarity, Bray-Curtis and Jensen-Shannon) in Libra with 40 samples of input (372GB in total). **Advanced cyberinfrastructure for Libra in iMicrobe**. To improve access to Libra we made it available on the iMicrobe website (https://[www.imicrobe.us](http://www.imicrobe.us/)). A researcher with a CyVerse account can run Libra on iMicrobe by filling-out a simple web form specifying the input files and parameters. Input files are selected from the CyVerse Data Store where they have either been uploaded by the user to their home directory or are part of the iMicrobe Data Commons. When a job is submitted, the user is presented with the status of the job, and on completion the output files and visualization of results. To deploy Libra on iMicrobe, we developed a job dispatch service to automate execution of Libra on a University of Arizona Hadoop cluster. The service is written in NodeJS and accepts a JSON description of the job inputs and parameters, stages the input files onto the UA Hadoop cluster, executes Libra with the given parameters, and transfers the resulting output files to the user's home directory in the CyVerse Data Store. The service provides a RESTful interface that mimics the Agave API Jobs service and is secured using an Agave OAuth2 token. Source code is located at <https://github.com/hurwitzlab/occ-plan-b>.

**Experimental Environment Description:**

**Mash and SIMKA configurations.** Mash v1.1 was run on the metagenomic datasets with the following parameters: -r –s 10000 –m 2 [19]. The analysis of assemblies was run without the parameter “-r”, used for short sequences.

SIMKA v1.3.2 was run on the metagenomic datasets with the following parameters: -abundance-min 2 -max-reads [MINCOUNT] -simple-dist -complex-dist, where [MINCOUNT] is the smallest sequence count across the analysed samples.

**Hadoop cluster configuration**. The Libra experiments described in the paper were performed on a Hadoop cluster consisting of 10 physical nodes (9 MapReduce worker nodes). Each node contains 12 CPUs and 128 GB of RAM, and is configured to run a maximum of 7 YARN containers simultaneously with 10 GB of RAM per container. The remaining system resources are reserved for the operating system and other Hadoop services such as Hive or Hbase.

**FUNDING**

This work was supported by the National Science Foundation award #1640775 to BLH and JHH. System support and access for the Hadoop cluster was provided by University of Arizona Information Technology Services. System support and access for the Wrangler cluster was provided by Texas Advanced Computing Center. The following reagent was obtained through BEI Resources, NIAID, NIH as part of the Human Microbiome Project: Genomic DNA from Microbial Mock Community B (Staggered, High Concentration), v5.2H, for Whole Genome Shotgun Sequencing, HM-277D.

*Competing interests*: The authors declare no competing interests.

**Availability and Implementation**:

**Project home page:** Program binary, source code and documentation for Libra are available in Github ([https://www.github.com/iychoi/Libra](https://www.github.com/iychoi/kogiri)); Libra web-based App is in iMicrobe under Apps ([http://imicrobe.us](http://imicrobe.us/)); code to implement the Libra web-based App is in Github (<https://github.com/hurwitzlab/occ-plan-b>); **Operating system(s):** MapReduce 2.0 (Apache Hadoop 2.3.0 or above); **Programming language:** Java 7 (or above); **Other requirements:** none; **License:** Apache License Version 2.0; **Any restrictions to use by non-academics:** no license needed. Libra has been registered with the SciCrunch.org database under reference ID: SCR_016608.

**REFERENCES**

[1. Yooseph S, Sutton G, Rusch DB, Halpern AL, Williamson SJ, Remington K, et al. The Sorcerer II Global Ocean Sampling expedition: expanding the universe of protein families. PLoS Biol. 2007;5:e16.](http://paperpile.com/b/8kIEbl/k1BC4)

[2. Sunagawa S, Coelho LP, Chaffron S, Kultima JR, Labadie K, Salazar G, et al. Structure and function of the global ocean microbiome. Science [Internet]. sciencemag.org; 2015;348. Available from:](http://paperpile.com/b/8kIEbl/96Fzn) <http://www.sciencemag.org/content/348/6237/1261359.abstract>

[3. Hurwitz BL, Sullivan MB. The Pacific Ocean Virome (POV): a marine viral metagenomic dataset and associated protein clusters for quantitative viral ecology. PLoS One. 2013;8:e57355.](http://paperpile.com/b/8kIEbl/6r8Pm)

[4. Dubinkina VB, Ischenko DS, Ulyantsev VI, Tyakht AV, Alexeev DG. Assessment of k-mer spectrum applicability for metagenomic dissimilarity analysis. BMC Bioinformatics. bmcbioinformatics.biomedcentral. …; 2016;17:38.](http://paperpile.com/b/8kIEbl/ZdF41)

[5. Teeling H, Waldmann J, Lombardot T, Bauer M, Glockner FO. TETRA: a web-service and a stand-alone program for the analysis and comparison of tetranucleotide usage patterns in DNA sequences. BMC Bioinformatics. 2004;5:163.](http://paperpile.com/b/8kIEbl/heFng)

[6. Wu Y-W, Ye Y. A novel abundance-based algorithm for binning metagenomic sequences using l-tuples. J Comput Biol. online.liebertpub.com; 2011;18:523–34.](http://paperpile.com/b/8kIEbl/Z5qS4)

[7. Fofanov Y, Luo Y, Katili C, Wang J, Belosludtsev Y, Powdrill T, et al. How independent are the appearances of n-mers in different genomes? Bioinformatics. Oxford University Press; 2004;20:2421–8.](http://paperpile.com/b/8kIEbl/bqvNX)

[8. Maillet N, Lemaitre C, Chikhi R, Lavenier D, Peterlongo P. Compareads: comparing huge metagenomic experiments. BMC Bioinformatics. bmcbioinformatics.biomedcentral. …; 2012;13 Suppl 19:S10.](http://paperpile.com/b/8kIEbl/BqLrD)

[9. Maillet N, Collet G, Vannier T, Lavenier D, Peterlongo P. Commet: Comparing and combining multiple metagenomic datasets. 2014 IEEE International Conference on Bioinformatics and Biomedicine (BIBM). ieeexplore.ieee.org; 2014. p. 94–8.](http://paperpile.com/b/8kIEbl/vVxsY)

[10. Ulyantsev VI, Kazakov SV, Dubinkina VB, Tyakht AV, Alexeev DG. MetaFast: fast reference-free graph-based comparison of shotgun metagenomic data. Bioinformatics. Oxford Univ Press; 2016;32:2760–7.](http://paperpile.com/b/8kIEbl/M6kKE)

[11. Ondov BD, Treangen TJ, Melsted P, Mallonee AB, Bergman NH, Koren S, et al. Mash: fast genome and metagenome distance estimation using MinHash. Genome Biol. biorxiv.org; 2016;17:132.](http://paperpile.com/b/8kIEbl/RWhtK)

[12. Broder AZ. On the resemblance and containment of documents. Proceedings Compression and Complexity of SEQUENCES 1997 (Cat No97TB100171) [Internet]. Available from:](http://paperpile.com/b/8kIEbl/9Emr) <http://dx.doi.org/10.1109/sequen.1997.666900>

[13. Benoit G, Peterlongo P, Mariadassou M, Drezen E, Schbath S, Lavenier D, et al. Multiple comparative metagenomics using multiset k-mer counting. PeerJ Comput Sci. PeerJ Inc.; 2016;2:e94.](http://paperpile.com/b/8kIEbl/LTd9P)

[14. Dean J, Ghemawat S. MapReduce: Simplified Data Processing on Large Clusters. Commun ACM. New York, NY, USA: ACM; 2008;51:107–13.](http://paperpile.com/b/8kIEbl/jTfy)

[15. Zaharia M, Chowdhury M, Franklin MJ, Shenker S, Stoica I. Spark: Cluster computing with working sets. HotCloud. static.usenix.org; 2010;10:95.](http://paperpile.com/b/8kIEbl/tdI8)

[16. Guo R, Zhao Y, Zou Q, Fang X, Peng S. Bioinformatics applications on Apache Spark. Gigascience [Internet]. academic.oup.com; 2018; Available from:](http://paperpile.com/b/8kIEbl/GFlP) <http://dx.doi.org/10.1093/gigascience/giy098>

[17. Kolker N, Higdon R, Broomall W, Stanberry L, Welch D, Lu W, et al. Classifying proteins into functional groups based on all-versus-all BLAST of 10 million proteins. OMICS. online.liebertpub.com; 2011;15:513–21.](http://paperpile.com/b/8kIEbl/A552F)

[18. McKenna A, Hanna M, Banks E, Sivachenko A, Cibulskis K, Kernytsky A, et al. The Genome Analysis Toolkit: a MapReduce framework for analyzing next-generation DNA sequencing data. Genome Res. genome.cshlp.org; 2010;20:1297–303.](http://paperpile.com/b/8kIEbl/5S5KM)

[19. Langmead B, Schatz MC, Lin J, Pop M, Salzberg SL. Searching for SNPs with cloud computing. Genome Biol. biomedcentral.com; 2009;10:R134.](http://paperpile.com/b/8kIEbl/NqwE2)

[20. Nguyen T, Shi W, Ruden D. CloudAligner: A fast and full-featured MapReduce based tool for sequence mapping. BMC Res Notes. biomedcentral.com; 2011;4:171.](http://paperpile.com/b/8kIEbl/Ft9hw)

[21. Schatz MC. BlastReduce: high performance short read mapping with MapReduce. University of Maryland, http://cgis cs umd edu/Grad/scholarlypapers/papers/MichaelSchatz pd f [Internet]. cs.umd.edu; 2008; Available from:](http://paperpile.com/b/8kIEbl/sDd86) <https://www.cs.umd.edu/sites/default/files/scholarly_papers/MichaelSchatz_1.pdf>

[22. Schatz MC. CloudBurst: highly sensitive read mapping with MapReduce. Bioinformatics. Oxford Univ Press; 2009;25:1363–9.](http://paperpile.com/b/8kIEbl/mdmoh)

[23. Pandey RV, Schlötterer C. DistMap: a toolkit for distributed short read mapping on a Hadoop cluster. PLoS One. dx.plos.org; 2013;8:e72614.](http://paperpile.com/b/8kIEbl/gl8cP)

[24. Nordberg H, Bhatia K, Wang K, Wang Z. BioPig: a Hadoop-based analytic toolkit for large-scale sequence data. Bioinformatics. Oxford Univ Press; 2013;29:3014–9.](http://paperpile.com/b/8kIEbl/nqMT)

[25. Gao T, Guo Y, Wei Y, Wang B, Lu Y, Cicotti P, et al. Bloomfish: A Highly Scalable Distributed K-mer Counting Framework. 2017 IEEE 23rd International Conference on Parallel and Distributed Systems (ICPADS). ieeexplore.ieee.org; 2017. p. 170–9.](http://paperpile.com/b/8kIEbl/07Nl)

[26. Menon RK, Bhat GP, Schatz MC. Rapid Parallel Genome Indexing with MapReduce. Proceedings of the Second International Workshop on MapReduce and Its Applications. New York, NY, USA: ACM; 2011. p. 51–8.](http://paperpile.com/b/8kIEbl/1e0Mk)

[27. Huang A. Similarity measures for text document clustering. Proceedings of the sixth new zealand computer science research student conference (NZCSRSC2008), Christchurch, New Zealand. academia.edu; 2008. p. 49–56.](http://paperpile.com/b/8kIEbl/6poA)

[28. Michie MG. Use of the Bray-Curtis similarity measure in cluster analysis of foraminiferal data. Math Geol. Kluwer Academic Publishers-Plenum Publishers; 1982;14:661–7.](http://paperpile.com/b/8kIEbl/RAEY)

[29. Lin J. Divergence measures based on the Shannon entropy. IEEE Trans Inf Theory. 1991;37:145–51.](http://paperpile.com/b/8kIEbl/Pllm)

[30. Brum JR, Ignacio-Espinoza JC, Roux S, Doulcier G, Acinas SG, Alberti A, et al. Patterns and ecological drivers of ocean viral communities. Science [Internet]. sciencemag.org; 2015;348. Available from:](http://paperpile.com/b/8kIEbl/rCFoW) <http://www.sciencemag.org/content/348/6237/1261498.abstract>

[31. Goff SA, Vaughn M, McKay S, Lyons E, Stapleton AE, Gessler D, et al. The iPlant Collaborative: Cyberinfrastructure for Plant Biology. Front Plant Sci. ncbi.nlm.nih.gov; 2011;2:34.](http://paperpile.com/b/8kIEbl/3YGkv)

[32. Devisetty UK, Kennedy K, Sarando P, Merchant N, Lyons E. Bringing your tools to CyVerse Discovery Environment using Docker. F1000Res. 2016;5:1442.](http://paperpile.com/b/8kIEbl/luhg4)

[33. McElroy KE, Luciani F, Thomas T. GemSIM: general, error-model based simulator of next-generation sequencing data. BMC Genomics. biomedcentral.com; 2012;13:74.](http://paperpile.com/b/8kIEbl/ftnTG)

[34. Human Microbiome Project Consortium. Structure, function and diversity of the healthy human microbiome. Nature. nature.com; 2012;486:207–14.](http://paperpile.com/b/8kIEbl/Z7p6a)

[35. Diepenbroek M, Grobe H, Reinke M, Schindler U, Schlitzer R, Sieger R, et al. PANGAEA—an information system for environmental sciences. Comput Geosci. Elsevier; 2002;28:1201–10.](http://paperpile.com/b/8kIEbl/LNPBO)

[36. O’Malley O. Terabyte sort on apache hadoop. Yahoo, available online at: http://sortbenchmark org/Yahoo-Hadoop pdf,(May). Citeseer; 2008;1–3.](http://paperpile.com/b/8kIEbl/PYVue)

[37. Watts GS, Youens-Clark K, Slepian MJ, Wolk DM, Oshiro MM, Metzger GS, et al. 16S rRNA gene sequencing on a benchtop sequencer: accuracy for identification of clinically important bacteria. J Appl Microbiol. Wiley Online Library; 2017;123:1584–96.](http://paperpile.com/b/8kIEbl/yybmX)

[38. Bergh O, Borsheim KY, Bratbak G, Heldal M. High abundance of viruses found in aquatic environments. Nature. 1989;340:467–8.](http://paperpile.com/b/8kIEbl/xHuWk)

[39. Hurwitz BL, Brum JR, Sullivan MB. Depth-stratified functional and taxonomic niche specialization in the “core”and “flexible”Pacific Ocean Virome. ISME J [Internet]. nature.com; 2014; Available from:](http://paperpile.com/b/8kIEbl/nWzc3) <http://www.nature.com/ismej/journal/vaop/ncurrent/full/ismej2014143a.html>

[40. Minot S, Wu GD, Lewis JD, Bushman FD. Conservation of gene cassettes among diverse viruses of the human gut. PLoS One. 2012;7:e42342.](http://paperpile.com/b/8kIEbl/AtFGg)

[41. Sun Y, Cai Y, Huse SM, Knight R, Farmerie WG, Wang X, et al. A large-scale benchmark study of existing algorithms for taxonomy-independent microbial community analysis. Brief Bioinform. Oxford Univ Press; 2012;13:107–21.](http://paperpile.com/b/8kIEbl/I2QyH)

[42. Edgar RC. Search and clustering orders of magnitude faster than BLAST. Bioinformatics. 2010;26:2460–1.](http://paperpile.com/b/8kIEbl/nAk4m)

[43. Niu BF, Fu LM, Sun SL, Li WZ. Artificial and natural duplicates in pyrosequencing reads of metagenomic data. BMC Bioinformatics. 2010;11:187.](http://paperpile.com/b/8kIEbl/7KBba)

[44. Cai Y, Sun Y. ESPRIT-Tree: hierarchical clustering analysis of millions of 16S rRNA pyrosequences in quasilinear computational time. Nucleic Acids Res. Oxford Univ Press; 2011;39:e95.](http://paperpile.com/b/8kIEbl/OZW8n)

[45. Hurwitz BL, Brum JR, Sullivan MB. Depth-stratified functional and taxonomic niche specialization in the “core” and “flexible” Pacific Ocean Virome. ISME J. 2015;9:472–84.](http://paperpile.com/b/8kIEbl/MYHIH)

[46. Weinstein JN, Collisson EA, Mills GB, Shaw KRM, Ozenberger BA, Ellrott K, et al. The cancer genome atlas pan-cancer analysis project. Nat Genet. Nature Publishing Group; 2013;45:1113–20.](http://paperpile.com/b/8kIEbl/Xwz4n)
