# Supplementary material for: Libra: scalable k-mer–based tool for massive all-vs-all metagenome comparisons
Source: Gigascience. 2018 Dec 28;8(2):giy165. doi: 10.1093/gigascience/giy165 (PMC6354030; doi:10.1093/gigascience/giy165)
Supplement: Supplemental Files [file giy165_supplemental_files.zip › Supplemental Fig2.pdf]

A

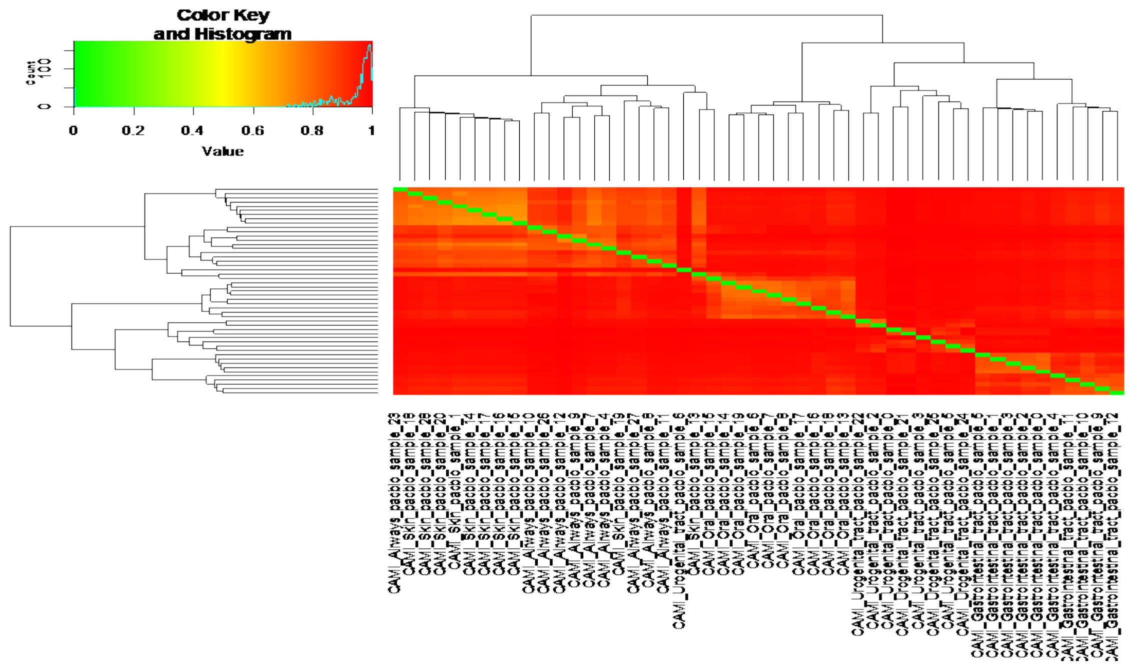
Color Key
and Histogram
Count
0
100
0
0.2
0.4
0.6
0.8
1
Value

B

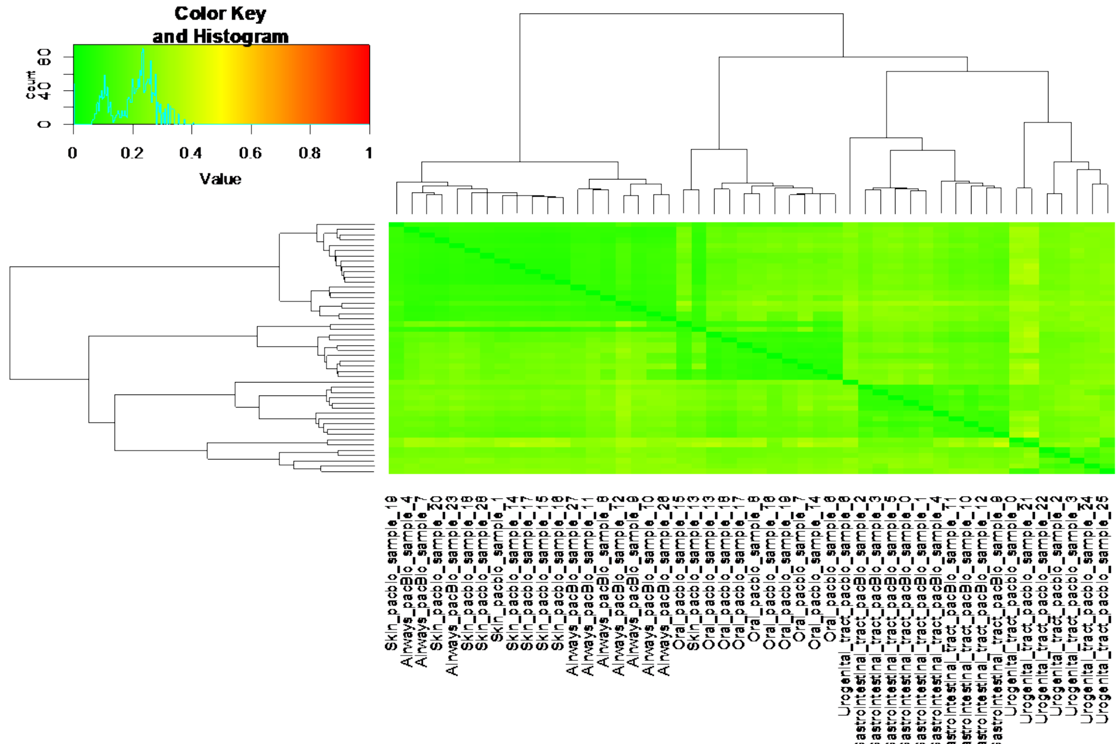
Color Key
and Histogram
Count
0
40
80
0
0.2
0.4
0.6
0.8
1
Value

C

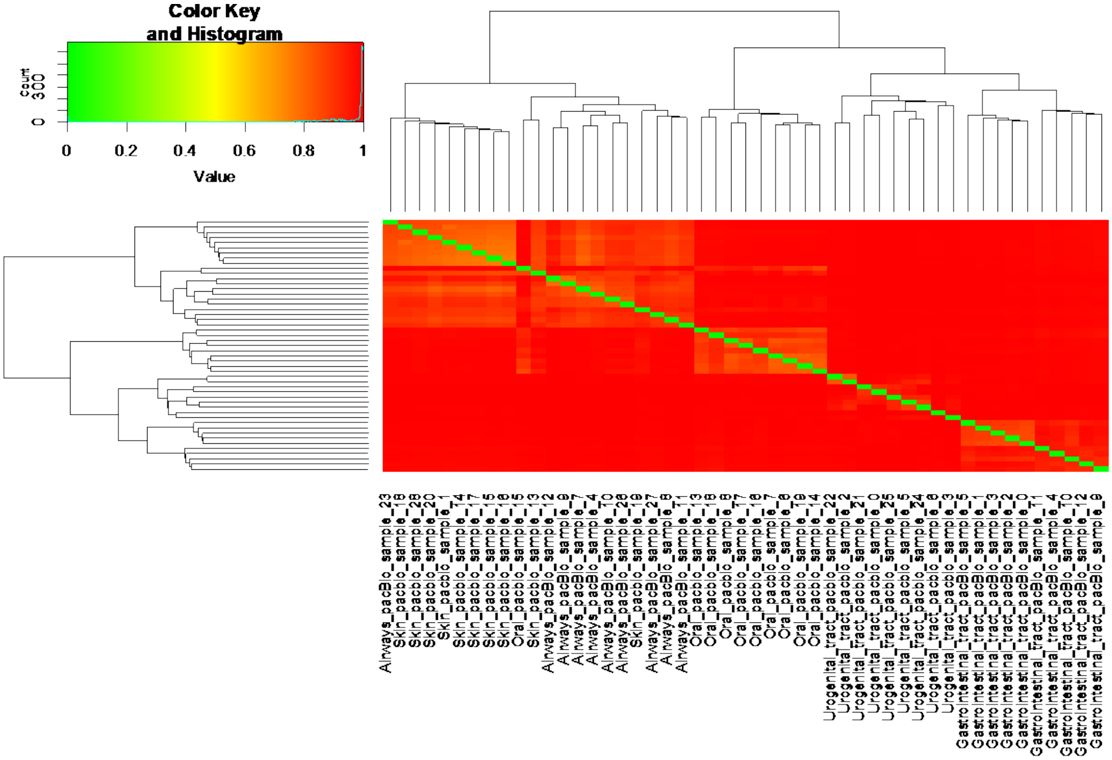

D

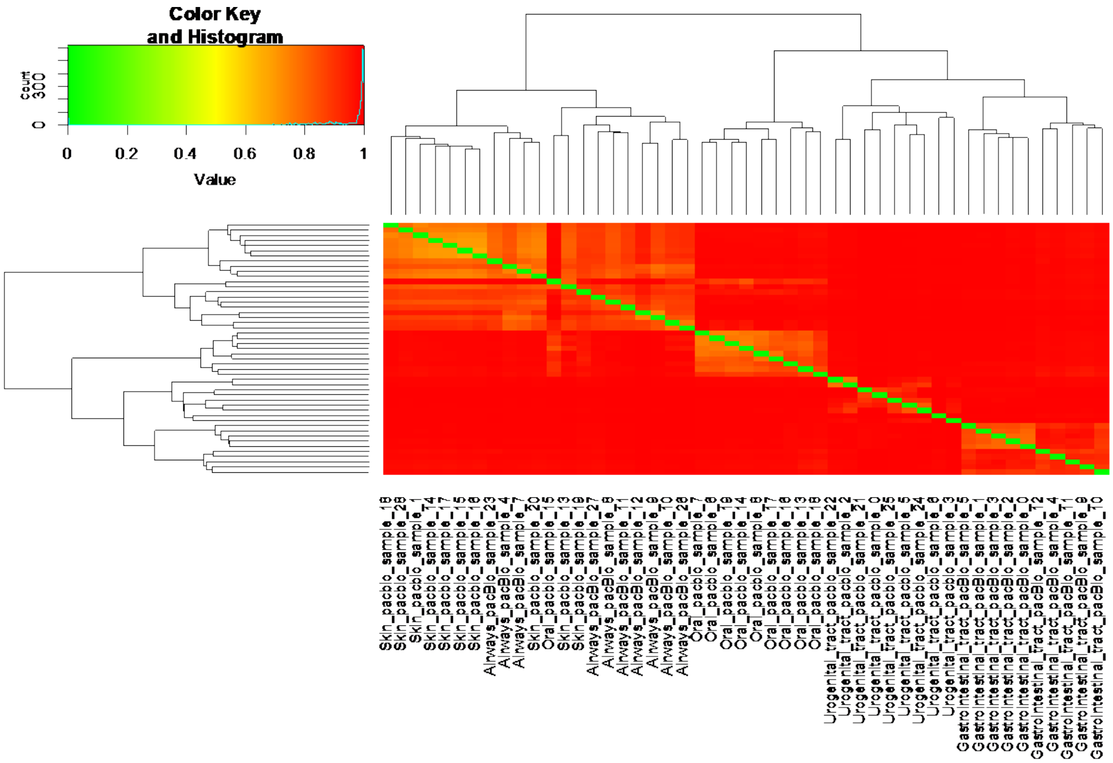

Supplemental Figure 2 : Comparison of Libra (Log), Mash and Simka (Jaccard and Bray-Curtis) for the analysis and clustering of the CAMI PacBio dataset

Sample to sample distance was computed on 48 HMP assemblies using Libra, Log weighting (a), Mash (b) or Simka, using Jaccard distance (c) and Bray-Curtis distance (d). The samples were clustered using Ward's method on their distance scores.
