# Supplementary material for: Libra: scalable k-mer–based tool for massive all-vs-all metagenome comparisons
Source: Gigascience. 2018 Dec 28;8(2):giy165. doi: 10.1093/gigascience/giy165 (PMC6354030; doi:10.1093/gigascience/giy165)
Supplement: Supplemental Files [file giy165_supplemental_files.zip › Supplemental Fig3.pdf]

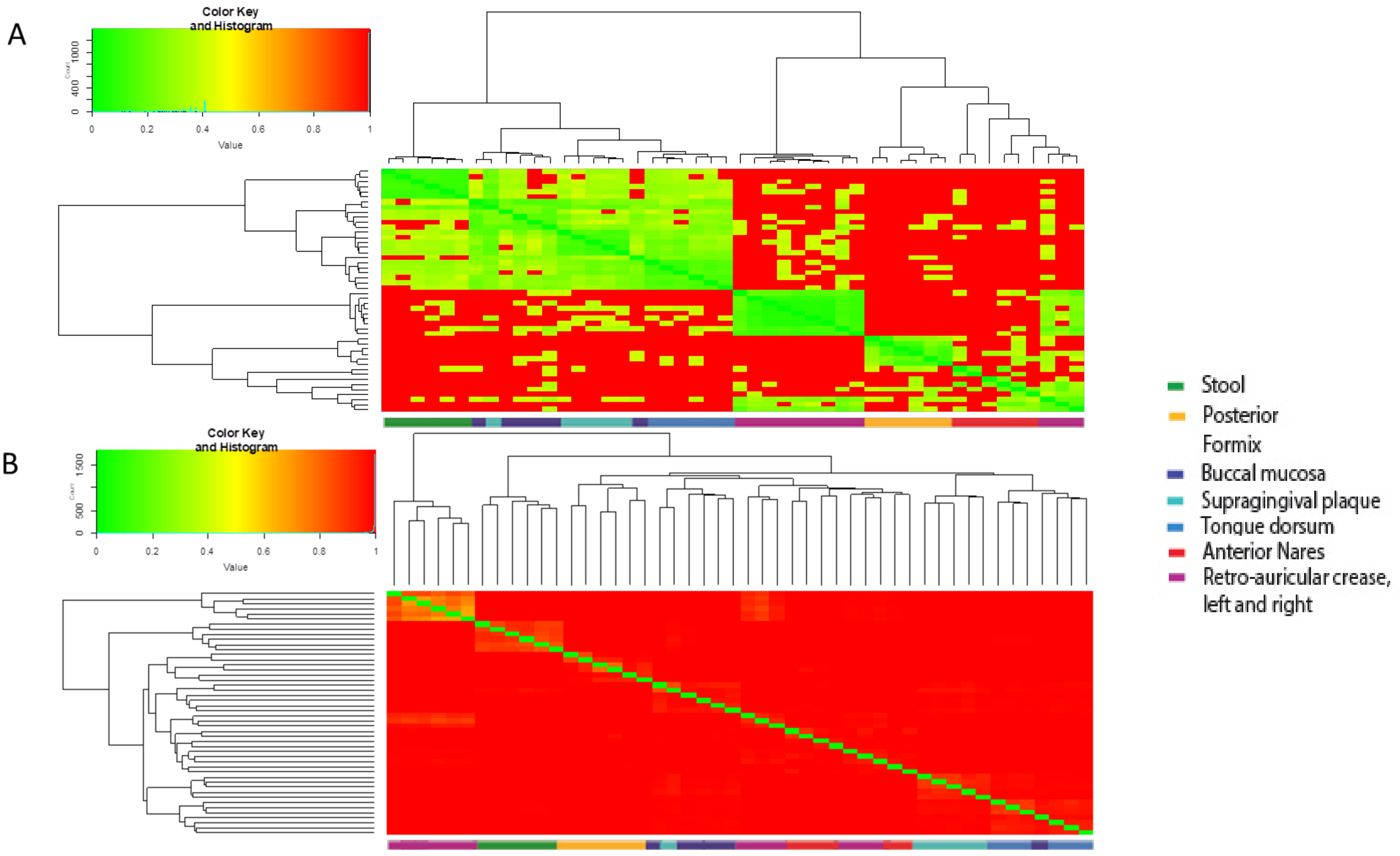

Supplemental Figure 3. Comparison of Mash and Libra for the analysis and clustering of HMP assemblies
Sample to sample distance was computed on 48 HMP assemblies using Mash (A) or Libra (B). The samples were clustered using Ward's method on their distance scores.
