# Supplementary material for: Libra: scalable k-mer–based tool for massive all-vs-all metagenome comparisons
Source: Gigascience. 2018 Dec 28;8(2):giy165. doi: 10.1093/gigascience/giy165 (PMC6354030; doi:10.1093/gigascience/giy165)
Supplement: Supplemental Files [file giy165_supplemental_files.zip › Supplemental Fig4.pdf]

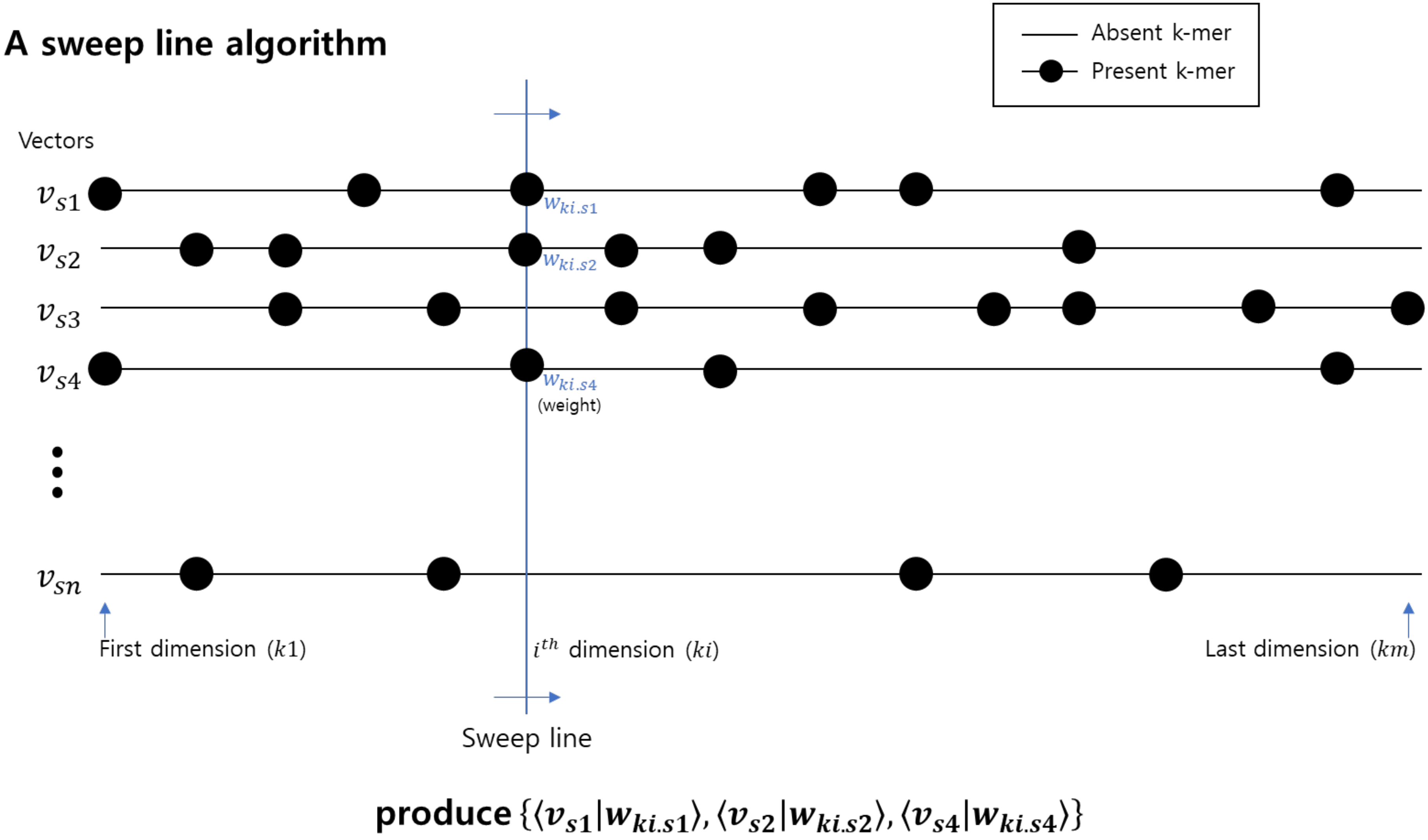

Supplemental Figure 4. Schema of the sweep line algorithm in distance matrix computation
A sweep line moves from the first dimension to the last (left to right). At every dimension containing k-mers (black dots), an output record is produced from the weights of the k-mers (based on k-mer abundance) on the sweep line.
