# Supplementary material for: Libra: scalable k-mer–based tool for massive all-vs-all metagenome comparisons
Source: Gigascience. 2018 Dec 28;8(2):giy165. doi: 10.1093/gigascience/giy165 (PMC6354030; doi:10.1093/gigascience/giy165)
Supplement: Supplemental Files [file giy165_supplemental_files.zip › Supplemental Fig5.pdf]

Partitioning the k-mer space

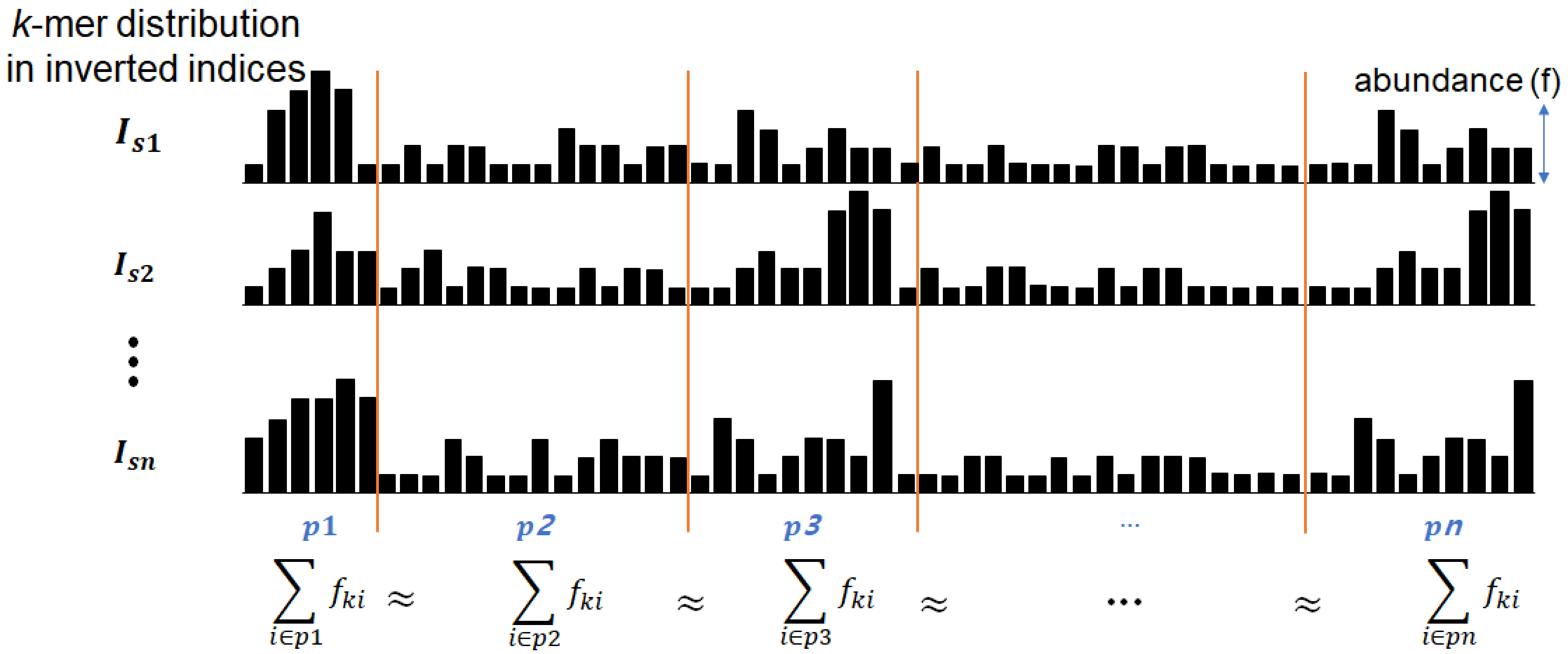

Supplemental Figure 5. Schema of the partitioning algorithm

Libra partitions the k-mer space based on the k-mer distribution to balance workloads across the partitions. Each partition has roughly the same number of records by having the same total k-mer counts.
