# Supplementary material for: Libra: scalable k-mer–based tool for massive all-vs-all metagenome comparisons
Source: Gigascience. 2018 Dec 28;8(2):giy165. doi: 10.1093/gigascience/giy165 (PMC6354030; doi:10.1093/gigascience/giy165)
Supplement: Supplemental Files [file giy165_supplemental_files.zip › supplemental_Fig1.pdf]

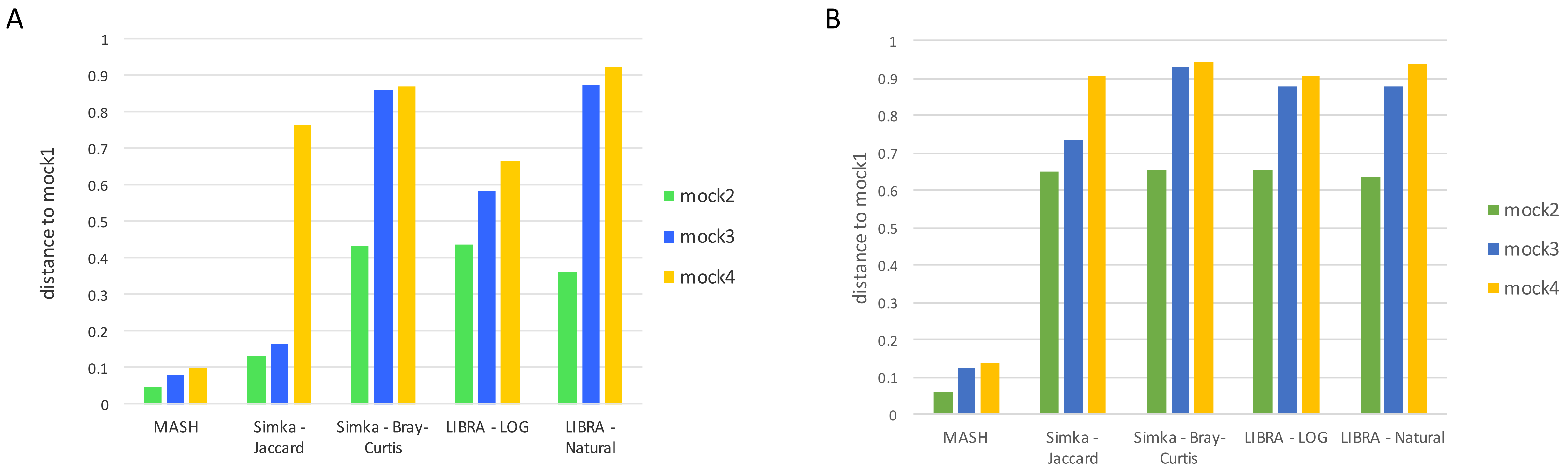

Supplemental Figure 4 : Analysis of simulated metagenomes using Mash, Simka and Libra.

Distance to staggered mock community artificial metagenome (mock 1), for simulated metagenomes from increasingly distant communities. The mock 1 relies on the known abundance profile from the staggered mock community. The mock 2 community profile was obtained by randomly inverting 3 species abundance from mock 1 profile. The mock 3 profile was obtained by randomly inverting 2 species abundances from mock 2 profile. Finally mock 4 profile was obtained by adding high abundance archeal genomes not present in any the other mock communities.

Simulated illumina metagenomes (A) were generated using GemSim at 100 million reads, and the PacBio simulated metagenomes (B) were generated using SIMLORD at 1 million reads. The distance between the mock 1 community to mock 2, mock 3, mock 4 was computed using Mash, SIMKA (Jaccard and Bray-curtis distance) and LIBRA (cosine distance, natural and logarithmic weighting).
