# Supplementary material for: Libra: scalable k-mer–based tool for massive all-vs-all metagenome comparisons
Source: Gigascience. 2018 Dec 28;8(2):giy165. doi: 10.1093/gigascience/giy165 (PMC6354030; doi:10.1093/gigascience/giy165)
Supplement: Response_to_Reviewer_Comments_Original_Submission.pdf [file giy165_response_to_reviewer_comments_original_submission.pdf]

Author's Response To Reviewer Comments

Close

As part of your revisions, it would be great if you can include performance evaluation in the case of long reads from Oxford Nanopore, PacBio, or Illumina sequencers. Reviewer #2 suggests to use some real nanopore datasets (available in e.g.,https://github.com/nanopore-wgs-consortium/NA12878/blob/master/Genome.md) for testing and evaluating Libra against other tools.

RESPONSE: We thank the reviewer and the editor for this excellent suggestion. We performed additional experiments using long read data (for the mock community and HMP datasets) per the reviewer's suggestion to evaluate Libra in comparison to other tools. The results show that Libra performs equally well on long and short read datasets. These data have been included in the manuscript, and as a detailed response to the reviewer below. We also go one step further, to show that Illumina and 454 short read technologies produce consistent results.

In addition, please register any new software application in the SciCrunch.org database to receive a RRID (Research Resource Identification Initiative ID) number, and include this in your manuscript. This will facilitate tracking, reproducibility and re-use of your tool.

RESPONSE: Thank you for the excellent recommendation. We have now registered Libra as a tool in SciCrunch.org and have added the RRID (SCR_016608) to the manuscript for tracking and re-use of our tool.

Response to Reviewers

Reviewer reports:

Reviewer #1: Title: Libra: robust biological inferences of global datasets using scalable k-mer based all-vs- all metagenome comparisons

Summary:

The authors present Libra, a software system for metagenomics sequence data analysis. Libra is "the first step in implementing a cloud-based resource." The authors claim 3 innovations: (1) Libra uses Hadoop, (2) Libra use of distance metrics, (3) Libra runs on CyVerse. The manuscript presents a software system that bundles known techniques into an integrated platform that should scale well to large datasets and is freely available on an existing cloud resource.

Commentary:

The software appears to be useful and well architected. The comparison to other tools is extensive. The manuscript says this was the first step of a system in development. The manuscript may be better presented as an application note or a progress report published elsewhere rather than a Research article for GigaScience. A paper with similar scope and similar format, published in GigaScience and referenced in this manuscript, appeared as a Review article not a Research article (Guo R, Zhao Y, Zou Q, Fang X, Peng S. Bioinformatics applications on Apache Spark. Gigascience. 2018).

RESPONSE: We sincerely thank the reviewer for understanding and recognizing the merit of the work. We decided to pursue a Research Article rather than a Data Note given that in addition to performing extensive analyses to compare and contrast the Libra to other tools based on synthetic data and mock communities, we also re-analyzed the Tara Oceans Virome data to reveal new biological insights that were missed in the original 2015 Science article. Specifically, we show for the first time that viral communities in the ocean are similar across temperature gradients, irrespective of their location in the ocean. We feel that this finding provides additional scientific insight into viruses in the ocean and therefore merits publication as a GigaScience Research article, rather than Data Note which would be constrained to just technical advances.

As a Research article, the manuscript makes three claims to innovation. One claimed innovation is Libra's use of sophisticated distance metrics. Libra gives users a choice of three metrics. The manuscript says two of those metrics are "widely used" and the other is "a new distance metric … using Cosine Similarity" (line 140). This is not the first use of cosine similarity in metagenomics (e.g., Virtual metagenome reconstruction from 16S rRNA gene sequences. Okuda et al. Nature Communications 2012). The manuscript does not distinguish this usage from prior ones. The authors say cosine similarity was demonstrated here only because it had the shortest runtime (line 235). The other two claims to innovation specify the use of Hadoop and CyVerse but both are widely used already. Thus, the claims seem unproven.

RESPONSE: We appreciate the reviewers' comments. Distance metrics have been widely used in metagenomics for a variety of purposes. In the paper the reviewer cites, cosine similarity was used as a metric to evaluate the accuracy of reconstructed genomes from "virtual metagenomes" based on the number of KEGG Orthologous Genes in common. The "virtual metagenomes" were derived based on species present in a 16S rRNA dataset obtained from gel electrophoresis (amplicon data), and are technically not from metagenomes which would consist of WGS data from microbes in a sample. Therefore the analysis is based on gene counts in genomes, and not on metagenomic sequence data. Our approach uses cosine similarity as a distance metric for comparing complete metagenomic sequence signatures, that has not been applied in this capacity before (in comparable tools Mash and Simka). As suggested, we updated the paper to cite this reference and describe its use in an alternative capacity in genome analysis. Similarly, no other tool for comparing sequence signatures from metagenomes uses Hadoop for massive analytics, or has been imbedded in the CyVerse cyberinfrastructure. Thus, these innovations remain novel for our use-case and stated applications.

Some claims would be easier to assess if the language were more precise. For example: (1) The Title claims the new tool provides robust inference and the Abstract claims that other tools

diminish the robustness of analysis. The manuscript also says Hadoop is robust. "Robust" is not defined or discussed further. (2) The Abstract describes Libra's three distance metrics as "complex" and the Innovations section refers to them as "sophisticated" but neither word gets defined or defended.

RESPONSE: We thank the reviewer for pointing out the need for further clarification of these terms. In the "Libra Implementation" section we define robust in the following way: "Hadoop allows robust parallel computation over distributed computing resources via its simple programming interface called MapReduce, while hiding much of the complexity of distributed computing (e.g. node failures)." The term robust refers to the ability to handle error without the need to restart analyses which is vital as the scale of data increases. We have updated the text to explicitly define this and have also removed the word "robust" from the title.
We define complex distance metrics in the introduction in the following way "simple distances scale linearly and complex distances metrics scale quadratically as additional samples are added". We define "complex distance" as a distance metric with a high complexity in terms of compute time. This is an important point, we have removed the term from the text to avoid confusion.

We agree with the reviewer that sophisticated is not a precise word choice and have removed the term from the Innovations section to be consistent with the abstract.

The referencing could use more rigor. For example: (1) Cosine similarity is introduced with an off-topic reference [34] (line 140) to a conference talk that compares several similarity metrics within the domain of document clustering. (2) A seemingly relevant review of prior art is not referenced (Web Resources for Metagenomics Studies. Dudhadara et al. GPB 2015). A seemingly relevant claim to prior art, found right in the CyVerse online documentation, is not noted (Scalable metagenomic analysis using iPlant. Vaughn. CyVerse Wiki 2013). (3) The Introduction says one existing tool is the fastest (line 72) without reference or explanation. The same paragraph states that abundance is a critical and previously ignored factor "central to microbial ecology" without providing a reference or sufficient evidence.

RESPONSE: Thank you for your careful review and drawing our attention to issues with the references. We have carefully reviewed the references and updated according to the reviewer's suggestions. We removed the reference for cosine similarity given that other publications in the field do not reference any papers, given that it is a commonly used similarity metric.

Reviewer #2: The authors developed a new k-mer based method called Libra that enables large scaled metagenomics samples comparison. The authors introduced the advanced method MapReduce to the area of comparative metagenomics and designed a pipeline for counting k-mers and computing distances using MapReduce. The new method was extensively evaluated on simulations and real datasets. The authors also made the software available on iMicrobe, which is easily accessible by biologists in the community. Overall the manuscript is well written and the datasets are publicly available. More details and discussions can be added in order to make the paper more comprehensive. Here are some comments:

RESPONSE: We thank the reviewer for recognizing the value of the work and providing

valuable suggestions for enhancing the work.

1. In Figure 2A, it seems that the distances defined by Mash and Libra decrease as the sequencing depth increases. However, the authors claim that "sequencing depth has little effect on the distance between samples in Mash and Libra (natural weighting)", which is confusing. Ideally, since the four artificial metagenomes were generated from the same community as the original sample, the distance between the artificial sample and the original sample should be small. The figure shows that as sample size is as large as 5M, the distance of Libra is close to 0. The large distance for small sample size may due to the variation in the sampling. The authors could elaborate more on the results.

RESPONSE: If the communities were sampled at their exact ratios we would theoretically get a distance of zero irrespective of the sample size. However, similar to real-world sequencing, random sampling selects more sequences from dominant organisms than rare (based on a higher probability of sampling a dominant organism over a rare one). This means that decreasing the sequencing depth removes the rare community component. Simka does not see this effect, because they normalize all samples to the lowest read count. Whereas Mash and Libra are taking into account all of the reads in the metagenomes, therefore they measure a larger difference when you compare the smallest (0.5M read sample) and largest (10 million read sample). We have updated the text to better describe this important point.

2. The authors claimed that "the Mash algorithm shows lower overall resolution (Figure 3A) as compared to Libra (Figure 3B)". Could the authors explain more how they defined "resolution"? From Figure 2B, it seems to me that the range of Mash distance is relatively smaller compared with that of other measures. So plotting heatmaps under the same range (0-1) may lead to the unclear patterns for Mash as what we see in Figure 3A.

RESPONSE: Thank you for your comment. This is indeed an important clarification. Mash, Simka, and Libra all report distance in the same range (0-1), and therefore we plot the data according to the reported results from each tool. The distance between metagenomes that Mash is able to detect based on the sketching algorithm (that uses a subset of reads) is small, leading to lower resolution in the graph compared to Simka and Libra that use 100% of the reads. We have updated the legend for the Figure to better describe this important point.

3. The author claimed from Figure 4 that "these differences reflect the effect of using all of the read data (Libra) rather than a subset (Mash)." It is true that Mash estimate the distance based on a subset of data. On the other hand, Mash and Libra use different measures. So the difference in clustering may also come from the different measures. The authors could add a discussion for this.

RESPONSE: We agree with the reviewer’s comment. Distance metrics are fundamental to comparative metagenomic analyses, but also add clarification on the importance of using abundance in the distance calculation. In Figure 4, Mash (Fig 4A) and Simka (Fig 4C) both use Jaccard distance, however Simka achieves better clustering by using all of the reads and including the abundance in the distance calculation. We have updated the text to clarify this point and also reference the Simka paper which shows a careful analysis of the effects of

sketching compared to using all of the k-mers.

4. Have the authors compared the running time of Libra with other methods? It would be great to see if Libra can have high accuracy and at the meanwhile reduces the running time or is within the similar running time with other methods.

RESPONSE: A direct comparison of the runtime of the tools is not possible given that each tool runs on a different computational architecture with a different number of servers and total CPU/memory (Mash runs on a single server; Simka runs on an HPC; and Libra on Hadoop). When running the HMP dataset we found that Mash runs in minutes, Simka in 2-3 hours, and Libra in ~12 hours. Because Libra uses a Hadoop framework, staging the data into HDFS takes significant run time, although the calculations are fast. Libra is developed as a method to scale to large datasets and be fault tolerant, whereas smaller datasets will run faster and with equal resolution using Simka. Thus, the major innovation Libra provides is analyses at scale. This important point was added into the discussion.

Reviewer #3: Choi et al propose a new tool called Libra for computing pairwise comparisons of samples in the case of large set of samples that is scalable (via cloud-based resources), fast and as accurate as (or better) than standard methods.
Several major and minor issues were detected:

RESPONSE: We thank the reviewer for their time and excellent suggestions.

Major issues:
- Unlike authors of Mash, authors of Libra do not provide any performance evaluation in case of long reads from Oxford Nanopore, PacBio, or Illumina sequencers. It seems Libra was only tested for short reads.
If this is the case and given the fact that long reads (10kbp or more) are becoming standard size for metagenomics, genomics (cf. numerous paper published in Nature methods, and Nature Biotechnology dealing with Nanopore reads) then authors should explicitly mention in the manuscript as well as in the title of the manuscript that Libra works only for short reads.
Otherwise, if Libra can be used for Nanopore sequencing for example then authors should create synthetic datasets with NanoSim (Yang et al, GigaScience. 2017.doi:10.1093/gigascience/gix010) and show the performance of it.

Also several real datasets of nanopore data are available (e.g., https://github.com/nanopore-wgs-consortium/NA12878/blob/master/Genome.md) for testing and should be used for evaluating Libra against the other tools.

RESPONSE: We thank the reviewer for their excellent and timely suggestion, we have added new experiments that demonstrate the utility of each of the tools (Mash, Simka, and Libra) on long read data. Specifically, we show that simulated data long read data for the mock community shows a similar stepwise distance pattern between each of the mock communities (as expected), but has a higher overall distance between each of the mock communities likely due to the high simulated random error rate compared to short read data. We added this analysis to the results, and included a new supplemental figure to show the results. Thus, all of the tools

can distinguish differences in long read and short read data alike. Please note that we chose to use SimLoRD for the simulated metagenomic data given that Nanosim is constrained to simulated genomic data. The same supplemental also includes the simulated data for the mock community based on Illumina data (per the reviewer's suggestion below).

Per the reviewer's suggestion, we have also added an analysis of the CAMI HMP "toy dataset" with simulated long reads from PacBio, to complement the analyses we already ran on real short read Illumina data from the Human Microbiome Project. This analysis shows that each of the tools is able to cluster the samples broadly by body site, however there are small misclassifications shared across all tools. These data suggest that increased error rate of the technology could have a limited impact on k-mer based analytics.

- The supplemental document, in docx format, containing information about methods has formulas that are not readable. Please correct and update this document, compile it in PDF, and also include as much as possible of it in the main text.

RESPONSE: We thank the reviewer for drawing our attention to this issue. We integrated the supplemental methods document into a comprehensive and refined methods section in the main article. All formulas have been checked and fixed.

Minor issues:
- Please provide a reference related to the microbial dark matter in for the claim in introduction:"k-mer based classifiers that rapidly assign metagenomic reads to known microbes miss the microbial dark matter". Then, please discuss/explain how well/bad is Libra to deal with "the microbial dark matter" that these taxonomic classifiers miss?

RESPONSE: Thank you for pointing out the missing reference, we have updated the text to include a reference. A detailed discussion of how comparative metagenomic approaches in general (employed by Mash, Libra, and Simka) elucidate the unknown fraction of metagenomes is included in the section titled "De novo comparative metagenomics offers a path forward."

- Table 1: This big table provides a long list of tools and yet the list is not exhaustive. Since this list is not exhaustive, and it is not clear how the tools were selected or even ordered, I'd recommend to explain better or put in supplement.
I'd also include a recent paper surveying these tools of your choice in case the readers want to know more and to simplify the reading.

RESPONSE: Thank you for this suggestion. The main point of the table was to show that tools have been developed to compare genomes using Hadoop (which are much smaller in terms of total bytes), but none compare metagenomes to-date. Moreover, none of these Hadoop-based tools are not available in an easy to use web-interface and accessible to the general user. We also show that metagenomic tools extensively use k-mer based analytics, most of these perform comparisons to known reference databases for taxonomic classification, and some have been developed to compare reads between metagenomes (however most cannot scale). We also point out that there are a number of tools for k-mer based comparisons, but none of these calculate the distance between metagenomes. We agree with the reviewer and have moved the table to

supplemental.

- For Figure 2, authors created "synthetic" or "simulated" datasets and called them "artificial". Why? Authors should rather call these datasets "synthetic" or "simulated" to be consistent with the language used by authors of GemSIM and generally language used in studies using synthetic datasets built with known profile.

RESPONSE: Thank you for pointing this out, we have updated the figures, figure legends, and text throughout the manuscript to consistently using the word “simulated”.

- Authors do show tests with 454 reads, however since this technology is not supported any more, I am afraid this evaluation brings limited value.

RESPONSE: We agree with the reviewer that 454 technology is not used as often these days, but have chosen to include 454 in addition to Illumina/Pacbio data (added in Supplemental Figure X) for the mock community analysis to show that the methodology works irrespective of the sequencing platform. This point is important for users who wish to compare new datasets with older datasets derived from 454 technologies.

- Please detail what are all the parameters for Libra's settings (for example, is the k-mer length variable ? is k equal to 21 like MASH's index ?...).

RESPONSE: We thank the reviewer for pointing this out. We have updated the methods to include information about the k-mer size and settings for Libra.

Close
