# Supplementary material for: Libra: scalable k-mer–based tool for massive all-vs-all metagenome comparisons
Source: Gigascience. 2018 Dec 28;8(2):giy165. doi: 10.1093/gigascience/giy165 (PMC6354030; doi:10.1093/gigascience/giy165)
Supplement: Reviewer_1_Report_Original_Submission -- Jason R. Miller, MS — 9/5/2018 Reviewed [file giy165_reviewer_1_report_original_submission.pdf]

Reviewer Report

Title: Libra: scalable k-mer based tool for massive all-vs-all metagenome comparisons

Version: Original Submission **Date:** 9/5/2018

Reviewer name: Jason R. Miller, MS

Reviewer Comments to Author:

Title: Libra: robust biological inferences of global datasets using scalable k-mer based all-vs- all metagenome comparisons

Summary:

The authors present Libra, a software system for metagenomics sequence data analysis. Libra is "the first step in implementing a cloud-based resource." The authors claim 3 innovations: (1) Libra uses Hadoop, (2) Libra use of distance metrics, (3) Libra runs on CyVerse. The manuscript presents a software system that bundles known techniques into an integrated platform that should scale well to large datasets and is freely available on an existing cloud resource.

Commentary:

The software appears to be useful and well architected. The comparison to other tools is extensive. The manuscript says this was the first step of a system in development. The manuscript may be better presented as an application note or a progress report published elsewhere rather than a Research article for GigaScience. A paper with similar scope and similar format, published in GigaScience and referenced in this manuscript, appeared as a Review article not a Research article (Guo R, Zhao Y, Zou Q, Fang X, Peng S. Bioinformatics applications on Apache Spark. Gigascience. 2018).

As a Research article, the manuscript makes three claims to innovation. One claimed innovation is Libra's use of sophisticated distance metrics. Libra gives users a choice of three metrics. The manuscript says two of those metrics are "widely used" and the other is "a new distance metric … using Cosine Similarity" (line 140). This is not the first use of cosine similarity in metagenomics (e.g., Virtual metagenome reconstruction from 16S rRNA gene sequences. Okuda et al. Nature Communications 2012). The manuscript does not distinguish this usage from prior ones. The authors say cosine similarity was demonstrated here only because it had the shortest runtime (line 235). The other two claims to innovation specify the use of Hadoop and CyVerse but both are widely used already. Thus, the claims seem unproven.

Some claims would be easier to assess if the language were more precise. For example: (1) The Title claims the new tool provides robust inference and the Abstract claims that other tools diminish the robustness of analysis. The manuscript also says Hadoop is robust. "Robust" is not defined or discussed

further. (2) The Abstract describes Libra's three distance metrics as "complex" and the Innovations section refers to them as "sophisticated" but neither word gets defined or defended.

The referencing could use more rigor. For example: (1) Cosine similarity is introduced with an off-topic reference [34] (line 140) to a conference talk that compares several similarity metrics within the domain of document clustering. (2) A seemingly relevant review of prior art is not referenced (Web Resources for Metagenomics Studies. Dudhadara et al. GPB 2015). A seemingly relevant claim to prior art, found right in the CyVerse online documentation, is not noted (Scalable metagenomic analysis using iPlant. Vaughn. CyVerse Wiki 2013). (3) The Introduction says one existing tool is the fastest (line 72) without reference or explanation. The same paragraph states that abundance is a critical and previously ignored factor "central to microbial ecology" without providing a reference or sufficient evidence.

Level of Interest

Please indicate how interesting you found the manuscript: Choose an item.

Quality of Written English

Please indicate the quality of language in the manuscript: Choose an item.

Declaration of Competing Interests

Please complete a declaration of competing interests, considering the following questions:

- Have you in the past five years received reimbursements, fees, funding, or salary from an organisation that may in any way gain or lose financially from the publication of this manuscript, either now or in the future?
- Do you hold any stocks or shares in an organisation that may in any way gain or lose financially from the publication of this manuscript, either now or in the future?
- Do you hold or are you currently applying for any patents relating to the content of the manuscript?
- Have you received reimbursements, fees, funding, or salary from an organization that holds or has applied for patents relating to the content of the manuscript?
- Do you have any other financial competing interests?
- Do you have any non-financial competing interests in relation to this paper?

If you can answer no to all of the above, write 'I declare that I have no competing interests' below. If your reply is yes to any, please give details below.

I declare that I have no competing interests

I agree to the open peer review policy of the journal. I understand that my name will be included on my report to the authors and, if the manuscript is accepted for publication, my named report including any attachments I upload will be posted on the website along with the authors' responses. I agree for my report to be made available under an Open Access Creative Commons CC-BY license (http://creativecommons.org/licenses/by/4.0/). I understand that any comments which I do not wish to be included in my named report can be included as confidential comments to the editors, which will not be published.

I agree to the open peer review policy of the journal

To further support our reviewers, we have joined with Publons, where you can gain additional credit to further highlight your hard work (see: https://publons.com/journal/530/gigascience). On publication of this paper, your review will be automatically added to Publons, you can then choose whether or not to claim your Publons credit. I understand this statement.

Yes Choose an item.
