# Supplementary material for: Libra: scalable k-mer–based tool for massive all-vs-all metagenome comparisons
Source: Gigascience. 2018 Dec 28;8(2):giy165. doi: 10.1093/gigascience/giy165 (PMC6354030; doi:10.1093/gigascience/giy165)
Supplement: Reviewer_1_Report_Revision_1 -- Jason R. Miller, MS — 11/1/2018 Reviewed [file giy165_reviewer_1_report_revision_1.pdf]

Reviewer Report

Title: Libra: scalable k-mer based tool for massive all-vs-all metagenome comparisons

Version: Revision 1 **Date:** 11/1/2018

Reviewer name: Jason R. Miller, MS

Reviewer Comments to Author:

Repeating my original observations, Libra appears to be useful and well architected. An extensive comparison to other tools is presented. I appreciate that the authors made specific revisions to the text. However, I feel my most important suggestions were not addressed.

My main suggestion was that this would be better presented as an Application Note, possibly in a different journal. In their response to reviewers, and in defense of submitting a GigaScience Research Article, the authors pointed to their finding that viral communities in the Tara ocean data are similar across temperature gradients, saying this fact was missed in the earlier Tara publication and is being reported here for the first time. If this were the critical finding, then I'd expect it to appear prominently. In fact, it is mentioned twice. First, "Taken together, these data indicate that viral populations are structured globally by temperature, and at finer resolution by station (for surface and DCM samples) indicating that micronutrients and local conditions play an important role in defining viral populations." Second, "We show for the first time that viral communities in the ocean are similar across temperature gradients, irrespective of their location in the ocean." This treatment does not point out any contradiction to the previous study. The finding is not mentioned in the heading of the subsection, the caption of Table 1 about Tara run time, or the caption of Figure 5 about Tara results. The finding is not mentioned in the Title or in the Abstract or in the Innovations section. The finding appears to be based on a visual interpretation that is vague ("largely structured by temperature") and provided without statistics. Thus, the wording of the manuscript suggests that this finding was presented, not as a conclusion about the oceans, but as an example of how Libra can be used. In its guide for authors, GigaScience says, "Research Articles present work utilising large scale data that provide some scientific insight and conclusions" (https://academic.oup.com/gigascience//pages/research). With respect, I maintain that the revised manuscript is an Application Note and not a Research Article.

Secondly, I had noted that the manuscript makes 3 claims to innovation with insufficient support. In their response to reviewers, the authors added the qualification that their application of Hadoop was a first in metagenomics. However, the revised manuscript omits that qualification. After saying, "Libra presents three main innovations", the revised text claims (1) "the use of a scalable Hadoop framework enabling massive dataset comparison" is novel. This sentence does not include any first-in-metagenomics qualification. The claim is unsupported as written. The revised text claims (2) "linear calculations for complex distance metrics allowing for high accuracy and clustering of the metagenomes based on their k-mer content" is novel. This sentence combines 6 ideas, leaving it unclear what precisely is being claimed. Is this the first linear-time calculation, or the first highly-accurate calculation, or the

first k-mer based calculation, or some combination? I find this claim unsupportable as written. The revised text claims (3) "a web-based tool imbedded in the CyVerse advanced cyberinfrastructure through iMicrobe for broader use of the tool in the scientific community" is novel. This claim has no first-in-metagenomics qualification. The claim is unsupported as written. With respect, I maintain that the revised manuscript's three claims to innovation are unproven.

A more thorough review might have been possible had Tracked Changes been presented.

Level of Interest

Please indicate how interesting you found the manuscript: Choose an item.

Quality of Written English

Please indicate the quality of language in the manuscript: Choose an item.

Declaration of Competing Interests

Please complete a declaration of competing interests, considering the following questions:

- Have you in the past five years received reimbursements, fees, funding, or salary from an organisation that may in any way gain or lose financially from the publication of this manuscript, either now or in the future?
- Do you hold any stocks or shares in an organisation that may in any way gain or lose financially from the publication of this manuscript, either now or in the future?
- Do you hold or are you currently applying for any patents relating to the content of the manuscript?
- Have you received reimbursements, fees, funding, or salary from an organization that holds or has applied for patents relating to the content of the manuscript?
- Do you have any other financial competing interests?
- Do you have any non-financial competing interests in relation to this paper?

If you can answer no to all of the above, write 'I declare that I have no competing interests' below. If your reply is yes to any, please give details below.

I declare that I have no competing interests

I agree to the open peer review policy of the journal. I understand that my name will be included on my report to the authors and, if the manuscript is accepted for publication, my named report including any attachments I upload will be posted on the website along with the authors' responses. I agree for my report to be made available under an Open Access Creative Commons CC-BY license (http://creativecommons.org/licenses/by/4.0/). I understand that any comments which I do not wish to

be included in my named report can be included as confidential comments to the editors, which will not be published.

I agree to the open peer review policy of the journal

To further support our reviewers, we have joined with Publons, where you can gain additional credit to further highlight your hard work (see: https://publons.com/journal/530/gigascience). On publication of this paper, your review will be automatically added to Publons, you can then choose whether or not to claim your Publons credit. I understand this statement.

Yes Choose an item.
