# Supplementary material for: Libra: scalable k-mer–based tool for massive all-vs-all metagenome comparisons
Source: Gigascience. 2018 Dec 28;8(2):giy165. doi: 10.1093/gigascience/giy165 (PMC6354030; doi:10.1093/gigascience/giy165)
Supplement: Reviewer_2_Report_Original_Submission -- Jie Ren — 9/12/2018 Reviewed [file giy165_reviewer_2_report_original_submission.pdf]

Reviewer Report

Title: Libra: scalable k-mer based tool for massive all-vs-all metagenome comparisons

Version: Original Submission **Date:** 9/12/2018

Reviewer name: Jie Ren

Reviewer Comments to Author:

The authors developed a new k-mer based method called Libra that enables large scaled metagenomics samples comparison. The authors introduced the advanced method MapReduce to the area of comparative metagenomics and designed a pipeline for counting k-mers and computing distances using MapReduce. The new method was extensively evaluated on simulations and real datasets. The authors also made the software available on iMicrobe, which is easily accessible by biologists in the community. Overall the manuscript is well written and the datasets are publicly available. More details and discussions can be added in order to make the paper more comprehensive. Here are some comments:

1. In Figure 2A, it seems that the distances defined by Mash and Libra decrease as the sequencing depth increases. However, the authors claim that "sequencing depth has little effect on the distance between samples in Mash and Libra (natural weighting)", which is confusing. Ideally, since the four artificial metagenomes were generated from the same community as the original sample, the distance between the artificial sample and the original sample should be small. The figure shows that as sample size is as large as 5M, the distance of Libra is close to 0. The large distance for small sample size may due to the variation in the sampling. The authors could elaborate more on the results.

2. The authors claimed that "the Mash algorithm shows lower overall resolution (Figure 3A) as compared to Libra (Figure 3B)". Could the authors explain more how they defined "resolution"? From Figure 2B, it seems to me that the range of Mash distance is relatively smaller compared with that of other measures. So plotting heatmaps under the same range (0-1) may lead to the unclear patterns for Mash as what we see in Figure 3A.

3. The author claimed from Figure 4 that "these differences reflect the effect of using all of the read data (Libra) rather than a subset (Mash)." It is true that Mash estimate the distance based on a subset of data. On the other hand, Mash and Libra use different measures. So the difference in clustering may also come from the different measures. The authors could add a discussion for this.

4. Have the authors compared the running time of Libra with other methods? It would be great to see if Libra can have high accuracy and at the meanwhile reduces the running time or is within the similar running time with other methods.

Level of Interest

Please indicate how interesting you found the manuscript: Choose an item.

Quality of Written English

Please indicate the quality of language in the manuscript: Choose an item.

Declaration of Competing Interests

Please complete a declaration of competing interests, considering the following questions:

- Have you in the past five years received reimbursements, fees, funding, or salary from an organisation that may in any way gain or lose financially from the publication of this manuscript, either now or in the future?
- Do you hold any stocks or shares in an organisation that may in any way gain or lose financially from the publication of this manuscript, either now or in the future?
- Do you hold or are you currently applying for any patents relating to the content of the manuscript?
- Have you received reimbursements, fees, funding, or salary from an organization that holds or has applied for patents relating to the content of the manuscript?
- Do you have any other financial competing interests?
- Do you have any non-financial competing interests in relation to this paper?

If you can answer no to all of the above, write 'I declare that I have no competing interests' below. If your reply is yes to any, please give details below.

I declare that I have no competing interests.

I agree to the open peer review policy of the journal. I understand that my name will be included on my report to the authors and, if the manuscript is accepted for publication, my named report including any attachments I upload will be posted on the website along with the authors' responses. I agree for my report to be made available under an Open Access Creative Commons CC-BY license (http://creativecommons.org/licenses/by/4.0/). I understand that any comments which I do not wish to be included in my named report can be included as confidential comments to the editors, which will not be published.

I agree to the open peer review policy of the journal

To further support our reviewers, we have joined with Publons, where you can gain additional credit to further highlight your hard work (see: https://publons.com/journal/530/gigascience). On publication of this paper, your review will be automatically added to Publons, you can then choose whether or not to claim your Publons credit. I understand this statement.

Yes Choose an item.
