# Supplementary material for: Libra: scalable k-mer–based tool for massive all-vs-all metagenome comparisons
Source: Gigascience. 2018 Dec 28;8(2):giy165. doi: 10.1093/gigascience/giy165 (PMC6354030; doi:10.1093/gigascience/giy165)
Supplement: Reviewer_3_Report_Original_Submission -- Rachid Ounit — 9/17/2018 Reviewed [file giy165_reviewer_3_report_original_submission.pdf]

Reviewer Report

Title: Libra: scalable k-mer based tool for massive all-vs-all metagenome comparisons

Version: Original Submission **Date:** 9/17/2018

Reviewer name: Rachid Ounit

Reviewer Comments to Author:

Choi et al propose a new tool called Libra for computing pairwise comparisons of samples in the case of large set of samples that is scalable (via cloud-based resources),
fast and as accurate as (or better) than standard methods.
Several major and minor issues were detected:

Major issues:
- Unlike authors of Mash, authors of Libra do not provide any performance evaluation in case of long reads from Oxford Nanopore, PacBio, or Illumina sequencers. It seems Libra was only tested for short reads.
If this is the case and given the fact that long reads (10kbp or more) are becoming standard size for metagenomics, genomics (cf. numerous paper published in Nature methods, and Nature Biotechnology dealing with Nanopore reads) then authors should explicitly mention in the manuscript as well as in the title of the manuscript that Libra works only for short reads.
Otherwise, if Libra can be used for Nanopore sequencing for example then authors should create synthetic datasets with NanoSim (Yang et al, GigaScience. 2017.doi:10.1093/gigascience/gix010) and show the performance of it.

Also several real datasets of nanopore data are available (e.g., https://github.com/nanopore-wgs-consortium/NA12878/blob/master/Genome.md) for testing and should be used for evaluating Libra against the other tools.

- The supplemental document, in docx format, containing information about methods has formulas that are not readable. Please correct and update this document, compile it in PDF, and also include as much as possible of it in the main text.

Minor issues:
- Please provide a reference related to the microbial dark matter in for the claim in introduction:"k-mer based classifiers that rapidly assign metagenomic reads to known microbes
miss the microbial dark matter". Then, please discuss/explain how well/bad is Libra to deal with "the microbial dark matter" that these taxonomic classifiers miss?

- Table 1: This big table provides a long list of tools and yet the list is not exhaustive. Since this list is not exhaustive, and it is not clear how the tools were selected or even ordered, I'd recommend to explain

better or put in supplement.
I'd also include a recent paper surveying these tools of your choice in case the readers want to know more and to simplify the reading.

- For Figure 2, authors created "synthetic" or "simulated" datasets and called them "artificial". Why? Authors should rather call these datasets "synthetic" or "simulated" to be consistent with the language used by authors of GemSIM and generally language used in studies using synthetic datasets built with known profile.

- Authors do show tests with 454 reads, however since this technology is not supported any more, I am afraid this evaluation brings limited value.

- Please detail what are all the parameters for Libra's settings (for example, is the k-mer length variable ? is k equal to 21 like MASH's index ?...).

Level of Interest

Please indicate how interesting you found the manuscript: Choose an item.

Quality of Written English

Please indicate the quality of language in the manuscript: Choose an item.

Declaration of Competing Interests

Please complete a declaration of competing interests, considering the following questions:

- Have you in the past five years received reimbursements, fees, funding, or salary from an organisation that may in any way gain or lose financially from the publication of this manuscript, either now or in the future?
- Do you hold any stocks or shares in an organisation that may in any way gain or lose financially from the publication of this manuscript, either now or in the future?
- Do you hold or are you currently applying for any patents relating to the content of the manuscript?
- Have you received reimbursements, fees, funding, or salary from an organization that holds or has applied for patents relating to the content of the manuscript?
- Do you have any other financial competing interests?
- Do you have any non-financial competing interests in relation to this paper?

If you can answer no to all of the above, write 'I declare that I have no competing interests' below. If your reply is yes to any, please give details below.

1. No 2. No 3. No 4. No 5. No

I agree to the open peer review policy of the journal. I understand that my name will be included on my report to the authors and, if the manuscript is accepted for publication, my named report including any attachments I upload will be posted on the website along with the authors' responses. I agree for my report to be made available under an Open Access Creative Commons CC-BY license (http://creativecommons.org/licenses/by/4.0/). I understand that any comments which I do not wish to be included in my named report can be included as confidential comments to the editors, which will not be published.

I agree to the open peer review policy of the journal

To further support our reviewers, we have joined with Publons, where you can gain additional credit to further highlight your hard work (see: https://publons.com/journal/530/gigascience). On publication of this paper, your review will be automatically added to Publons, you can then choose whether or not to claim your Publons credit. I understand this statement.

Yes Choose an item.
