# Supplementary material for: Libra: scalable k-mer–based tool for massive all-vs-all metagenome comparisons
Source: Gigascience. 2018 Dec 28;8(2):giy165. doi: 10.1093/gigascience/giy165 (PMC6354030; doi:10.1093/gigascience/giy165)
Supplement: Supplemental Files [file giy165_supplemental_files.zip › Response_to_reviews_round2.docx]

GIGA-D-18-00324R1

Libra: scalable k-mer based tool for massive all-vs-all metagenome comparisons

Illyoung Choi, MS; Alise J. Ponsero, PhD; Matthew Bomhoff, BS; Ken Youens-Clark, BA; John H. Hartman, PhD; Bonnie L Hurwitz, PhD

GigaScience

Dear Prof. Hurwitz,

Your manuscript "Libra: scalable k-mer based tool for massive all-vs-all metagenome comparisons" (GIGA-D-18-00324R1) has been assessed by our reviewers. Based on these reports, and my own assessment as Editor, I am pleased to inform you that it is potentially acceptable for publication in GigaScience, once you have carried out some essential revisions suggested by our reviewers.

Reviewer #1 requires a few more clarifications to be made. Their reports, together with any other comments, are below. Please also take a moment to check our website at <https://giga.editorialmanager.com/> for any additional comments that were saved as attachments.

*RESPONSE: We thank the reviewers for these important additional comments and clarifications. We agree with the reviewers and have addressed the comments in the manuscript per their recommendations. A point-by-point response is provided below. We also ask the the editor consider our resubmission for an Application Note and not a Research Article per reviewer 1’s comments below.*

In addition, please register any new software application in the SciCrunch.org database to receive a RRID (Research Resource Identification Initiative ID) number, and include this in your manuscript. This will facilitate tracking, reproducibility and re-use of your tool.

*RESPONSE: Thank you. We have registered Libra as a tool in SciCrunch.org and have added the RRID (SCR_016608) to the manuscript for tracking and re-use of our tool.*

The due date for submitting the revised version of your article is 06 Feb 2019.

We look forward to receiving your revised manuscript soon.

Best wishes,

Nicole Nogoy, Ph.D

GigaScience

www.gigasciencejournal.com

Reviewer reports:

Reviewer #1: Repeating my original observations, Libra appears to be useful and well architected. An extensive comparison to other tools is presented. I appreciate that the authors made specific revisions to the text. However, I feel my most important suggestions were not addressed.

My main suggestion was that this would be better presented as an Application Note, possibly in a different journal. In their response to reviewers, and in defense of submitting a GigaScience Research Article, the authors pointed to their finding that viral communities in the Tara ocean data are similar across temperature gradients, saying this fact was missed in the earlier Tara publication and is being reported here for the first time. If this were the critical finding, then I'd expect it to appear prominently. In fact, it is mentioned twice. First, "Taken together, these data indicate that viral populations are structured globally by temperature, and at finer resolution by station (for surface and DCM samples) indicating that micronutrients and local conditions play an important role in defining viral populations." Second, "We show for the first time that viral communities in the ocean are similar across temperature gradients, irrespective of their location in the ocean."

This treatment does not point out any contradiction to the previous study. The finding is not mentioned in the heading of the subsection, the caption of Table 1 about Tara run time, or the caption of Figure 5 about Tara results. The finding is not mentioned in the Title or in the Abstract or in the Innovations section. The finding appears to be based on a visual interpretation that is vague ("largely structured by temperature") and provided without statistics. Thus, the wording of the manuscript suggests that this finding was presented, not as a conclusion about the oceans, but as an example of how Libra can be used. In its guide for authors, GigaScience says, "Research Articles present work utilising large scale data that provide some scientific insight and conclusions" (<https://academic.oup.com/gigascience//pages/research>). With respect, I maintain that the revised manuscript is an Application Note and not a Research Article.

*RESPONSE: We thank the reviewer for their comments, and agree that the scientific findings are not the main focus of the paper. We ask that the editor consider our revision for an Application Note and not a Research Article.*

Secondly, I had noted that the manuscript makes 3 claims to innovation with insufficient support. In their response to reviewers, the authors added the qualification that their application of Hadoop was a first in metagenomics. However, the revised manuscript omits that qualification. After saying, "Libra presents three main innovations", the revised text claims (1) "the use of a scalable Hadoop framework enabling massive dataset comparison" is novel. This sentence does not include any first-in-metagenomics qualification. The claim is unsupported as written.

The revised text claims (2) "linear calculations for complex distance metrics allowing for high accuracy and clustering of the metagenomes based on their k-mer content" is novel. This sentence combines 6 ideas, leaving it unclear what precisely is being claimed. Is this the first linear-time calculation, or the first highly-accurate calculation, or the first k-mer based calculation, or some combination? I find this claim

unsupportable as written. The revised text claims (3) "a web-based tool imbedded in the CyVerse advanced cyberinfrastructure through iMicrobe for broader use of the tool in the scientific community" is novel. This claim has no first-in-metagenomics qualification. The claim is unsupported as written. With respect, I maintain that the revised manuscript's three claims to innovation are unproven.

*RESPONSE: We agree with the reviewer that each of these claims requires clarification and support based on previous work. The innovation we are trying to convey is in the end-to-end solution we provide rather than each component individually. We have carefully re-phased the abstract and “Innovations” section to clarify this important point. We also added more references and contrasts to previous related works.*

(1) *We changed the problematic first claim from “the use of a scalable Hadoop framework enabling massive dataset comparison” to “Libra is therefore the first k-mer based de-novo comparative metagenomic tool that uses rely on a Hadoop framework for scalability and fault tolerance”*
(2) *We changed the second claim from “linear calculations for complex distance metrics allowing for high accuracy and clustering of the metagenomes based on their k-mer content" to “Cosine similarity, although extensively used in computer science, has been rarely implemented in genomic and metagenomic studies* [*(Okuda et al. 2012)*](https://paperpile.com/c/8kIEbl/YE8x)*. To our knowledge, this work is the first to describe the use of the cosine similarity metric to cluster metagenomes based on their k-mer content. “*
(3) *We modified the last claim from "a web-based tool imbedded in the CyVerse advanced cyberinfrastructure through iMicrobe for broader use of the tool in the scientific community" to “The work described here is the first step in implementing a free cloud-based computing resource for de-novo comparative metagenomics that can be broadly used by scientists to analyze large-scale shared data resources.”*

A more thorough review might have been possible had Tracked Changes been presented.

*RESPONSE: We apologizes for the oversight. We have included the tracked changes in three supplemental documents. The first two were from the original re-submission. And the third revision highlights changes described here.*

Reviewer #3: Authors have partially addressed my concerns, otherwise several still apply:

The reference 4 that authors give for "Microbial dark matter" does not introduce anything about microbial dark matter. Typo ?

*RESPONSE: Thank you for catching this, we have updated to add three references specific to microbial dark matter and the role of metagenomics in expanding the tree of life*.

Also, note that it was not necessary to move table 1 to supplemental material -- I was hoping for some clarifications about it not more (cf. my previous comment) -- if authors move this table then they will make sure credits/citations are nonetheless fully given.

*RESPONSE: To streamline the introduction, we followed your initial suggestion to add the table to the supplemental. We have split the original table into two tables that are focused on the main points in the introduction. Supplemental Table 1A provides a comprehensive list of all de novo metagenomic comparison tools that we are aware of. Supplemental Table 1B provides a comprehensive list of all genomic/metagenomic tools that use a Hadoop framework for computation. The main point of Supplemental Table 1A is to show that Libra is the first de novo metagenomic comparison tool to use a Hadoop framework and also provide the user with a web-based tool. The main point of Supplemental Table 1B is to show that other genomic and metagenomic tools use Hadoop framework, but are for other use-cases. We have also made sure that each of the tools are cited in the main text.*

There is still the issue of the formatting for the equations/formulas/vectors, see "Cosine Similarity metric" or "Sweep line algorithm", some strange symbols are indicated (I opened this manuscript with different PDF readers, including Adobe, they all show formatting issues). Is it an issue by the editor/s platform or authors ?

*RESPONSE: Our apologies the conversion didn’t work properly again. We fixed by uploading the PDF of the manuscript (as a primary file), in addition to the docx (as Supplemental).*

Finally, "artificial" is still use in Supplemental Figure 1.

*RESPONSE: Thank you for finding this. We have updated Supplemental Figure 1 to remove the term “artificial”.*
